# Comparison of methodologies used to determine aromatic lignin unit ratios in lignocellulosic biomass

**DOI:** 10.1186/s13068-021-01897-y

**Published:** 2021-03-06

**Authors:** Renee M. Happs, Bennett Addison, Crissa Doeppke, Bryon S. Donohoe, Mark F. Davis, Anne E. Harman-Ware

**Affiliations:** 1 Renewable Resources and Enabling Sciences Center, National Renewable Energy Laboratory, Golden, CO 80401 USA; 2 Biosciences Center, National Renewable Energy Laboratory, Golden, CO 80401 USA

**Keywords:** Lignin, S/G ratio, Thioacidolysis, NMR, Pyrolysis-molecular beam mass spectrometry

## Abstract

**Background:**

Multiple analytical methods have been developed to determine the ratios of aromatic lignin units, particularly the syringyl/guaiacyl (S/G) ratio, of lignin biopolymers in plant cell walls. Chemical degradation methods such as thioacidolysis produce aromatic lignin units that are released from certain linkages and may induce chemical changes rendering it difficult to distinguish and determine the source of specific aromatic lignin units released, as is the case with nitrobenzene oxidation methodology. NMR methods provide powerful tools used to analyze cell walls for lignin composition and linkage information. Pyrolysis-mass spectrometry methods are also widely used, particularly as high-throughput methodologies. However, the different techniques used to analyze aromatic lignin unit ratios frequently yield different results within and across particular studies, making it difficult to interpret and compare results. This also makes it difficult to obtain meaningful insights relating these measurements to other characteristics of plant cell walls that may impact biomass sustainability and conversion metrics for the production of bio-derived fuels and chemicals.

**Results:**

The authors compared the S/G lignin unit ratios obtained from thioacidolysis, pyrolysis-molecular beam mass spectrometry (py-MBMS), HSQC liquid-state NMR and solid-state (ss) NMR methodologies of pine, several genotypes of poplar, and corn stover biomass. An underutilized approach to deconvolute ssNMR spectra was implemented to derive S/G ratios. The S/G ratios obtained for the samples did not agree across the different methods, but trends were similar with the most agreement among the py-MBMS, HSQC NMR and deconvoluted ssNMR methods. The relationship between S/G, thioacidolysis yields, and linkage analysis determined by HSQC is also addressed.

**Conclusions:**

This work demonstrates that different methods using chemical, thermal, and non-destructive NMR techniques to determine native lignin S/G ratios in plant cell walls may yield different results depending on species and linkage abundances. Spectral deconvolution can be applied to many hardwoods with lignin dominated by S and G units, but the results may not be reliable for some woody and grassy species of more diverse lignin composition. HSQC may be a better method for analyzing lignin in those species given the wealth of information provided on additional aromatic moieties and bond linkages. Additionally, trends or correlations in lignin characteristics such as S/G ratios and lignin linkages within the same species such as poplar may not necessarily exhibit the same trends or correlations made across different biomass types. Careful consideration is required when choosing a method to measure S/G ratios and the benefits and shortcomings of each method discussed here are summarized.

## Introduction

Lignin is an abundant aromatic polymer found in plant cell walls where it contributes to defense against insects and microbes, structural support of the plant, and water and nutrient transport [1]. Lignin is composed of aromatic units that vary in structure and composition depending on the plant species, tissue type, and specific cell wall layer [2, 3]. The lignin content in hardwood tree species generally ranges from 18 to 30% of the dry wood mass and varies in the ratio of aromatic lignin units. Hardwood lignin consists primarily of sinapyl or syringyl (S) phenylpropanoid units and coniferyl or guaiacyl (G) phenylpropanoid units and a small amount of *p*-coumaryl (H) phenylpropanoid units. Softwood tree species such as pines consist of lignins that are predominantly composed of G units, while grasses have lignin composed of S, G, and H units and also contain ferulates and coumarates in the cell walls. The S, G, and H canonical monolignols are used to form most lignin polymers in most types of biomass but other non-conventional aromatic units such as caffeyl alcohol, tricin, resveratrol and others occur in certain tissue types and biomass species as reviewed in del Rio et al. [4].

In the context of developing technologies to use plant biomass as a source of renewable feedstock for fuels and chemicals, there has been significant interest in how lignin content and composition facilitates or inhibits access to cell wall carbohydrates by cellulolytic enzymes, and utilization of the lignin itself [5, 6]. Increased delignification rates of hardwoods have been shown to correlate with increases in S/G ratios [7]. For example, higher S content in woody species has been correlated with greater pulping efficiency [8, 9]. These results have been suggested to be due in part to the lower abundance of carbon–carbon linkages among S units relative to other aromatic lignin units [10, 11]. Lignin content and S/G ratios could be critical biomass attributes in a biorefinery processes, particularly related to how these metrics impact enzymatic or acidic hydrolysis of biomass to produce fermentable sugars [12–14]. The relative abundance of lignin aromatic units has also been shown to correlate with anaerobic digestion of biomass to produce methane [15]. Thus, by breeding or by directly engineering the lignin biosynthetic pathway in biomass to alter S/G ratios, biomass conversion to bioproducts and biofuels can be made more efficient and economical [16].

Various methods can be used to determine the relative abundance of aromatic lignin units, particularly the ratio of the most abundant S and G units (S/G ratio). Thioacidolysis is a common chemical degradation method used to quantitatively determine the S/G ratio of non-condensed lignin units in various types of plants [17, 18]. Thioacidolysis proceeds by cleaving β-O-4 ether linkages, where additional steps such as Raney nickel desulfurization may be implemented to analyze dimers with C–C linkages [19, 20]. Variations in lignin structures may lead to differences in S, G, and H unit yields obtained from thioacidolysis [17]. For example, the higher occurrence of C–C linkages involving the 5-position of G units in softwood lignins may negatively impact thioacidolysis yields, while thioacidolysis yields may be higher from hardwood lignins containing more S units (in comparison to softwoods) bound by ether linkages [18, 21]. Observations that higher S/G ratio lignins correspond to a more effective release of total aromatic units have been previously reported [22–27]. However, recent work has indicated that the S/G ratio of poplar as determined by thioacidolysis is not correlated to the yield of aromatic units produced by reductive catalytic fractionation (RCF), a thermochemical conversion technique relevant to lignin characterization or lignin upgrading strategies [28].

Pyrolysis or thermal degradative methods coupled with chromatographic and/or mass spectrometry techniques can be performed on minimally processed biomass, yield highly reproducible results, and can be used in high-throughput platforms to analyze lignin content and composition in biomass [3, 29–33]. However, thermal degradation may overestimate the S content as products generated from labile ether linkages are detected whereas condensed linkages (either inherent or produced) may not be incorporated in the analyses, not unlike chemical degradation methods [34, 35]. Additionally, the S/G ratios measured by analysis of pyrolysis products do not always compare well with other degradative techniques, particularly as some phenolic pyrolysates may not originate from lignin (for example, there may be overlaps of G-lignin and ferulate-derived species) [36–41].

^1^H and ^13^C nuclear magnetic resonance (NMR) spectroscopy, in both solution or gel and solid-state, has been widely used to study the structure and composition of lignin [42, 43]. Additionally, ^31^P NMR has been employed as a method to investigate lignin structure by derivatization of hydroxyl groups in lignin subunits [44–46]. While this method is useful for analysis of certain functional groups, it requires extensive sample preparation and alteration of lignin, it only analyzes solubilized components, and is not necessarily capable of resolving S and G units [47].

2D heteronuclear (^1^H–^13^C) single quantum correlation (HSQC) spectroscopy is used to estimate S/G ratios from either isolated lignin or whole biomass among many different plant species, and alleviates some of the spectral overlap that occurs in 1D ^1^H and ^13^C methods; however, the method is not fully quantitative due to differential relaxation of the nuclei being analyzed [43, 48]. Whole cell wall analysis by NMR in a gel state can be performed by solubilizing biomass in various solvents, but these methods may suffer from incomplete solubilization, sample heterogeneity, and differences in relaxation times that can affect S/G ratio measurements [49–51]. 2D NMR methods may use correction factors, but this methodology resulted in lower S/G ratios determined for lignin in hardwood species than S/G ratios determined by chemical degradation methods [52]. More recently, quantitative 2D NMR methods such as HSQC_0_ [53] have been developed that addresses the relaxation problems associated with the previous methods to improve quantitation of aromatic lignin units [54]. The results from quantitative HSQC_0_ have shown lower S/G ratios for *Miscanthus* than the previously reported ratios measured by thioacidolysis (which only detects aromatic lignin units released from cleavage of β-O-4 linkages) [54, 55]. More recently HSQC_0_ has been employed to predict depolymerization yields from lignin [56]. Generally, NMR methods in use today accommodate either isolated lignin or whole biomass for analysis [42].

A method to measure S/G ratios of hardwoods, without the need for degradation or extensive sample preparation, was developed by Manders et al. using ^13^C solid-state NMR (ssNMR) [57] and versions of the method have been applied broadly [58–64]. The ^13^C ssNMR method determines the S/G ratio by subtracting a softwood lignin spectrum (softwood lignin consisting primarily of G units) from a hardwood spectrum. The obvious advantage of this approach comes from omitting the time-consuming lignin isolation step required in most chemical degradation methods and many spectroscopic methods, which may lead to enrichment of S units. However, the method developed by Manders assumes that the softwood G-lignin ^13^C ssNMR spectroscopic profile in the ~ 150 ppm range matches the G-lignin sub-profile of S- and G-rich hardwood, which may not be universally applicable across all biomass types [61]. As an alternative to the Manders method, spectral deconvolution of the aromatic region from quantitative or semi-quantitative ^13^C ssNMR data has been used to obtain lignin composition data [65–69].

The purpose of this manuscript is to provide a comparison of the S/G ratios determined for a series of biomass samples including pine (softwood), poplar (hardwood), and corn stover (grass) as determined by thioacidolysis, py-MBMS, gel-state HSQC NMR, and ^13^C ssNMR using two types of data analysis including a spectral deconvolution peak-fitting analysis to determine the relationships between analytical methodologies and the resulting aromatic lignin unit ratios and linkages determined in different types of biomass. The novelty of this work is the considered comparison of multiple deconstructive and non-destructive lignin analysis methods applied to multiple samples from a single biomass type (poplar). This enabled a unique comparison of characterization techniques and an evaluation of the understanding of lignin composition and structure provided by each technique. Additionally, softwood and corn stover samples were analyzed for lignin composition and structure to demonstrate how analytical limitations may vary among biomass types.

## Results

### Thioacidolysis

Thioacidolysis yields of aromatic lignin units for each of the biomass samples are presented in Table 1 (also Additional file 1: Table S1 with other S/G values determined by other methods). The Pearson correlation coefficient between the total yield of thioacidolysis-released aromatic lignin units on a g^−1^ of biomass basis and the yield of aromatic lignin units reported g^−1^ of estimated lignin content (based on py-MBMS analysis) was 0.93; therefore, comparison of yields on g^−1^ of estimated lignin content or a g^−1^ of biomass would be similar and are subsequently discussed on a g^−1^ of biomass basis as the lignin content was an estimate based on py-MBMS analysis. While the H unit content determined by thioacidolysis was consistent among the set of poplar samples, the S and G unit content varied widely, both in S/G ratio and total aromatic lignin unit yields. The S/G ratio determined by thioacidolysis did not correlate with the total yield of aromatic units (Pearson correlation = − 0.08, Additional file 2: Table S2). However, the yield of S units was the primary driver in the total yield of thioethylated aromatic lignin units for data both within the set of poplar samples (Pearson correlation of S, total units = 0.92, Pearson correlation of G, total units = 0.62) and including the corn stover (Pearson correlation of S, total units = 0.94, Pearson correlation of G, total units = 0.68, Additional file 3: Table S3). Thioacidolysis of pine produced only G and H-derived units, similar to previously reported values [18]. The corn stover sample yielded lower total aromatic lignin units from thioacidolysis than most of the hardwoods and softwood samples, which is typical for grasses [17, 18].

**Table 1:** Aromatic lignin unit content of biomass determined by thioacidolysis (average of *n* = 2 samples, not all samples were analyzed by thioacidolysis and are denoted N/A)

Biomass ID	H µmol/g biomass	S µmol/g biomass	G µmol/g biomass	S/G	Sum µmol/g biomass	Sum µmol/g lignin content*
NIST 8493 Pine	6.7	0.0	205.7	0.0	217.7	818.4
FCIC Corn Stover	4.8	47.5	37.9	1.3	97.2	709.5
BESC-004 Poplar	5.5	218.5	105.6	2.1	329.6	1277.5
BESC-021 Poplar	5.5	111.3	87.5	1.3	204.3	908.0
BESC-036 Poplar	5.4	207.3	69.4	3.0	282.0	1155.7
BESC-075 Poplar	4.5	78.1	22.3	3.5	104.9	476.8
BESC-095 Poplar	N/A	N/A	N/A	N/A	N/A	N/A
BESC-096 Poplar	N/A	N/A	N/A	N/A	N/A	N/A
BESC-140 Poplar	5.0	156.3	40.7	4.0	202.0	834.7
BESC-169 Poplar	5.1	144.8	85.5	1.7	235.3	1089.4
BESC-173 Poplar	N/A	N/A	N/A	N/A	N/A	N/A
BESC-182 Poplar	5.1	185.3	78.1	2.4	268.5	1214.9
BESC-217 Poplar	5.1	147.0	57.3	2.6	209.4	887.3
BESC-219 Poplar	N/A	N/A	N/A	N/A	N/A	N/A
BESC-255 Poplar	N/A	N/A	N/A	N/A	N/A	N/A
BESC-282 Poplar	5.0	119.5	87.1	1.4	211.5	1026.7
BESC-322 Poplar	N/A	N/A	N/A	N/A	N/A	N/A
BESC-334 Poplar	5.0	198.1	68.4	2.9	271.6	N/A
BESC-388 Poplar	4.8	132.2	104.7	1.3	241.8	1129.9
BESC-841 Poplar	5.1	231.4	68.3	3.4	304.8	1354.7
BESC-853 Poplar	N/A	N/A	N/A	N/A	N/A	N/A
BESC-863 Poplar	4.8	138.6	62.9	2.2	206.3	916.9
BESC-883 Poplar	4.8	189.1	60.4	3.1	254.3	1077.5

*Lignin content estimate based on py-MBMS analysis

### Py-MBMS

Py-MBMS analysis of the biomass samples present in sufficient quantity (not all samples were analyzed due to low mass availability) produced spectra consisting of ions derived from S, G, and H lignin units bound by various types of linkages. Lignin contents were determined based on methods described previously [29, 31, 32] in order to estimate Klason lignin content (wt %) using mean-normalized spectra to remove mass-dependent variation; S/G ratios were determined using unique (minimal-overlapping) ions of known origin that produced S/G ratios consistent with other methods in the literature [31, 70]. Traditional S/G ratios determined by py-MBMS are assumed as being derived from S, G, and H units where this method was established based on the results from NMR and thioacidolysis, but theoretically could include additional or alternative ions, particularly as some of the ions chosen to determine S/G do potentially originate from multiple sources to varying degrees.

Lignin content estimates of the poplar samples by py-MBMS ranged from 20.6 to 25.8 wt% lignin (Table 2). Traditional S/G ratios determined for the poplar samples using py-MBMS ranged from 1.3 to 2.2. Focusing on ions between 50 and 250 due to the nature of ions outside of that range originating primarily from noise and overlapping sources, the variance of the spectra was highest for *m/z* 138 (G-lignin), 151 (G-lignin, this ion may also originate from ferulate), 165 (S-lignin), and 181 (S-lignin), where () indicates origin of ion. The lignin content and S/G based on py-MBMS data were weakly correlated (Pearson correlation = 0.54) within the poplar samples. Lignin content as determined by py-MBMS did not strongly correlate with thioacidolysis yields for the entire poplar set either (Pearson correlation = 0.45, Additional file 2: Table S2). However, the majority of the poplar lignin estimates from py-MBMS did appear to correlate well with aromatic peak abundances from the peak-fit NMR data (values in Additional file 1: Table S1). The lignin content of the NIST 8493 Monterey pine was provided by the supplier based on Klason results to be 26.6 wt% lignin, and the lignin content of the FCIC corn stover was estimated as 13.7 wt% relative to a corn stover sample of known Klason content using py-MBMS spectra. Table 2 provides characterization data of the biomass samples based on py-MBMS analysis. The S/G ratio determined for the NIST pine was 0.2 since non-zero values of the ions otherwise derived from S units were observed in the spectra, but these values could not be differentiated from noise and fragment ions and because pine does not produce S-lignin, the S/G for NIST pine was assigned to 0. Because the type and amount of noise and/or fragmentation contributing to the abundance of these ions is not known or likely to be consistent for each of the biomass types, this value was not adjusted across the other samples.

**Table 2:** Py-MBMS characterization of lignin content and S/G ratios in select biomass samples (not all samples were analyzable by py-MBMS and are denoted N/A)

Biomass ID	S/G	Lignin content
NIST 8493 Pine	0.0*	26.6
FCIC Corn Stover	0.8	13.7
BESC-004 Poplar	1.9	25.8
BESC-021 Poplar	1.3	22.5
BESC-036 Poplar	2.1	24.4
BESC-075 Poplar	2.0	22.0
BESC-095 Poplar	N/A	N/A
BESC-096 Poplar	N/A	N/A
BESC-140 Poplar	2.0	24.2
BESC-169 Poplar	1.6	21.6
BESC-173 Poplar	N/A	N/A
BESC-182 Poplar	1.9	22.1
BESC-217 Poplar	2.0	23.6
BESC-219 Poplar	N/A	N/A
BESC-255 Poplar	N/A	N/A
BESC-282 Poplar	1.4	20.6
BESC-322 Poplar	N/A	N/A
BESC-334 Poplar	N/A	N/A
BESC-388 Poplar	1.4	21.4
BESC-841 Poplar	2.2	22.5
BESC-853 Poplar	N/A	N/A
BESC-863 Poplar	1.8	22.5
BESC-883 Poplar	2.1	23.6

*Adjusted value based on S-derived lignin ion intensities low value

### Gel-state NMR (HSQC)

Gel-state HSQCs were collected on all poplar samples, as well as corn stover and pine, according to Mansfield et al. [51]. Generally, S/G ratios were consistent over triplicate runs, although the percent error ranged from as low as 2% to as high as 16%. It is believed that this range in error is partly due to inconsistencies in how well individual samples formed a gel (visual observation) and could also possibly come from the milling process. Some samples demonstrated better “gelling” after being heated to 40 °C for data collection, whereas other samples were unaffected (visual observation, data not shown). The S/G ratio of the corn stover and poplar samples ranged from 0.9 to 2.4. The S/G ratios of the poplar samples as determined by HSQC correlated strongly with the S/G ratios as determined by thioacidolysis (although the range for thioacidolysis was broader) and py-MBMS (Pearson correlation, HSQC/thioacidolysis = 0.84, HSQC/py-MBMS = 0.96, Additional file 2: Table S2).

Lignin unit linkages in the poplar samples as determined by HSQC provided structural information to inform bias in methodologies used to measure S/G ratios. Interestingly, β-O-4 linkages as determined by HSQC did not correlate with thioacidolysis yields (Pearson correlation = 0.11) and did not correlate strongly with lignin content as determined by py-MBMS (Pearson correlation = 0.45), indicating that the total yields of aromatic lignin units as detected from thioacidolysis and by py-MBMS was not solely dependent on the abundance of those linkages in the biomass but is complicated by the other linkages as well. Additionally, the poplar β-O-4 linkages did not strongly correlate with S-lignin unit yields from thioacidolysis (Pearson correlation = 0.40) but did for py-MBMS ions typically derived from S-lignins (Pearson correlation for most S-derived ions ~ 0.6, Additional file 2: Table S2). However, β-O-4 linkages in the poplar set did more strongly correlate with S/G ratio as determined by py-MBMS (Pearson correlation = 0.71), and to a lesser degree with S/G as determined by thioacidolysis (Pearson correlation = 0.70) and HSQC (Pearson correlation = 0.61). There was a general weakly negative correlation of poplar β-O-4 linkages with G units (Pearson correlation, HSQC/thioacidolysis = − 0.51, HSQC/G units from py-MBMS ~ − 0.5). Table 3 lists the calculated S/G and bond content results of triplicate analyses of poplar, pine, and corn stover samples.

**Table 3:** HSQC calculated S/G ratios and linkage content

Biomass ID	S/G ratio	S/G error	β-O-4	Error	β-β	Error	β-5	Error
NIST 8493 Pine	N/A	N/A	57	N/A	10	N/A	33	N/A
FCIC Corn Stover	1.1	0.20	99	N/A	N/A	N/A	N/A	N/A
BESC-004	1.6	0.15	70	2.0	24	1.4	6.1	1.2
BESC-021	0.89	0.04	66	2.6	25	2.4	9.4	1.5
BESC-036	1.9	0.17	74	3.8	21	4.1	4.8	2.1
BESC-075	2.1	0.05	72	2.3	23	2.1	4.6	0.4
BESC-095	1.5	0.21	75	2.6	20	1.2	5.2	2.4
BESC-096	2.1	0.25	76	2.3	20	2	3.4	0.9
BESC-140	1.7	0.11	74	4.2	22	2.8	4.3	1.5
BESC-169	1.2	0.05	73	1.9	21	0.5	6.5	2.1
BESC-173	1.1	0.13	69	2.1	23	1.1	8.2	1.0
BESC-182	1.8	0.20	73	4.1	21	3.7	5.9	2.3
BESC-217	1.7	0.18	72	0.5	23	2.1	5.3	1.8
BESC-219	2.3	0.13	72	1.8	25	3.1	2.8	1.6
BESC-255	1.8	0.17	71	3.4	22	3.0	6.2	1.0
BESC-282	1.1	0.04	62	3.7	25	2.6	14	4.8
BESC-322	2.2	0.19	75	2.4	22	1.9	3.2	0.8
BESC-334	2.3	0.21	69	2.3	27	1.4	4.5	1.0
BESC-388	0.94	0.01	67	4.4	22	2.4	11	3.1
BESC-841	2.4	0.30	73	5.7	23	5.9	3.8	1.8
BESC-853	2.4	0.33	73	3.8	23	1.6	3.3	2.4
BESC-863	1.4	0.12	73	4.3	23	2.9	4.1	1.7
BESC-883	2.3	0.36	76	1.8	21	2.9	2.5	1.4

### Solid-state NMR—Manders subtraction method

The S/G ratios of poplar samples were calculated from solid-state ^13^C NMR interrupted decoupling spectra as described by Manders [57]. Table 4 gives the integral values and the S/G ratios calculated using the Manders method for the poplar samples. A low, mid-range, and two high S/G ratio samples (as calculated by py-MBMS) were run in triplicate to determine consistency of the Manders method. Generally, S/G ratios were consistent over triplicate runs, with the percent error ranging from 4 to 7%. Overall, the S/G ratios calculated trend much lower than all the other methods studied thus far and had a significantly lower range of S/G ratios. The S/G ratio of the corn stover and poplar samples ranged from 0.5 to 1.1. The S/G ratios of the poplar samples as determined by ssNMR still did correlate with the S/G ratios as determined by thioacidolysis, py-MBMS or HSQC (Pearson correlation, ssNMR/thioacidolysis = 0.76, ssNMR/py-MBMS = 0.81, ssNMR/HSQC = 0.80).

**Table 4:** Integration values of interrupted decoupling ssNMR spectra (Manders method)

Biomass ID	S integral	G integral	Normalized S	Normalized G	S/G
NIST 8493 Pine	N/A	100.0	N/A	33.3	N/A
FCIC Corn Stover	37.6	62.4	9.4	20.8	0.5
BESC-004	48.1	51.9	12.0	17.3	0.7
BESC-021*	46.4	53.6	11.6	17.9	0.7
BESC-036	52.3	47.7	13.1	15.9	0.8
BESC-075	53.2	46.8	13.3	15.6	0.9
BESC-095	52.8	47.2	13.2	15.7	0.8
BESC-096	59.2	40.8	14.8	13.6	1.1
BESC-140	54.8	45.2	13.7	15.1	0.9
BESC-169	43.7	56.3	10.9	18.8	0.6
BESC-173	47.5	52.5	11.9	17.5	0.7
BESC-182*	48.8	51.2	12.2	17.1	0.7
BESC-217	54.0	46.0	13.5	15.3	0.9
BESC-219	59.0	41.0	14.7	13.7	1.1
BESC-255	53.4	46.6	13.4	15.5	0.9
BESC-282	43.1	56.9	10.8	19.0	0.6
BESC-322	50.9	49.1	12.7	16.4	0.8
BESC-334	59.8	40.2	15.0	13.4	1.1
BESC-388*	45.7	54.3	11.4	18.1	0.6
BESC-841	54.6	45.4	13.7	15.1	0.9
BESC-853*	55.8	44.2	14.0	14.7	0.9
BESC-863	50.1	49.9	12.5	16.6	0.8
BESC-883	59.7	40.3	14.9	13.4	1.1

*Average of duplicate or triplicate sample runs

### Solid-state NMR—spectral deconvolution

Spectral deconvolution (peak-fitting) of ^13^C CP-MAS spectra in the aromatic domain was performed to estimate the relative abundances of S and G and lignin for twenty-two natural poplar variants and one corn stover sample. Peak-fitting ^13^C solid-state NMR data to quantitatively understand local molecular structure of biopolymers is a widely used practice, with many applications to biomass and other heterogeneous polymers [67, 71, 72]. Solid-state NMR profiles are generally deconvoluted into pseudo-Voigt lineshapes, meaning a weighted sum of Gaussian and Lorentzian profiles are used for a single resonance. This means any particular ^13^C NMR resonance has variables in peak position, amplitude, width (full-width half-max, or FWHM), and finally peak shape (Gaussian vs Lorentzian weighting factor). When deconvoluting overlapping resonances, inaccurate initial estimates of peak position, linewidth and peak shape might result in unreliable and inaccurate fits.

**Fig. 1:**
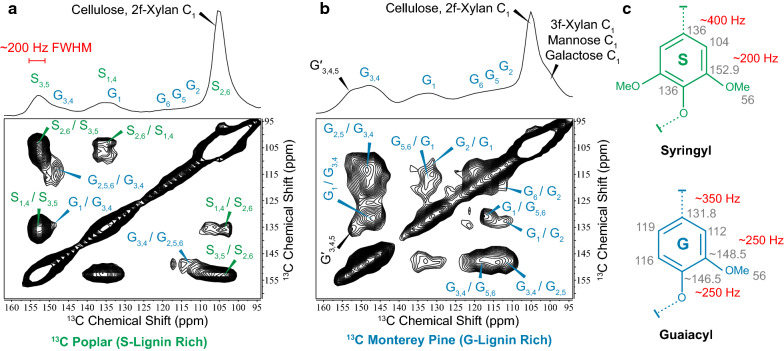
Aromatic region of through-space two-dimensional ^13^C–^13^C correlation data on **a** ^13^C-enriched poplar woody stems and **b** ^13^C-enriched Monterey pine. ^13^C chemical shifts and approximate linewidths were extracted from the data and listed in grey and red text next to aromatic lignin unit chemical structures shown in **c**

**Fig. 2:**
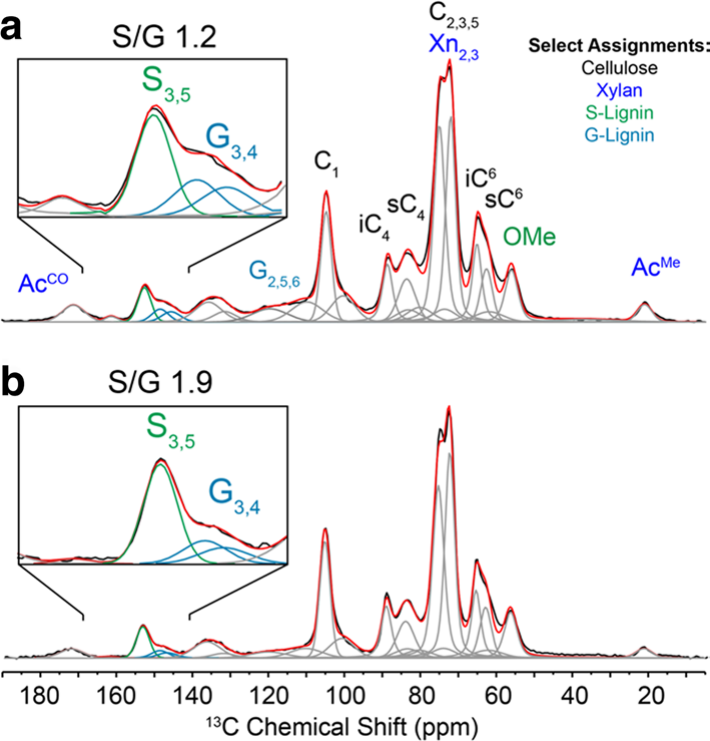
Example spectral deconvolution of ^1^H–^13^C CP-MAS data obtained from low-S (**a**) and high-S (**b**) natural poplar variants. Lignin S/G ratios were obtained from deconvoluted peak areas from lignin S_3,5_ and G_3,4_ signals located near 153 and 146–148 ppm, respectively

To identify acceptable initial fitting conditions prior to spectral deconvolution, we collected 2D ^13^C–^13^C through-space dipolar assisted rotational resonance (DARR) spectra on model ^13^C-enriched woody biomass (Fig. 1). Precise ^13^C chemical shifts were extracted from inspection of off-diagonal cross-peaks, and reasonable linewidth estimates were obtained from analysis of resolved cross-peaks. S-lignin peaks were identified from S-lignin-rich ^13^C-enriched hybrid poplar woody stems (Fig. 1a), while G-lignin shifts were extracted from ^13^C-enriched Monterey pine since softwood biomass is entirely G-lignin (Fig. 1b). We note that two signals centered at 146.5 and 148.5 could be identified in the 2D spectrum, which we use to represent G_3,4_ moieties generally. Since guaiacyl units lack a methoxy group at the ring-5 position and are therefore subject to carbon–carbon and carbon–oxygen condensation, a broader distribution of chemical environments is expected for G units compared to the more symmetric S units. Minor G-lignin chemical shifts in the poplar biomass were consistent with G-lignin signals in the pine sample. Peak shapes were initially set to mixed 90% Gaussian 10% Lorentzian component because sample heterogeneity will impart a Gaussian distribution of Lorentzian-like signals. However, this weighting factor was varied systematically (from 9:1 to 1:9) to help identify fitting errors. To improve these initial starting parameters, a single CP-MAS spectrum representative of the full dataset was deconvoluted such that chemical shifts were only allowed to perturb by 0.1 ppm about the shifts identified from the 2D data, and peak widths and lineshapes were allowed minor deviation from initial guesses to obtain optimal fits. Finally, with initial chemical shifts, peak widths and peak shapes all carefully estimated, batch-fitting of the entire dataset was accomplished using Python code (fitting performed with lmfit module) in which only peak amplitude was allowed to vary for each spectrum whereas peak position, Gaussian/Lorentzian ratios and FWHM were locked for all samples. Representations of resulting spectral deconvolutions for High-S (BESC-096) and Low-S (BESC-021) lignin natural poplar variants are shown in Fig. 2 (fits for all samples are provided in Supplementary Materials). S/G ratios arise directly from the relative deconvoluted peak areas of the S_3,5_ to G_3,4_ signals.

When applying this fitting strategy, it became clear that small variations in the initial starting conditions had minor effects on the final observable, namely S/G ratios based on the 153/148 ppm peak areas. Since estimation of initial fitting parameters were obtained with a manual process and are therefore subject to some researcher bias, the above procedure was repeated several times. Table 5 lists the average S/G ratios obtained from repeated batch-fitting of the entire poplar and corn stover dataset using a range of different starting conditions.

**Table 5:** S/G ratios obtained from peak-fitting for deconvolution of ssNMR (n = 7 fitting iterations)

Biomass ID	Average S/G	Std Dev
FCIC Corn Stover	0.74*	0.04
BESC-004	1.6	0.1
BESC-021	1.2	0.1
BESC-036	2.0	0.2
BESC-075	2.1	0.2
BESC-095	1.7	0.1
BESC-096	2.0	0.2
BESC-140	1.9	0.2
BESC-169	1.4	0.1
BESC-173	1.4	0.1
BESC-182	1.7	0.1
BESC-217	1.9	0.1
BESC-219	2.1	0.2
BESC-255	2.1	0.2
BESC-282	1.3	0.1
BESC-322	2.2	0.2
BESC-334	2.2	0.2
BESC-388	1.4	0.1
BESC-841	2.1	0.2
BESC-853	2.1	0.2
BESC-863	1.6	0.1
BESC-883	2.2	0.2

*Ratio measured = S/(G + FA)

## Discussion

### Limitations, considerations, and the impact of cell wall structure on S/G and associated methodologies

**Fig. 3:**
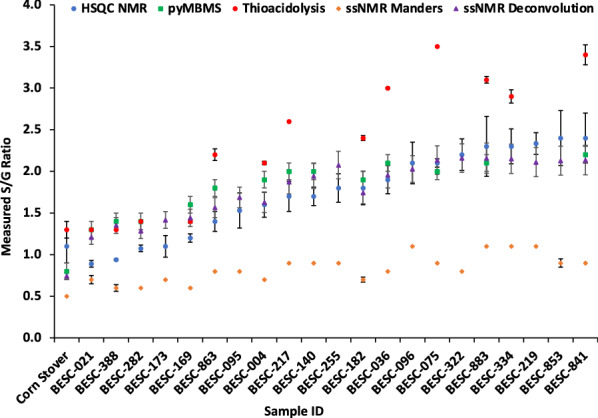
Comparison of S/G ratios in corn stover and poplar measured, calculated, or predicted by thioacidolysis, py-MBMS, HSQC, ssNMR Manders method, and ssNMR spectral deconvolution. Samples are sorted for graphical viewing by listing corn stover first, and sorting poplar samples via HSQC measured S/G ratios. Error bars are provided where duplicate or triplicate sample analysis was performed

A comparison of S/G ratios determined by four different methods for the poplar biomass analyzed is provided in Fig. 3 and Additional file 1: Table S1. Generally, thioacidolysis analyses resulted in higher S/G ratios, whereas the Manders ssNMR method plateaued at 1.1. HSQC and py-MBMS data yielded similar S/G ratios for the poplar and corn stover samples. The deconvoluted ssNMR spectra provided S/G measurement improvements over the Manders method to more closely reflect the S/G values determined by HSQC and py-MBMS.

Lignin is not evenly distributed within a plant. Past studies have shown that almost 75% of the lignin in hardwood is contained within fiber cells and only 25% of lignin is contained in vessel and ray cells [73]. Lignin concentration is also highest in the middle lamella of the fiber cells, while the secondary cell walls contain a higher total amount of lignin [21, 74]. High concentrations of G units are present in the middle lamella, which may be more condensed, containing a higher proportion of the total C–C linkages present that would not be released during thioacidolysis. Since S-type lignin tends to be dominant in the thicker, less dense secondary cell walls and would contain more β-O-4 linkages, these S units could be released more effectively. One can reasonably assume that G units may not be effectively released even at low S/G ratios. This could explain why the S/G ratio measured by thioacidolysis trends high, agreeing with previous observations regarding chemical degradation methods [23, 25, 27].

Similarly, since py-MBMS is only capable of releasing and detecting aromatic lignin units and dimers after breaking thermally labile linkages, it may be biased towards the analysis of moieties bound by β-O-4 linkages, particularly S-lignin units. If S/G ratios measured by py-MBMS were heavily impacted by and correlated with β-O-4 linkages, in addition to being positively correlated with S content, then the total lignin content estimated would also be influenced such that the higher lignin content would trend with S/G. Here, the lignin content determined by py-MBMS did not correlate strongly with S/G nor did lignin content estimates correlate strongly with thioacidolysis yields for the poplar samples or the entire biomass sets. Therefore, in addition to strong correlation with HSQC S/G, which may be less biased but is otherwise only semi-quantitative, py-MBMS S/G determined by the traditional method could be an accurate representation of S/G ratios in biomass, pending other linkage or aromatic unit anomalies not explored here that may otherwise impact the data. However, given the relatively small sample size of this data set and since the lignin values determined by py-MBMS did not consistently align with solid-state aromatic NMR spectral features, it may be necessary to interpret some of these correlations with caution.

Several factors contribute to the error associated with the S/G ratios measured by ^1^H–^13^C HSQC volume integrations. HSQC experiments are generally not quantitative due to the way the experiment is typically performed. Cross-peaks in a ^1^H–^13^C HSQC experiment arise from polarization transfer through one-bond J-coupling to correlate protons directly bonded to a carbon. While this provides quantitative information, the intensity of the cross-peaks is impacted by both heteronuclear and homonuclear coupling constants as well as T_1_ and T_2_ relaxation. A single ^1^H–^13^C HSQC experiment is generally optimized for one ^1^H–^13^C coupling constant; usually an average value of 145 Hz is chosen to capture both aromatic (~ 160 Hz) and aliphatic (~ 120 Hz) environments in an effort to optimize the number and intensity of the observed cross-peaks. Additionally, relaxation is not accounted for in the same way as 1D experiments, where a delay of 5 × T_1_ is employed. These delays make an HSQC experiment untenably long. The use of modern adiabatic pulse sequences mitigates most of the issues with J-coupling, but care should still be taken when using a single HSQC experiment to quantify both aromatic lignin units and inter-unit bond linkages, as the latter falls in the aliphatic region. While adiabatic HSQC pulse sequences were employed here, the experiment is considered semi-quantitative for the samples studied here, meaning only relative amounts of lignin units or linkages can be compared between samples.

### ^*13*^*C ssNMR analysis of lignocellulosic biomass cell walls*

Solid-state NMR data were processed in two different ways (Manders et al. and spectral deconvolution or peak-fitting) to extract S/G estimates directly from the intact biomass. Results from both data processing methods trend correctly, but it appears the Manders subtraction method undercounts S/G ratio compared to spectral deconvolution. Like thioacidolysis, py-MBMS, and gel-state HSQC analyses, there are issues related to analyses of S/G by solid-state ^13^C NMR methods. First, broad overlapping lines and overall poor resolution of 1D ^13^C solid-state NMR data poses inherent challenges, especially if detailed analyses of lignin composition are desired. Second, due to the low sensitivity of NMR in general, compounded with the low (1.1%) natural abundance of ^13^C, a single CP-MAS experiment usually requires long acquisition times. Sample throughput cannot possibly compete with some other analytical techniques used to obtain S/G estimates. That said, with the advancement of dynamic nuclear polarization (DNP)-enhanced solid-state NMR, which uses microwave irradiation to transfer spin polarization from electron radicals to nearby nuclei, it may be possible to perform these measurements in under 5 min per sample [75]. Additionally, it is widely known that routine cross-polarization NMR data is not inherently quantitative. In addition to experimental choices like magnetic field strength and rotor spinning speed, differences in cross-polarization rates (T_CH_), spin–lattice relaxation times (T_1_), and spin–lattice relaxation times in the rotating frame (T_1ρ_) for carbons in different chemical environments can affect their relative intensities [76, 77]. However, in our experiments, differences in CP rates can be neglected because (1) we operated at a reasonably low (50 MHz) ^13^C Larmor frequency and low (6900 Hz) spinning speed such that the CP condition was quite robust, and (2) the S- and G-lignin carbons of interest used for analysis (respectively, at 153 and 148 ppm) are both non-protonated quaternary aromatic carbons in similar dynamic environments. In support, identical correction factors to adjust for variations in cross-polarization kinetics were found by Davis et al. for these two signals, confirming this assessment [68]. Therefore, careful experimental setup and data analysis can ensure that reproducible S/G ratios are determined by counting all S and G units using the solid-state ^13^C NMR method. That said, researchers operating at higher magnetic fields and faster spinning speeds may need to take more precautions; in cases where routine ramped CP-MAS might be insufficient, the multiCP method developed by the Schmidt-Rohr lab would be an excellent approach [78].

It is clear from comparing S/G ratios determined using the Manders method with spectral deconvolution that the procedure in which the data is processed can significantly impact results. To explain the difference, we hypothesize that the method proposed by Manders of subtracting out the softwood-derived G-lignin profile from S- and G-rich hardwood spectrum is somewhat flawed. As can be seen in Fig. 1b, in softwoods there exists a minor downfield shoulder in the G-lignin spectrum near 153 ppm. Based on multiple reports in the literature [42, 70, 79–81] this signal can be assigned to G-lignin ring carbons at the 3, 4 and even 5 position depending on if the guaiacyl unit is or is not etherified, type of inter-unit linkage present and if carbon–oxygen condensation has occurred at the C5 position (labeled G′_3,4,5_ in Fig. 1b). The abundance of the G-lignin downfield shoulder near 153 ppm seen in pure-G softwoods is therefore unlikely to match the same G-lignin profile in S- and G-rich hardwoods. As a result, subtracting a G-lignin profile from hardwood ^13^C data could unintentionally subtract signal that is truly from S-lignin. This likely explains why S/G ratios obtained using the Manders method tend to under-represent syringyl units, while deconvolution of the same data produce S/G ratios that are consistent with HSQC and py-MBMS methods.

While promising, spectral deconvolution of ^13^C ssNMR data may not be broadly applicable to all biomass types. Lignin from hardwoods is predominantly S and G with low abundance of H units and *p*-hydroxybenzoates. Therefore, the aromatic region of the ^13^C NMR spectrum of hardwoods like poplar is reasonably simple making spectral deconvolution straightforward. On the other hand, corn stover is known to be rich in S and G units with an abundance of hydroxycinnamates such as ferulate (FA) and *p*-coumarate (pCA). The S/G ratios determined from deconvolution of the 148–153 ppm region likely overestimate guaiacyl content since FA and pCA moieties have spectral features near 147 ppm.

Similarly, some biomass types have significant representations of non-conventional aromatic lignin units derived from flavonoids (tricin), hydroxystilbenes, and acetylated lignin units, which may be shifted by 1–2 ppm compared to non-acetylated lignin units [4]. In other words, the spectral deconvolution approach demonstrated applied here may work to characterize a specific type of biomass (hardwoods are particularly promising) but care must be taken when applying across different biomass types. This concept is highlighted in our attempt to deconvolute ^13^C ssNMR spectrum from corn stover. A ratio between the 153 and 147 ppm regions of 0.7 to 0.8 was observed similar to py-MBMS (S/G = 0.7), but this does not match the S/G ratio of 1.1 measured from HSQC integrations or 1.3 as measured by thioacidolysis (Fig. 3). When FA content is considered, the S/(G + FA) ratios obtained from HSQC integrations (0.7, data not shown) and solid-state NMR peak-fitting analysis are consistent, confirming that G and FA content cannot be separately quantified in CP-MAS data and that the py-MBMS S/G analysis of grasses is also complicated by the presence of ferulates.

Despite these limitations, solid-state NMR methods are powerful for biomass characterization because they are rich in structural information and data is obtained on samples in their native and unaltered states. For example, in addition to S/G ratios shown here, estimates for cellulose crystallinity index and lignin composition are accessible from the same CP-MAS data [82–84].

In choosing a methodology for studying lignin and particularly if evaluation of S/G ratios is required, the first criterion to consider is size of the sample set. Large sample sets where reliable high-throughput data is required are suitable for py-MBMS or potentially HSQC, but not necessarily for thioacidolysis due to laborious sample preparation or for ssNMR due to limitations in equipment for handling large sample populations as well as experimental length unless DNP hardware is available. Additionally, if information beyond S/G ratios is needed, HSQC spectra can provide bond linkage information as well as other aromatic moieties present in lignin which becomes important for grassy species that are high in coumarates and ferulates. Additionally, py-MBMS could also provide lignin content estimates or thioacidolysis total aromatic lignin unit yields may also be suitable methods if those metrics are needed, although thioacidolysis has the distinct characteristic of only cleaving β-O-4 bonds and data will be biased accordingly. Py-MBMS should only compare lignin content of similar biomass types as well and comparing S/G across biomass types by py-MBMS may need to be interpreted with caution, particularly if comparing samples that may vary in the abundance of ferulates, which may produce similar pyrolysates and subsequent ions as G units. If amount of sample is limited, then non-destructive methods may offer the best alternative, although care needs be taken when employing spectral deconvolution to estimate S/G ratios and it is recommended for use only by those with experience in spectral deconvolution. Both HSQC and ssNMR provide a look at intact cell walls, and while it could be argued that the ball-milling required for HSQC may affect cell wall structure, it may be negligible so long as overheating is prevented and samples are milled consistently. Table 6 summarizes the main considerations for each methodology reported here.

**Table 6:** Benefits and shortcomings associated with methods used for S/G analysis reported in this work

Method	Benefit		Shortcoming
Thioacidolysis	Small sample size, high reproducibility, wealth of historical data		Potential bias for aromatic lignin units released by β-O-4 linkages not being representative (although can be advantage for specificity), sensitive to other components in biomass impacting reaction, laborious sample preparation
Py-MBMS	Small sample size, rapid analysis, high reproducibility, multiple cell wall phenotype measurements possible		Destructive, requires comparison within species, potential bias for aromatic lignin units released by thermally labile linkages, overlap of some lignin-derived ions with non-lignin-derived species, semi-quantitative
^1^H–^13^C HSQC	Representative of whole cell wall, multiple cell wall phenotype measurements possible particularly including lignin linkage information		Semi-quantitative, large sample size requirement, long analysis time (which can be overcome if cryoprobe is available)
ssNMR Manders	Non-destructive		Underestimates contribution from S-lignin units
ssNMR Deconvolution	Non-destructive, representative of whole cell wall, multiple cell wall phenotype measurements possible		Low throughput (can be overcome if DNP hardware is available), sensitive to incorrect initial peak-fitting parameters, not appropriate for grass species, need other a priori data

## Conclusions

Each of the methods considered here has its advantages and disadvantages for analyzing lignin in biomass. Choosing the best method will depend on the type of biomass being studied and whether comparisons are being made within or among different biomass species. If S/G ratios are being compared within species (for example, different phenotypes of poplar), methods such as HSQC, ^13^C deconvoluted/peak-fit ssNMR and py-MBMS, and thioacidolysis may provide sufficient, representative, comprehensive, and accurate information for lignin analysis studies. ^13^C ssNMR deconvolution method for S/G analysis may not provide an accurate analysis for grass species. Comparing S/G across species (for example, grasses and hardwoods) may best be interpreted using HSQC data to take in context the lignin linkage information also captured. S/G comparisons across species are also traditionally performed using thioacidolysis and py-MBMS but if lignin linkages differ substantially, bias in S/G may be captured using these methods. This study did not identify a significant correlation between lignin S/G ratio and β-O-4 linkages across these poplar samples. The methods used for comparing the S/G ratios determined within and across different types of biomass should be carefully considered in the context of the limitations of the methods and their resulting findings relationships with other lignin structural or linkage features.

## Methods and materials

### Biomass

*Populus trichocarpa* biomass was grown as outlined in Table 7 and samples were dried and debarked, milled, destarched and extracted with ethanol/water prior to analysis [33]. Corn stover was harvested and baled in October 2017 in Story County, Iowa. Bales were stored field-side for two months until being transported and placed in covered storage. A *Pinus radiata* (Monterey pine) sample used to represent a predominately guaiacyl-based lignin was obtained from the National Institute of Standards and Technology (NIST). ^13^C-enriched hardwood (DN34 poplar) and softwood (*Pinus radiata,* Monterey Pine, IsoLife) samples were also used to inform spectral deconvolution method development. Samples were extracted in a Soxhlet extractor using an ethanol solution and then dried under vacuum for 12 h.

**Table 7:** Samples used for lignin analysis. Samples were chosen based on a range of phenotypes including S/G ratios as determined by py-MBMS

Sample	Biomass species	Source
Pine	*Pinus radiata* (Monterey Pine)	NIST standard 8493
^13^C-enriched pine	*Pinus radiata* (Monterey Pine)	IsoLife
FCIC Corn stover	*Zea mays*	Iowa, USA
BESC-021	*Populus trichocarpa*	Pacific Northwest, USA
BESC-036	*Populus trichocarpa*	Pacific Northwest, USA
BESC-075	*Populus trichocarpa*	Pacific Northwest, USA
BESC-095	*Populus trichocarpa*	Pacific Northwest, USA
BESC-096	*Populus trichocarpa*	Pacific Northwest, USA
BESC-140	*Populus trichocarpa*	Pacific Northwest, USA
BESC-169	*Populus trichocarpa*	Pacific Northwest, USA
BESC-173	*Populus trichocarpa*	Pacific Northwest, USA
BESC-182	*Populus trichocarpa*	Pacific Northwest, USA
BESC-217	*Populus trichocarpa*	Pacific Northwest, USA
BESC-219	*Populus trichocarpa*	Pacific Northwest, USA
BESC-255	*Populus trichocarpa*	Pacific Northwest, USA
BESC-282	*Populus trichocarpa*	Pacific Northwest, USA
BESC-322	*Populus trichocarpa*	Pacific Northwest, USA
BESC-334	*Populus trichocarpa*	Pacific Northwest, USA
BESC-388	*Populus trichocarpa*	Pacific Northwest, USA
BESC-841	*Populus trichocarpa*	Pacific Northwest, USA
BESC-853	*Populus trichocarpa*	Pacific Northwest, USA
BESC-863	*Populus trichocarpa*	Pacific Northwest, USA
BESC-883	*Populus trichocarpa*	Pacific Northwest, USA

### Thioacidolysis

Thioacidolysis was performed on 2 mg of ground sample that was previously destarched and extracted, as reported in Harman-Ware et al. [18]. Samples were analyzed and yields were quantified by GC/MS using internal standard response factors built from external calibration standards of synthesized H, G and S arylglycerol species as reported in [85].

### Py-MBMS

Approximately 4 mg of ground biomass samples were pyrolyzed using a Frontier PY2020 unit at 500 °C for 30 s in 80 µL deactivated stainless steel cups and analyzed in duplicate. Mass spectral data was acquired using an Extrel Super-Sonic MBMS Model Max 1000 and processed using Merlin Automation software (V3). Spectra were collected from *m/z* 30 to 450 at 17 eV. Lignin content was estimated in a poplar sample set relative to NIST standard 8492 *Populus deltoides* of known Klason lignin content and corn stover lignin content was estimated relative to a reference corn stover material of known Klason lignin content. Ion intensities *m/z* 120, 124 (G), 137 (G), 138 (G), 150 (ferulate and G), 152, 154 (S), 164 (G), 167 (S), 168 (S), 178 (G), 180, 181, 182 (S), 194 (S), 208 (S) and 210 (S) where G denotes primarily guaiacyl-derived ions, S denotes primarily syringyl-derived ions, where other ions derived from other aromatic lignin units or multiple sources, were mean-normalized and summed to obtain relative lignin contents. Syringyl-to-guaiacyl (S/G) ratios were estimated by dividing the sum of S-based ions by the sum of G-based ion intensities.

### Whole biomass gel-state ^1^H–^13^C HSQC

Heteronuclear single quantum coherence (HSQC) NMR spectra were acquired for ball milled whole biomass samples (30–50 mg) dissolved in 500 µL DMSO-d6 and pyridine-d5 (4:1, 500 μL). Spectra were acquired at 40 °C on a Bruker Avance III 600 MHz spectrometer at 11.7 T using a room-temperature broadband probe. Spectra were acquired with 1024 points and a SW of 12 ppm in the F2 (^1^H) dimension and 256 points and SW of 220 ppm in the F1 (^13^C) dimension. The spectral processing parameters from Mansfield et al. [51] were used and integrations were performed using TopSpin 3.6. Spectra for each sample are provided in the Additional file 3: Materials.

### Solid-state ^13^C NMR

High-resolution, solid-state ^13^C NMR spectra were collected at 4.7 T with cross-polarization (CP) and magic angle spinning (MAS) in a Bruker Avance 200 MHz spectrometer. Interrupted decoupling spectra were obtained with 2.0 ms of variable amplitude CP to minimize intensity variations of the non-protonated aromatic carbons that are sensitive to Hartmann-Hahn mismatch at higher MAS rotation rates [86]. ^1^H and ^13^C fields were matched at 53.6 kHz and a 1 dB ramp was applied to the proton radiofrequency (r.f.) during the matching period. Proton decoupling began 30 μs after the conclusion of CP. Simultaneous 90-degree refocusing pulses were applied to both nuclei to refocus any evolution caused by chemical shift interaction. This removed baseline distortions due to phasing errors. An additional 30 μs delay for interrupted decoupling and refocusing was then applied. Acquisition time was 0.026 s with a spectral width of 20 kHz. MAS was performed at 6900 Hz with 40,000 scans averaged using a pulse repetition rate of 1.0 s. All ^13^C chemical shifts were referenced externally to TMS at 0.0 ppm by setting the downfield resonance of Adamantane to 38.48 ppm. To calculate solid-state ^13^C NMR S/G ratios for the poplar samples, the standard pine spectrum was scaled until the intensity of the shoulder at 148 ppm was equal to the intensity of the 148 ppm shoulder in the hardwood spectrum as described by Manders [56]. The softwood spectrum was then subtracted from the poplar spectrum and the result, theoretically, is a pure S spectrum. The S spectrum is then integrated from 158 to 126 ppm and calibrated against the softwood spectrum which was integrated in the same manner and calibrated to 100. The G component was calculated by multiplying the softwood integration by the scaling factor from the subtraction. These values are then normalized to an equal number of unprotonated units on the benzene ring (divide S intensity by 4, divide G intensity by 3) before calculating the S/G ratio. We assume that intensity arising from 5–5 linkages has a negligible interference with the S/G ratio calculation due to their low abundance, 5% or less, in hardwood lignin [21, 56]. Spectral deconvolution of CP-MAS data were informed by extracting ^13^C chemical shifts and linewidths from two-dimensional (2D) ^13^C–^13^C dipolar assisted rotational resonance (DARR) data on ^13^C-enriched hardwood (DN34 Poplar) and softwood (Monterey Pine, IsoLife) samples [87]. Spectra for each sample are provided in the Additional file 4: Spectral data.

## Supplementary Information

**Additional file 1.** S/G ratios determined by different methods, thioacidolysis yields, lignin content estimates, relative lignin linkage abundances and py-MBMS spectral peak TIC-normalized intensities for each sample analyzed in this study.

**Additional file 2.** Pearson correlation coefficients for various characteristics measured in the poplar samples.

**Additional file 3.** Pearson correlation coefficients for various characteristics determined using all samples in this study.

**Additional File 4.** 2D 1H -13C HSQC spectra and 1D 13C CP MAS Interrupted Decoupling spectra with spectral deconvolution peak fitting results of biomass samples.

## Data Availability

The Department of Energy will provide public access to these results of federally sponsored research in accordance with the DOE Public Access Plan (http://energy.gov/downloads/doe-public-access-plan). Data are available in the manuscript and any data not provided are available upon request from the coauthors.
