# Supplementary material for: Comparison of methodologies used to determine aromatic lignin unit ratios in lignocellulosic biomass
Source: Biotechnol Biofuels. 2021 Mar 6;14:58. doi: 10.1186/s13068-021-01897-y (PMC7936455; doi:10.1186/s13068-021-01897-y)
Supplement: Supplementary file 4 — Additional File 4. 2D 1H -13C HSQC spectra and 1D 13C CP MAS Interrupted Decoupling spectra with spectral deconvolution peak fitting results of biomass samples. [file 13068_2021_1897_MOESM4_ESM.pdf]

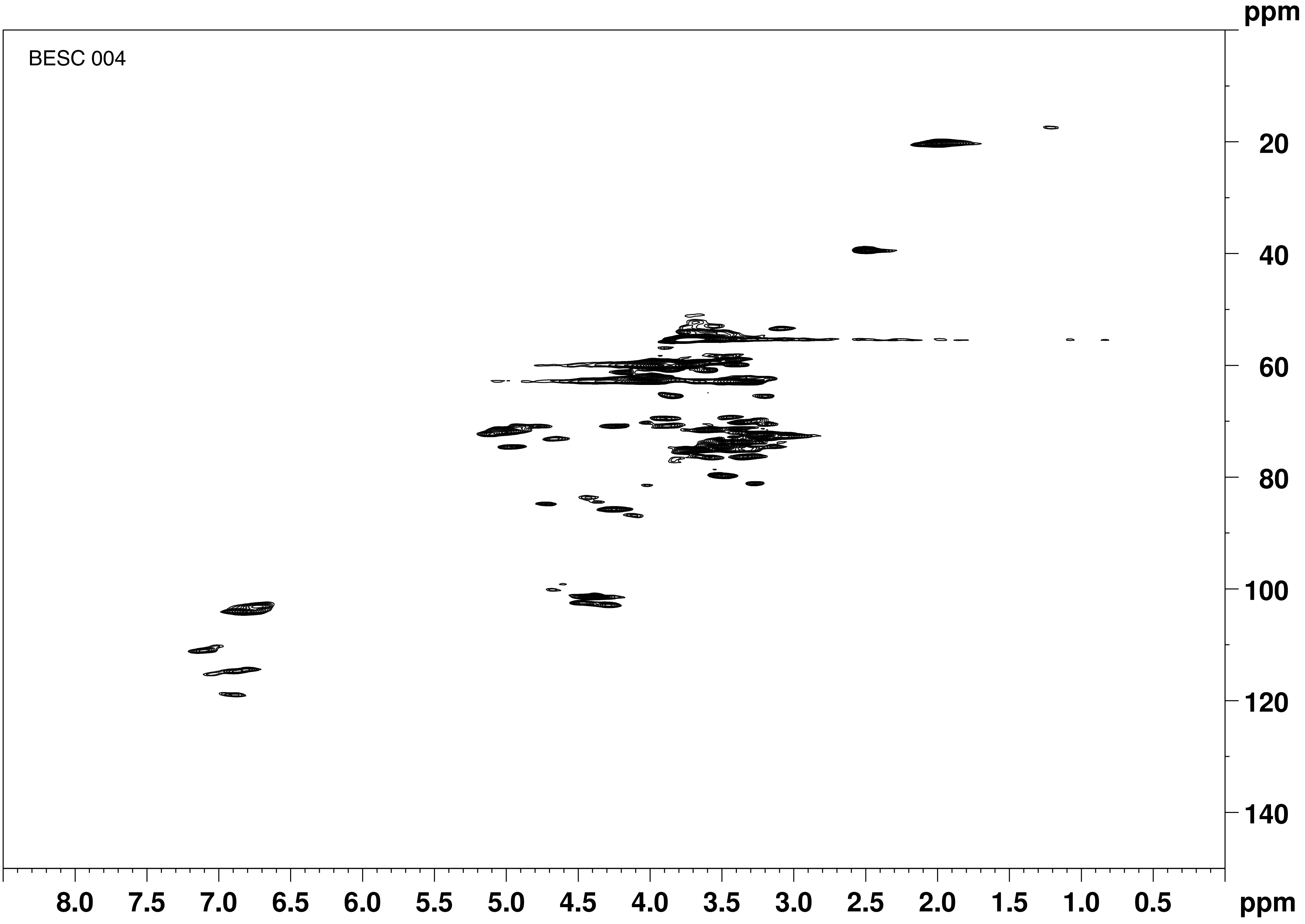
BESC 004
ppm
20
40
60
80
100
120
140
8.0
7.5
7.0
6.5
6.0
5.5
5.0
4.5
4.0
3.5
3.0
2.5
2.0
1.5
1.0
0.5
ppm

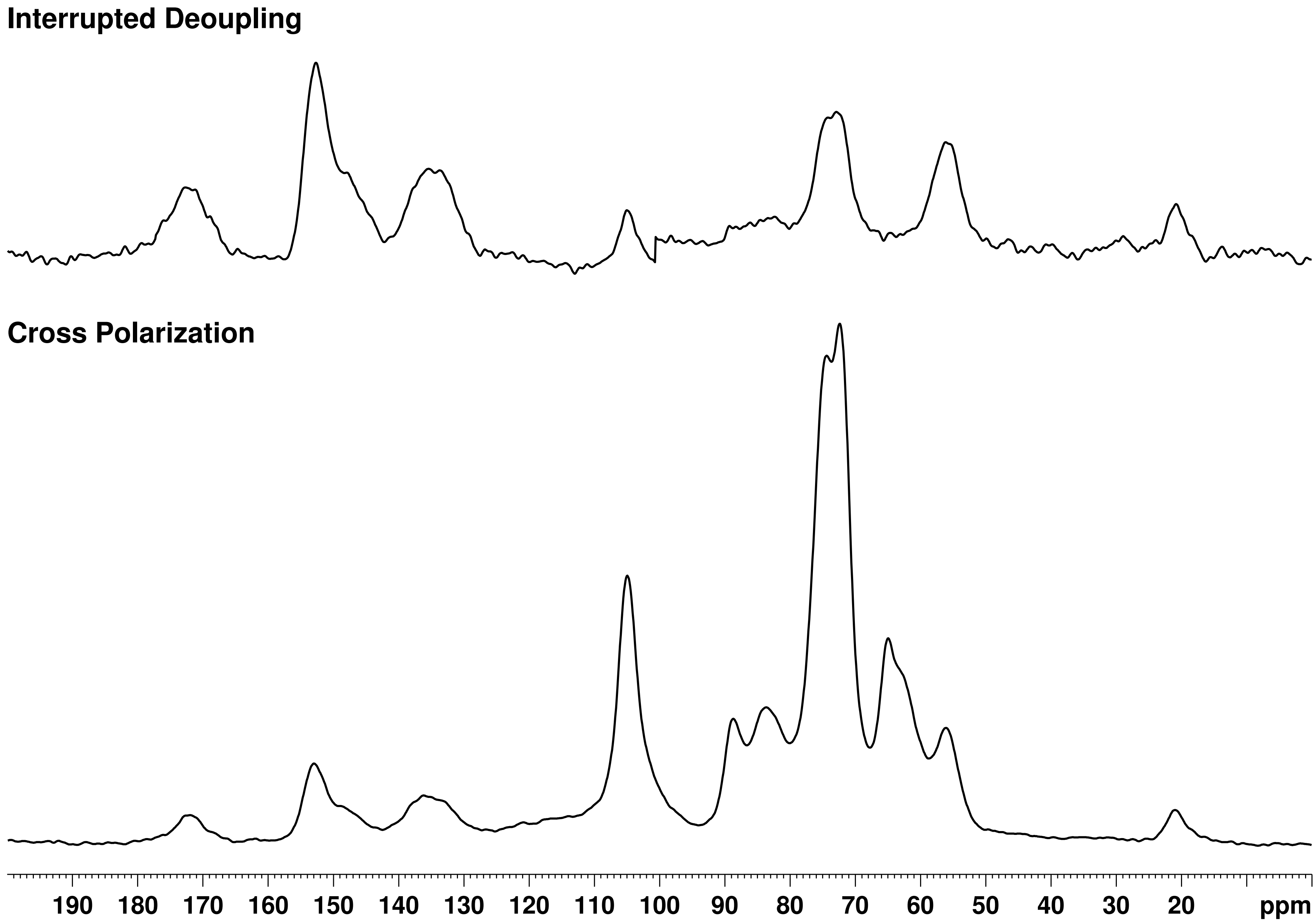
Interrupted Deoupling
Cross Polarization
190
180
170
160
150
140
130
120
110
100
90
80
70
60
50
40
30
20
ppm

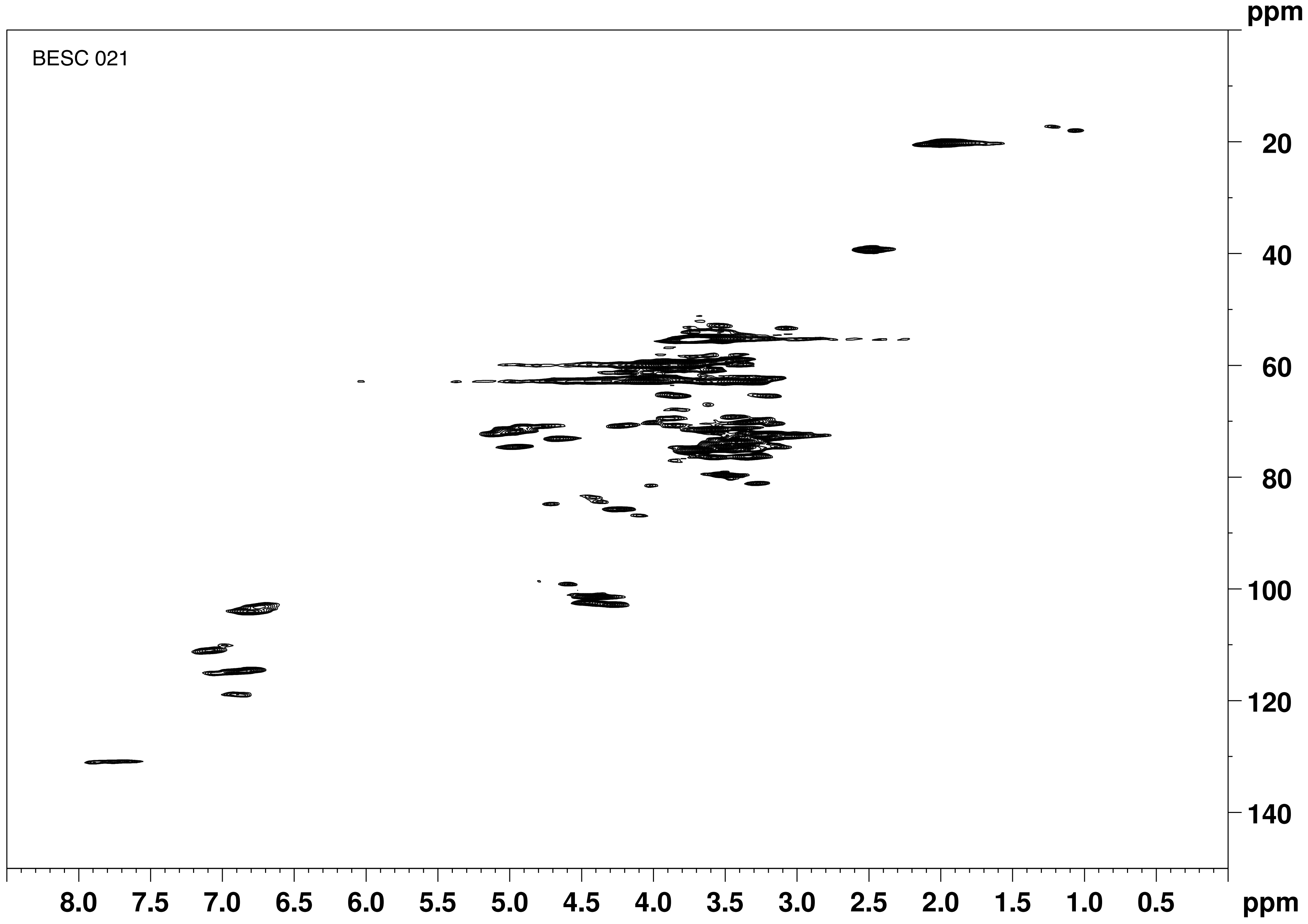
BESC 021
ppm
20
40
60
80
100
120
140
8.0 7.5 7.0 6.5 6.0 5.5 5.0 4.5 4.0 3.5 3.0 2.5 2.0 1.5 1.0 0.5
ppm

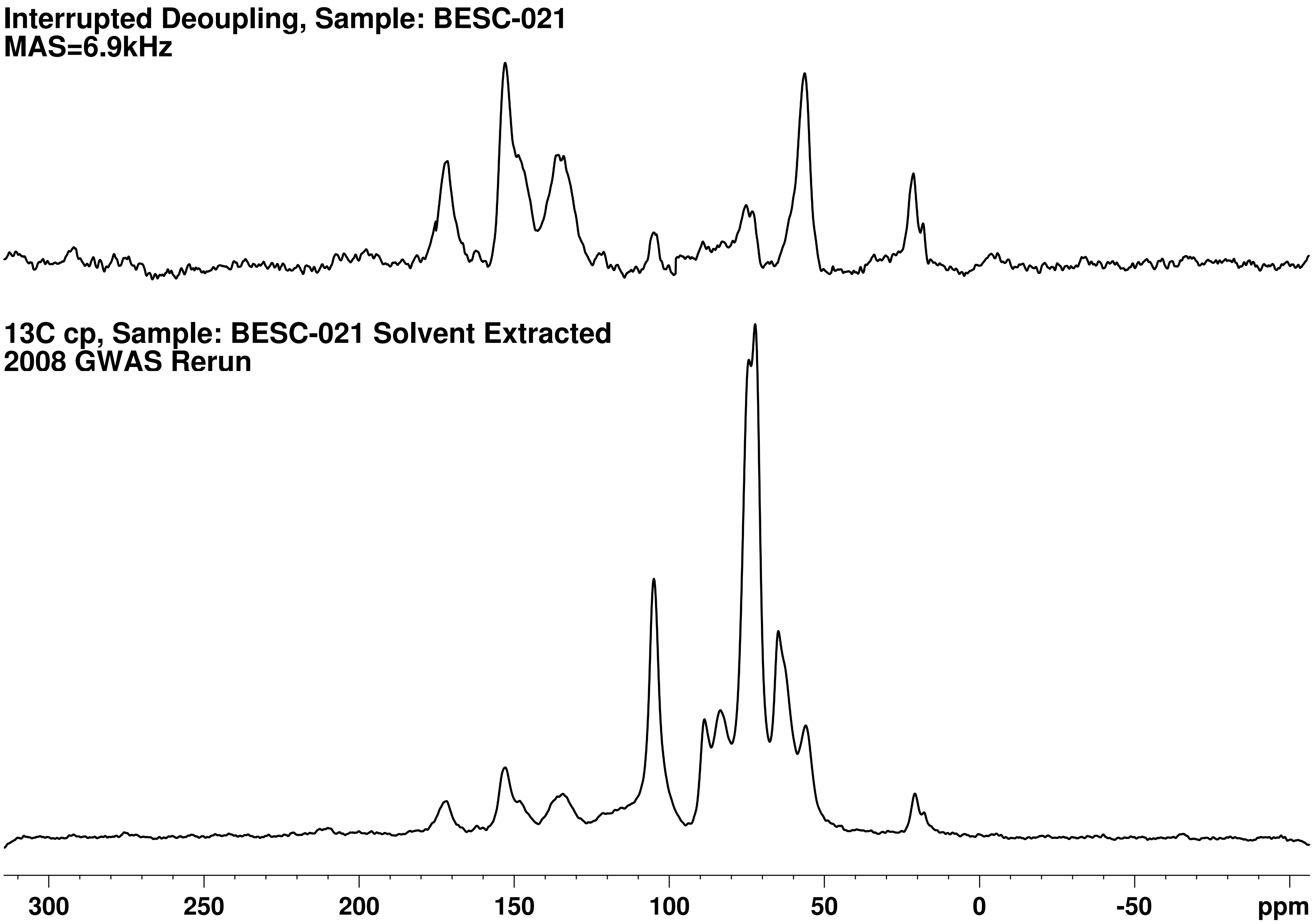

Interrupted Deoupling, Sample: BESC-021
MAS=6.9kHz
13C cp, Sample: BESC-021 Solvent Extracted
2008 GWAS Rerun
300
250
200
150
100
50
0
-50
ppm

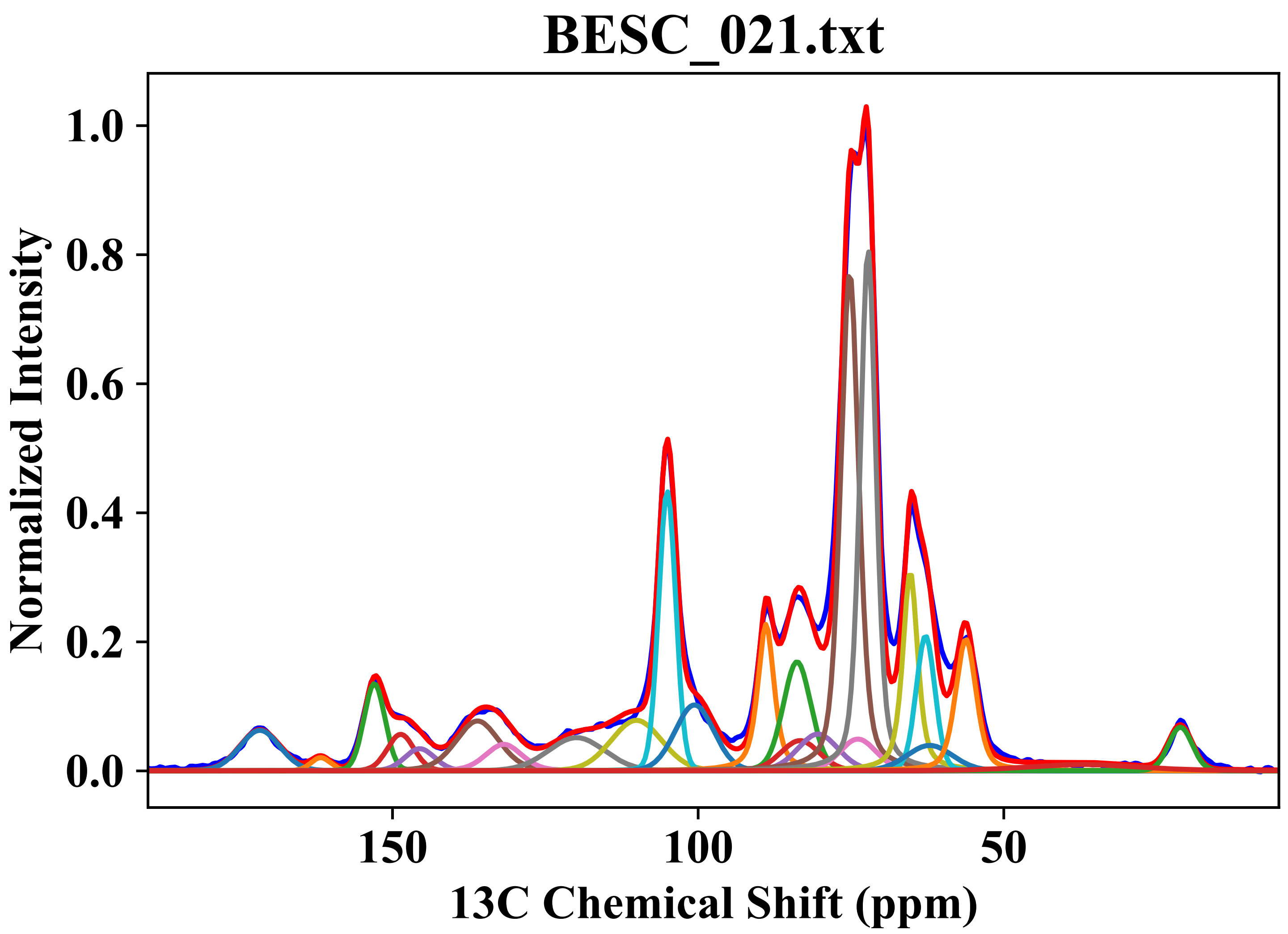

BESC_021.txt
Normalized Intensity
1.0
0.8
0.6
0.4
0.2
0.0
150
100
50
13C Chemical Shift (ppm)

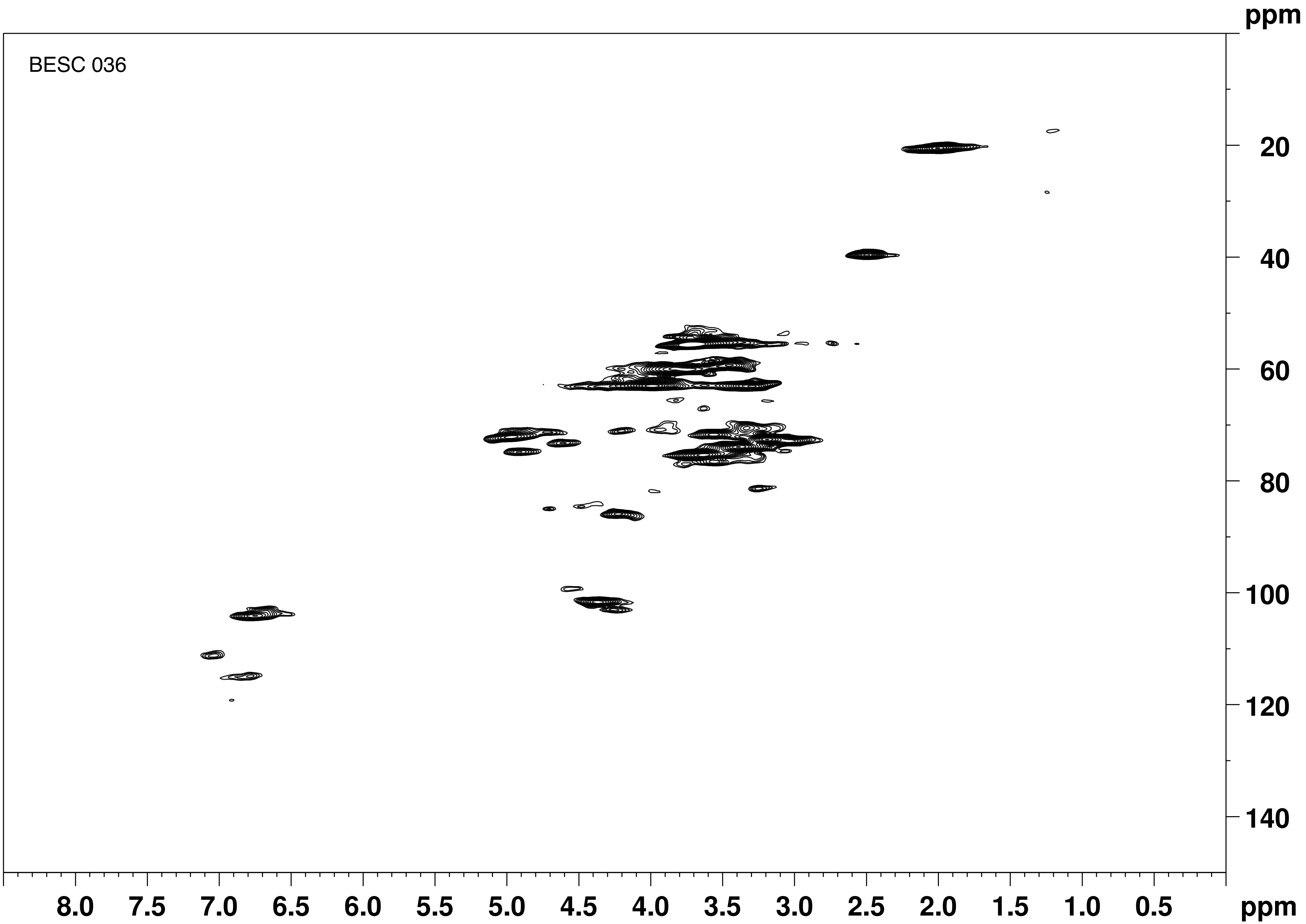

BESC 036
ppm
20
40
60
80
100
120
140
8.0
7.5
7.0
6.5
6.0
5.5
5.0
4.5
4.0
3.5
3.0
2.5
2.0
1.5
1.0
0.5
ppm

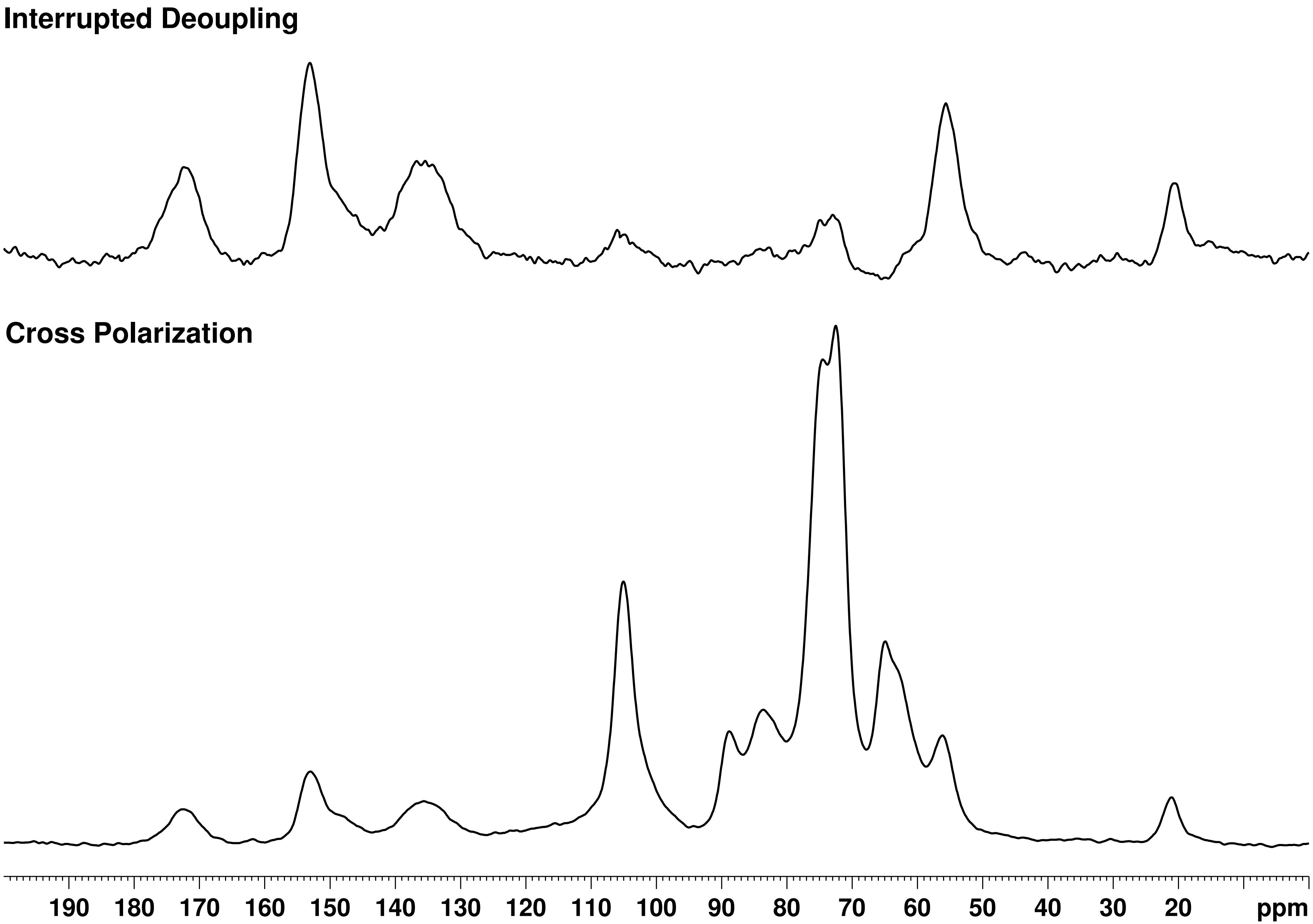
Interrupted Deoupling
Cross Polarization
190 180 170 160 150 140 130 120 110 100 90 80 70 60 50 40 30 20 ppm

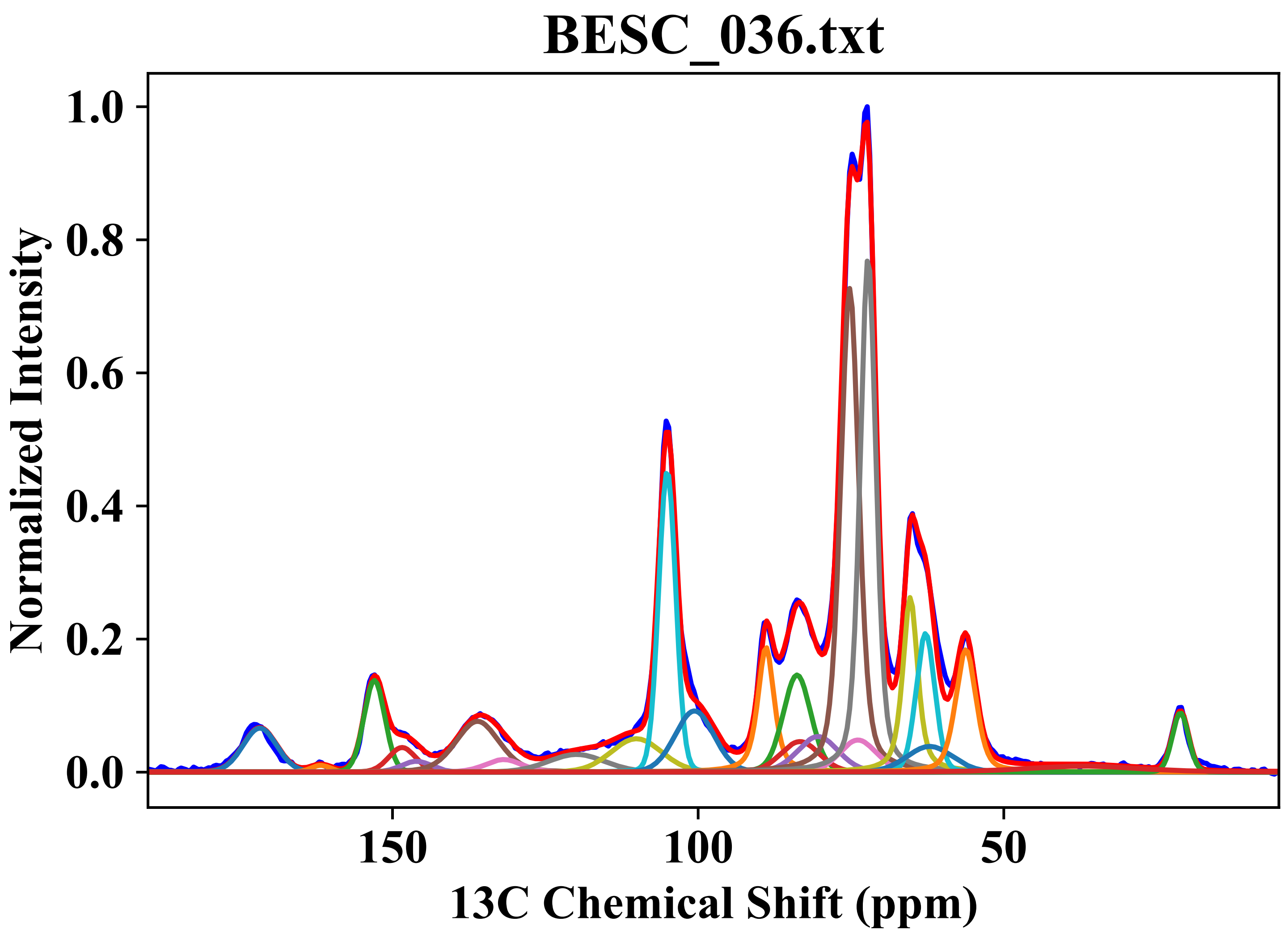
BESC_036.txt
Normalized Intensity
0.0
0.2
0.4
0.6
0.8
1.0
150
100
50
13C Chemical Shift (ppm)

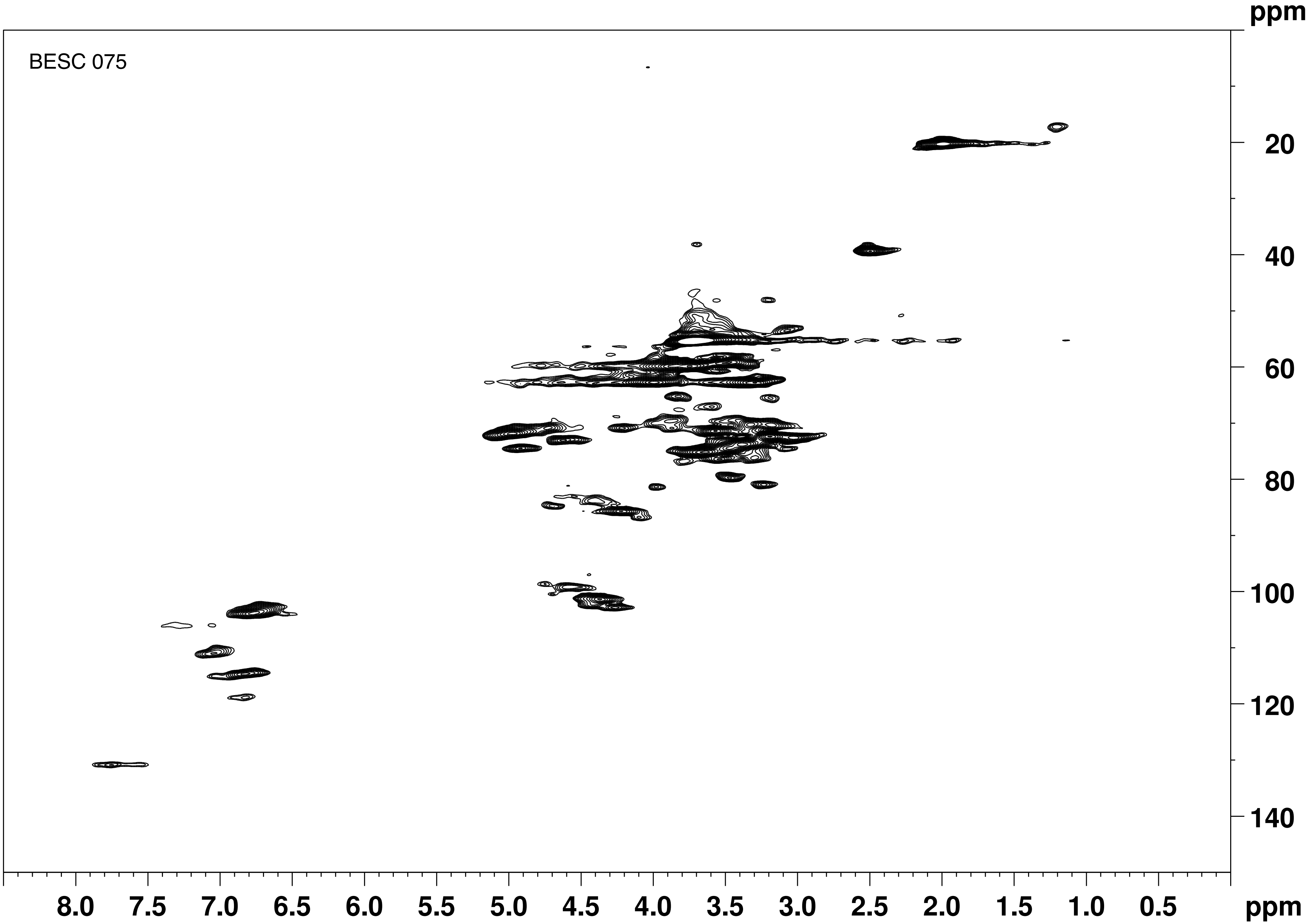
BESC 075
ppm
20
40
60
80
100
120
140
8.0
7.5
7.0
6.5
6.0
5.5
5.0
4.5
4.0
3.5
3.0
2.5
2.0
1.5
1.0
0.5
ppm

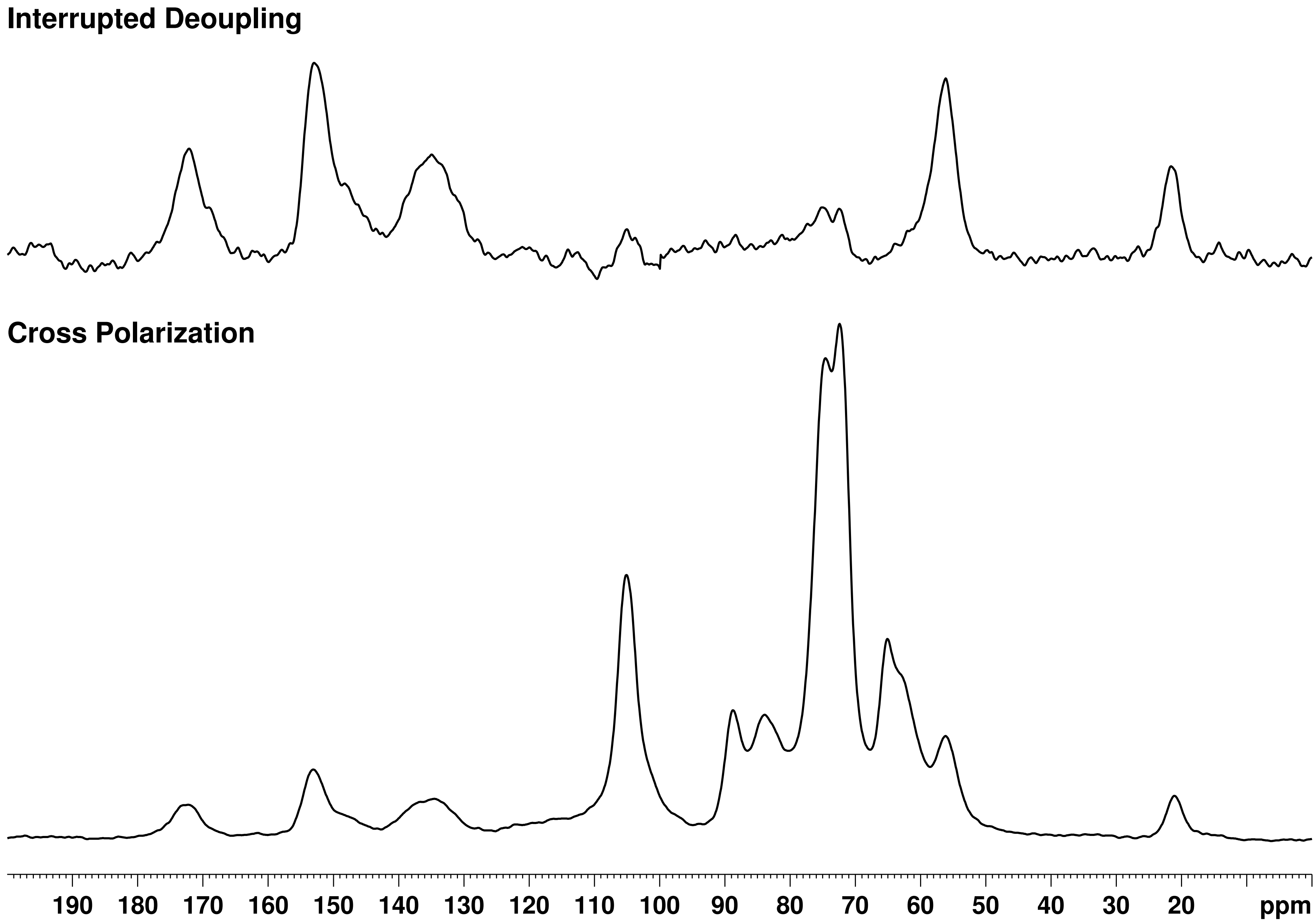
Interrupted Deoupling
Cross Polarization
190
180
170
160
150
140
130
120
110
100
90
80
70
60
50
40
30
20
ppm

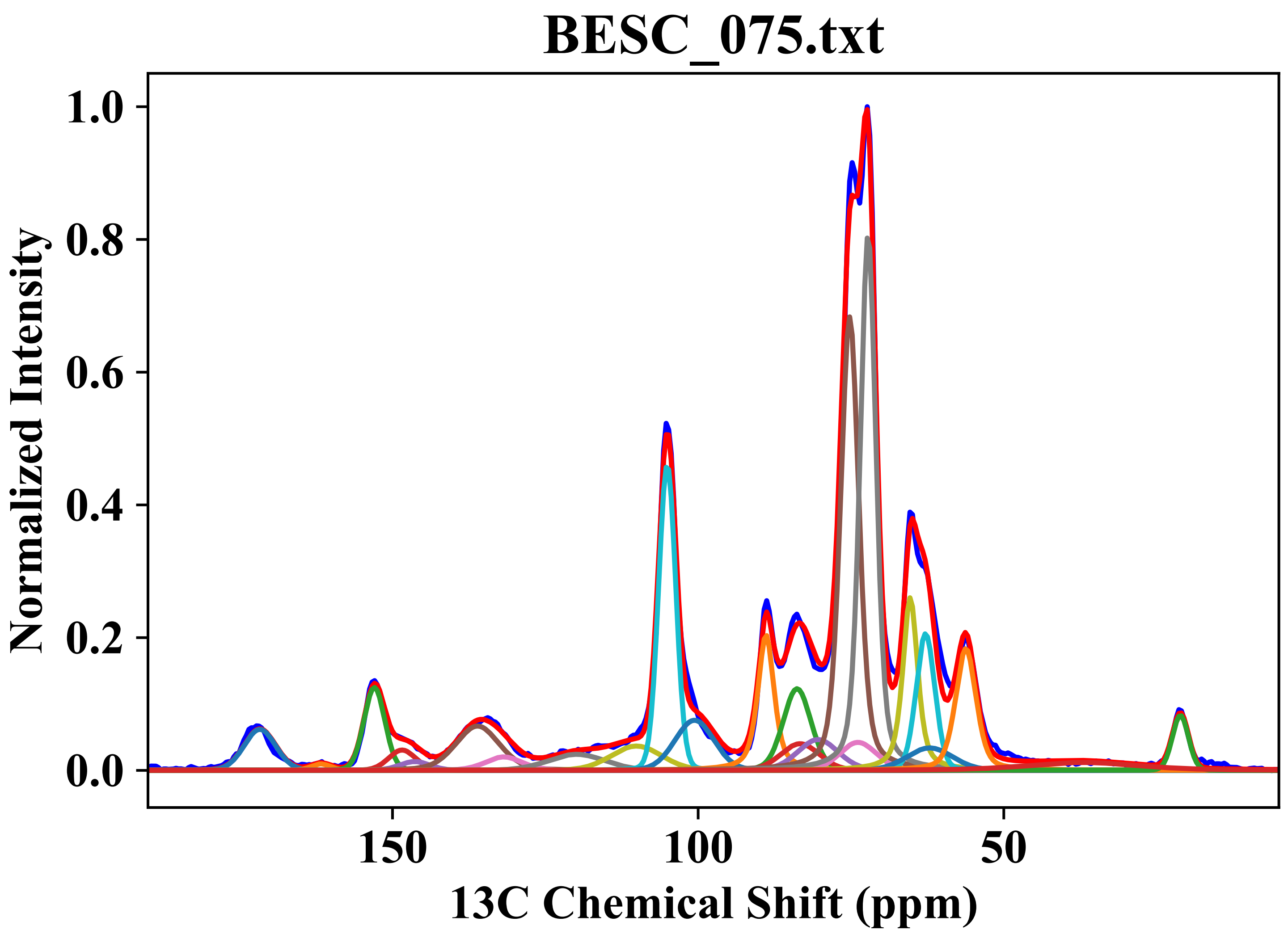
BESC_075.txt
Normalized Intensity
0.0
0.2
0.4
0.6
0.8
1.0
150
100
50
13C Chemical Shift (ppm)

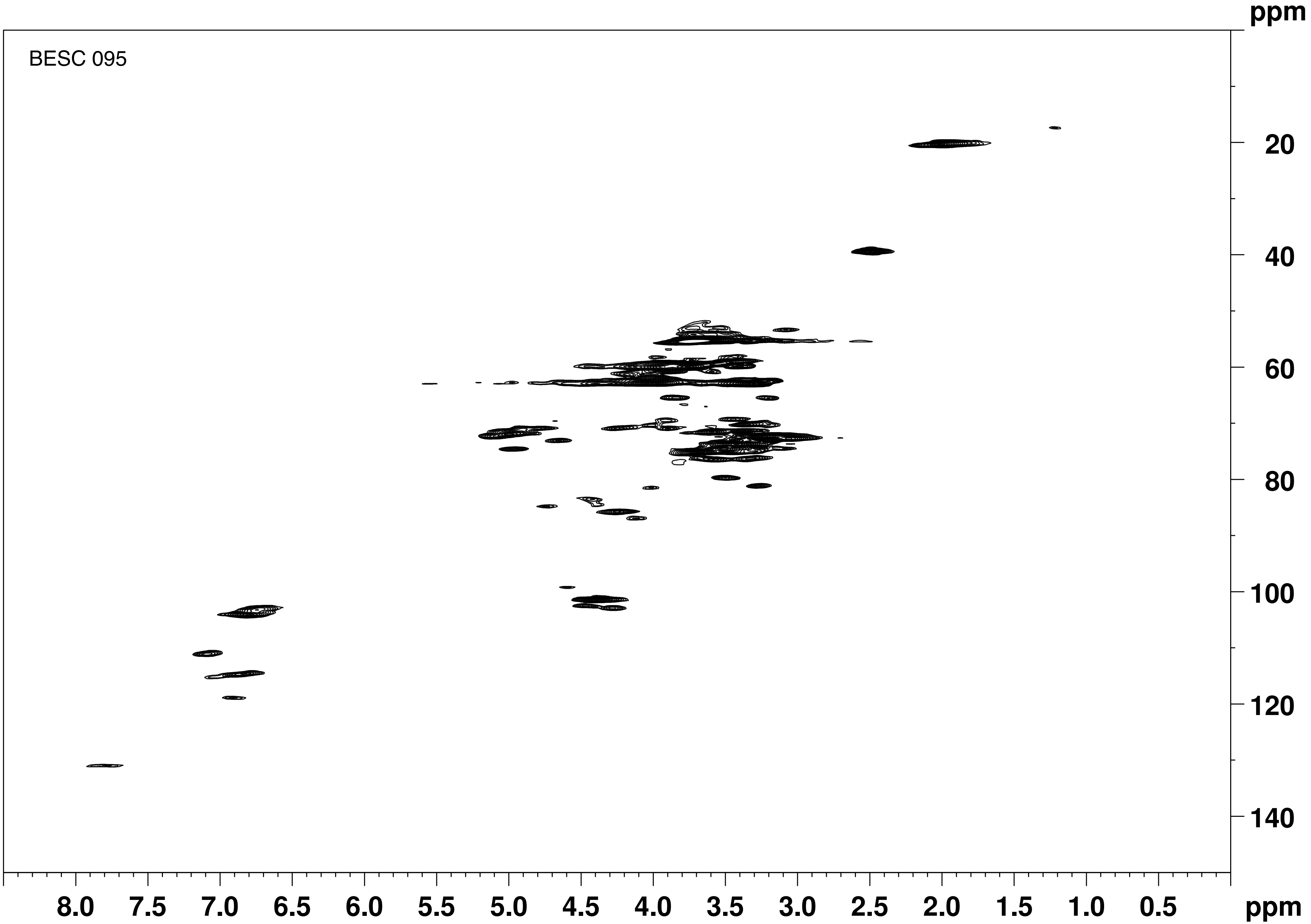
BESC 095
ppm
20
40
60
80
100
120
140
8.0 7.5 7.0 6.5 6.0 5.5 5.0 4.5 4.0 3.5 3.0 2.5 2.0 1.5 1.0 0.5
ppm

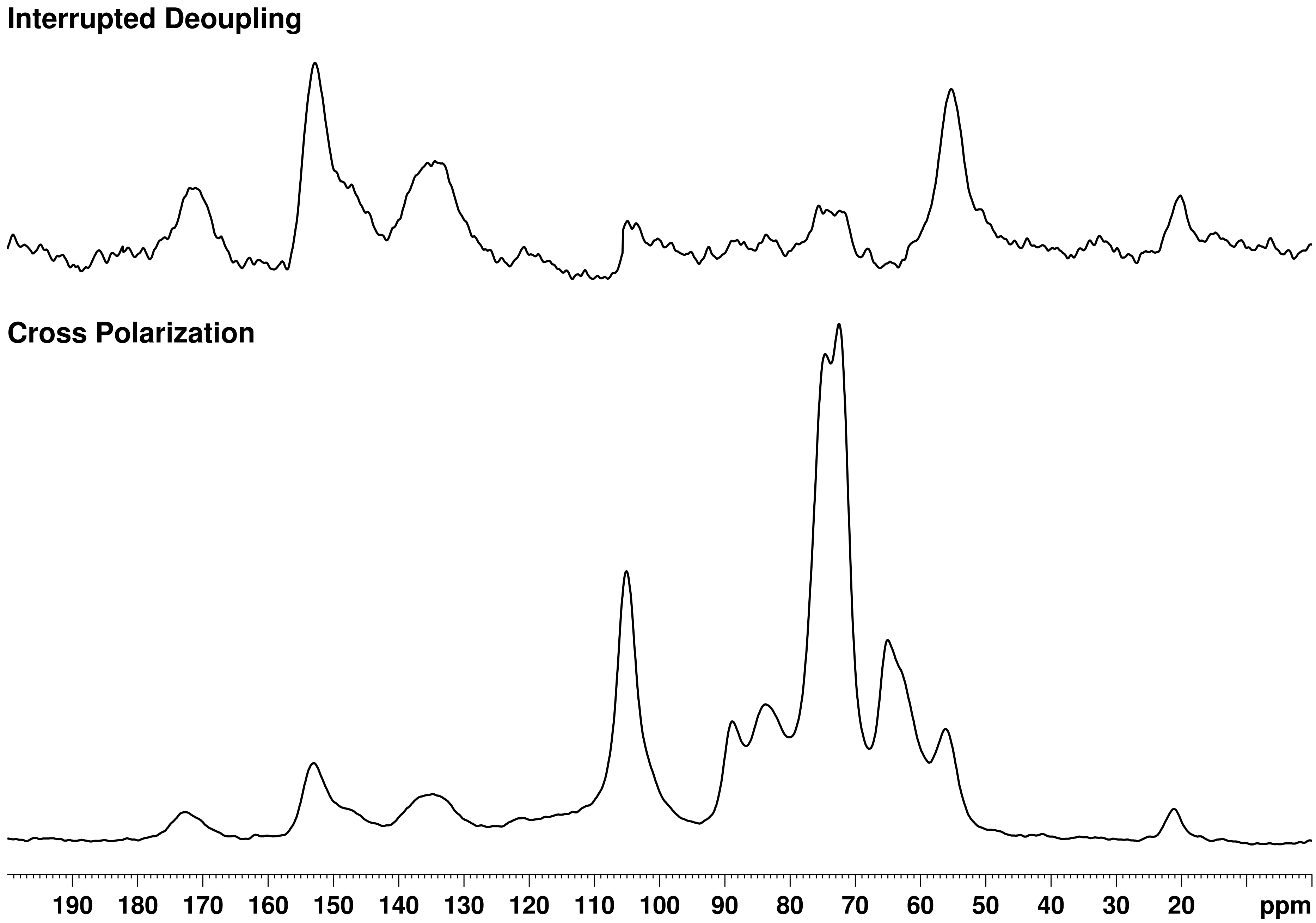

Interrupted Deoupling
Cross Polarization
190 180 170 160 150 140 130 120 110 100 90 80 70 60 50 40 30 20 ppm

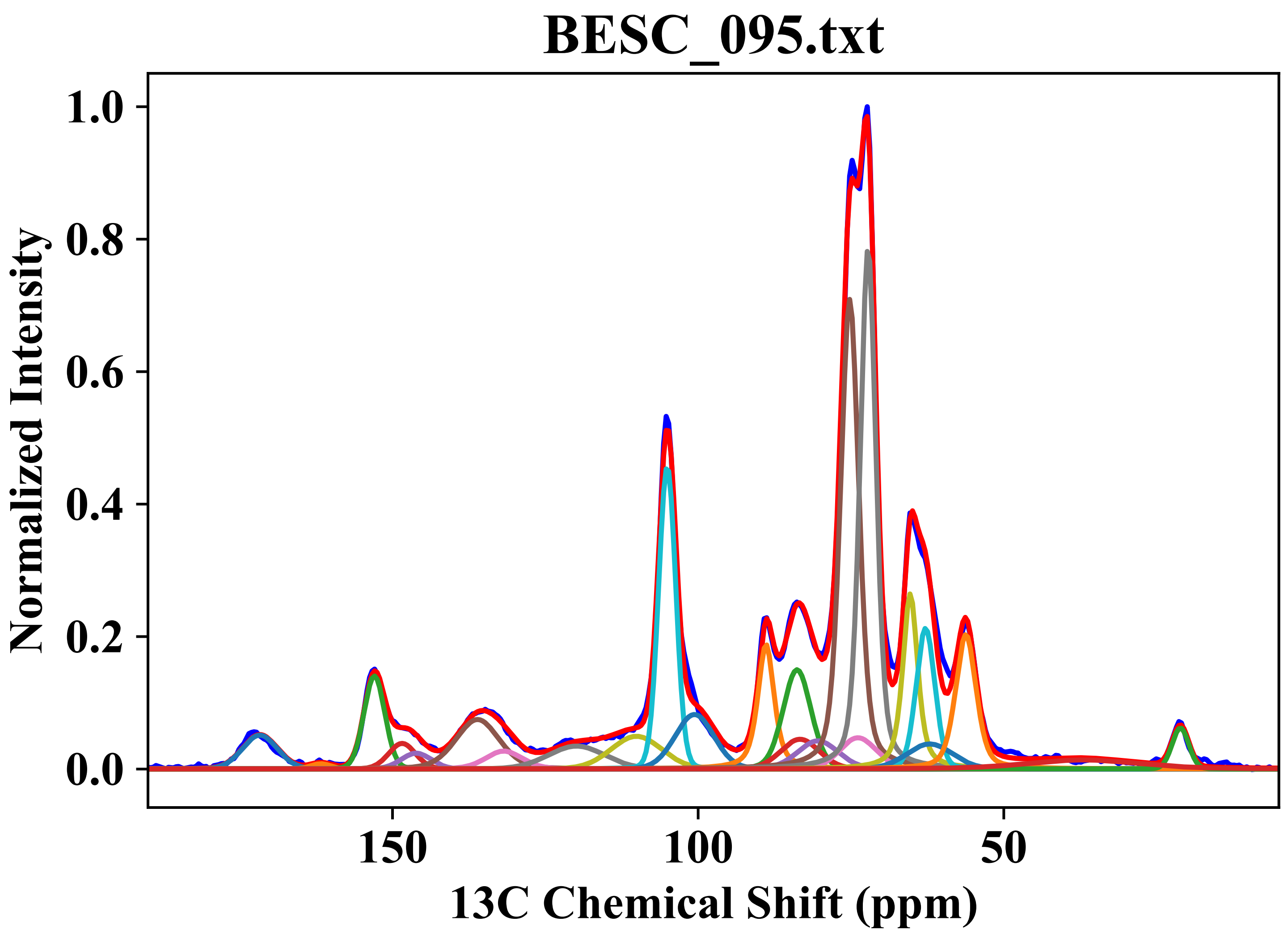
BESC_095.txt
Normalized Intensity
1.0
0.8
0.6
0.4
0.2
0.0
150
100
50
13C Chemical Shift (ppm)

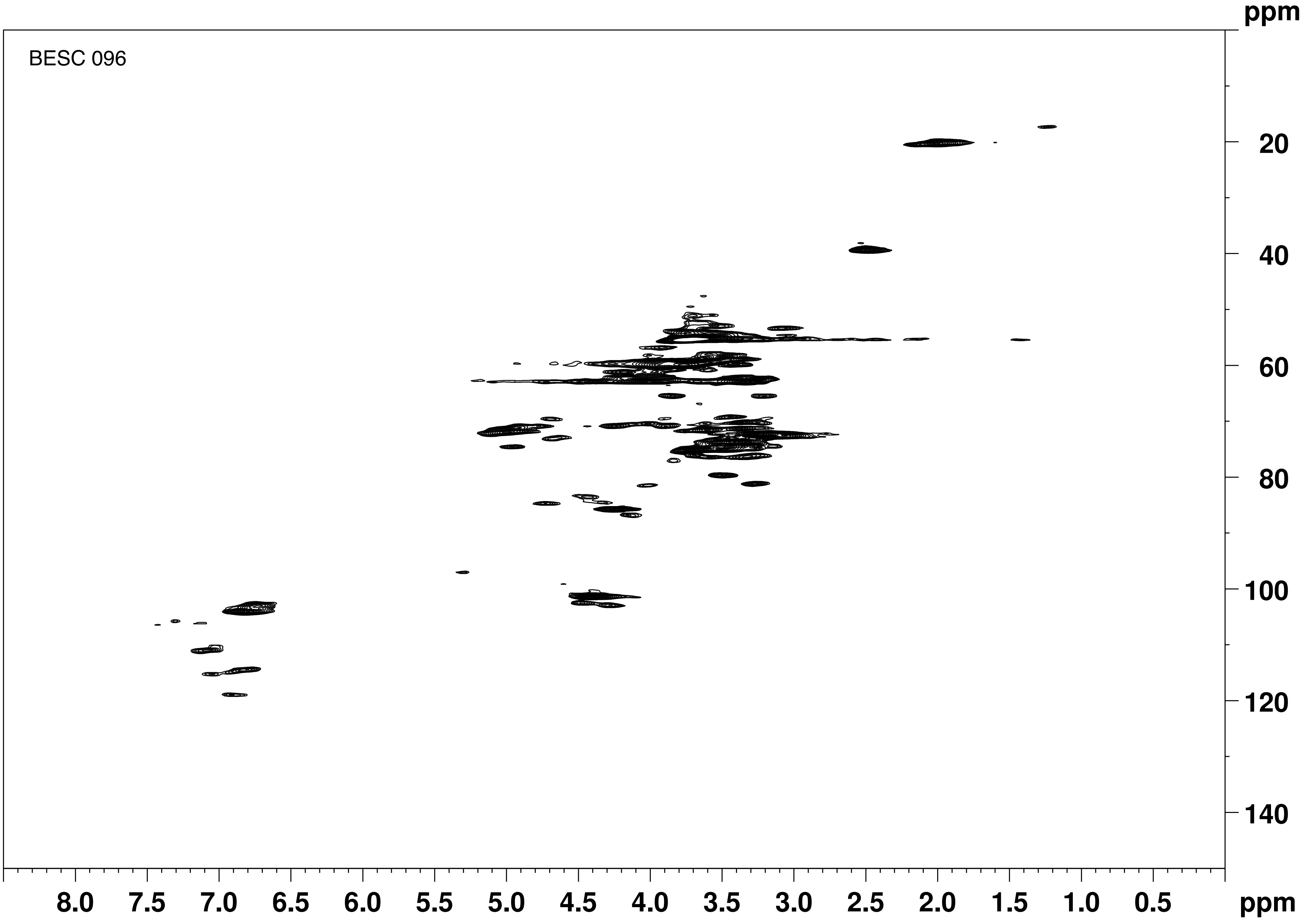
BESC 096
ppm
20
40
60
80
100
120
140
8.0 7.5 7.0 6.5 6.0 5.5 5.0 4.5 4.0 3.5 3.0 2.5 2.0 1.5 1.0 0.5
ppm

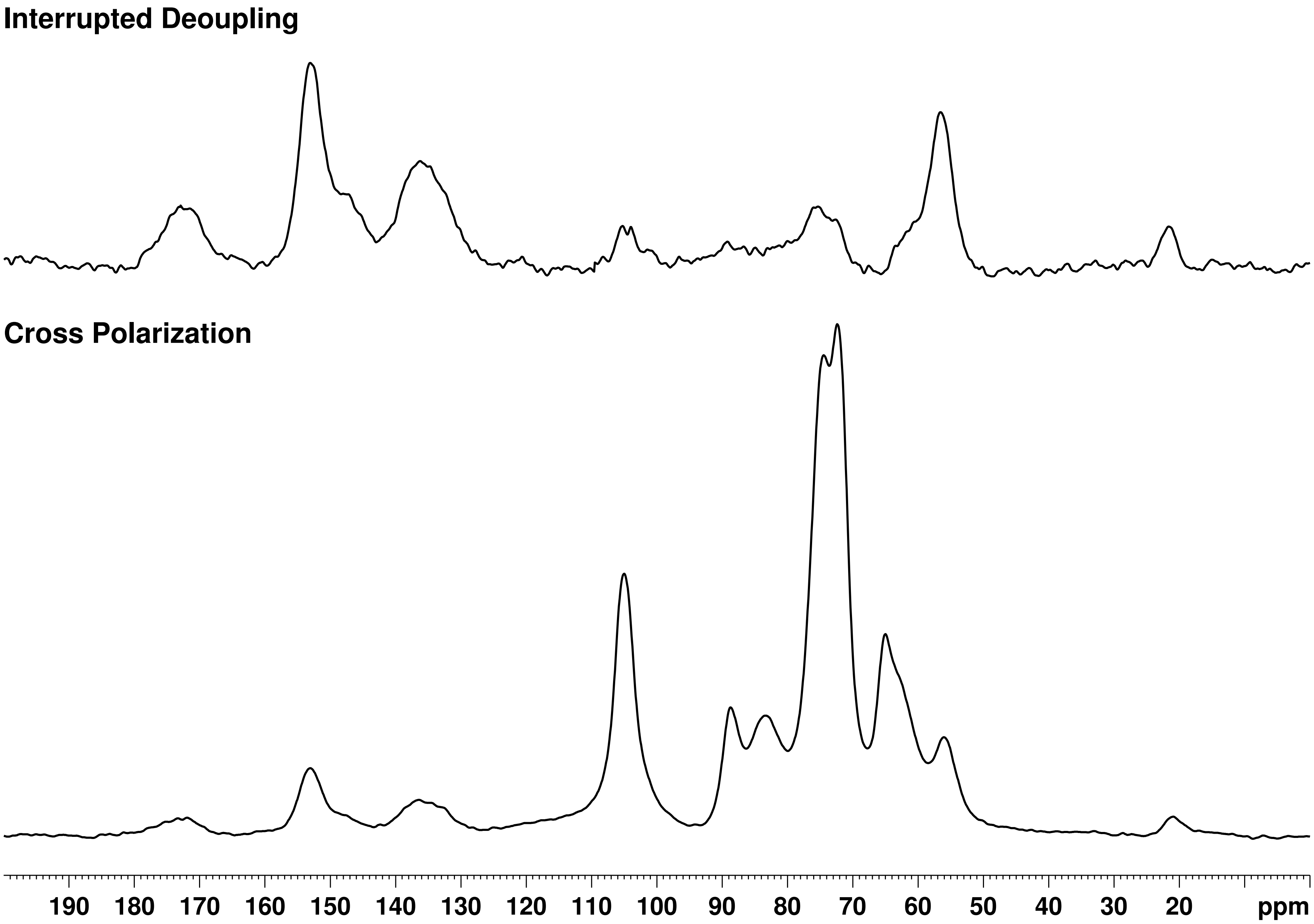
Interrupted Deoupling
Cross Polarization
190 180 170 160 150 140 130 120 110 100 90 80 70 60 50 40 30 20 ppm

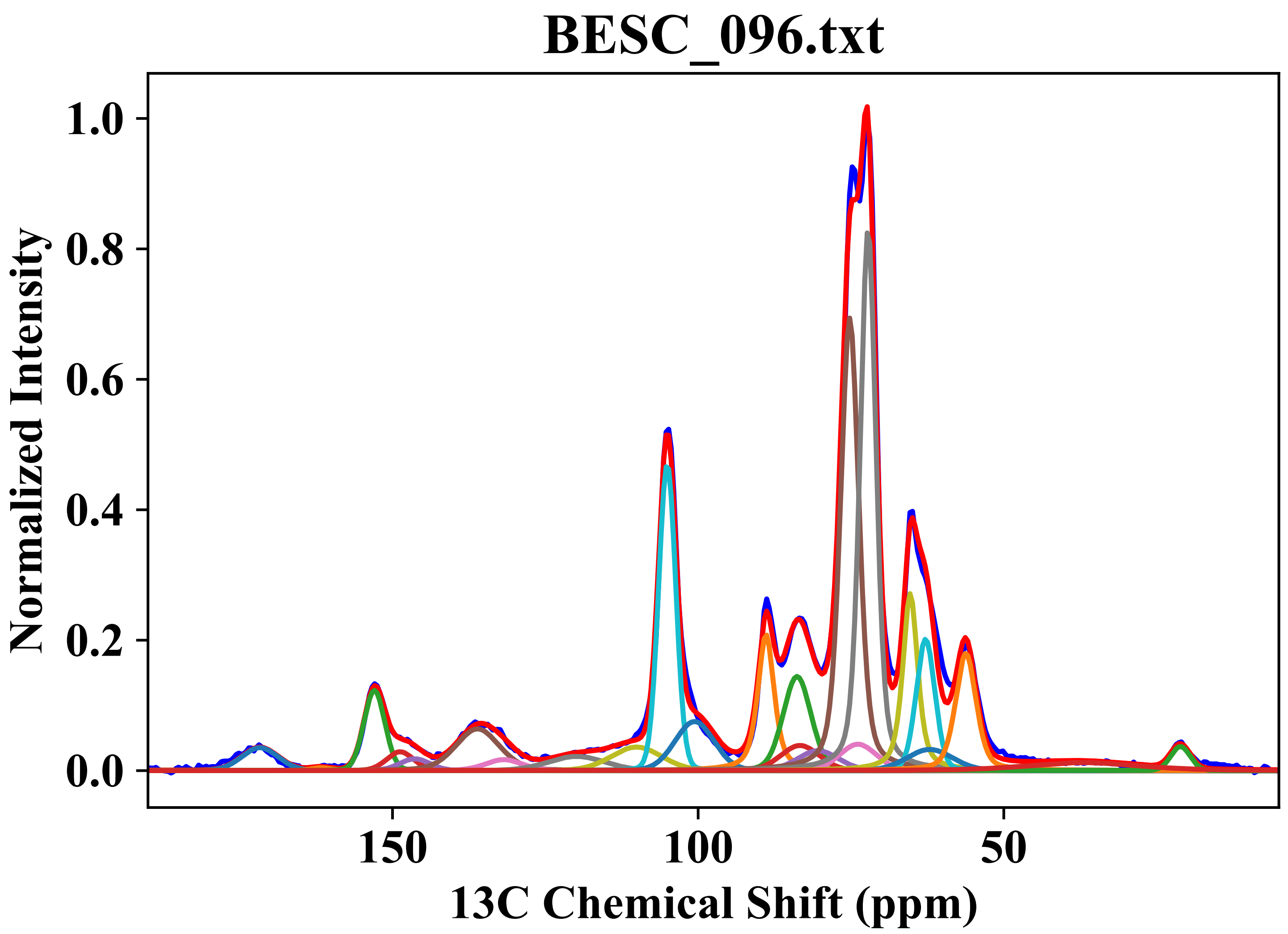
BESC_096.txt
Normalized Intensity
1.0
0.8
0.6
0.4
0.2
0.0
150
100
50
13C Chemical Shift (ppm)

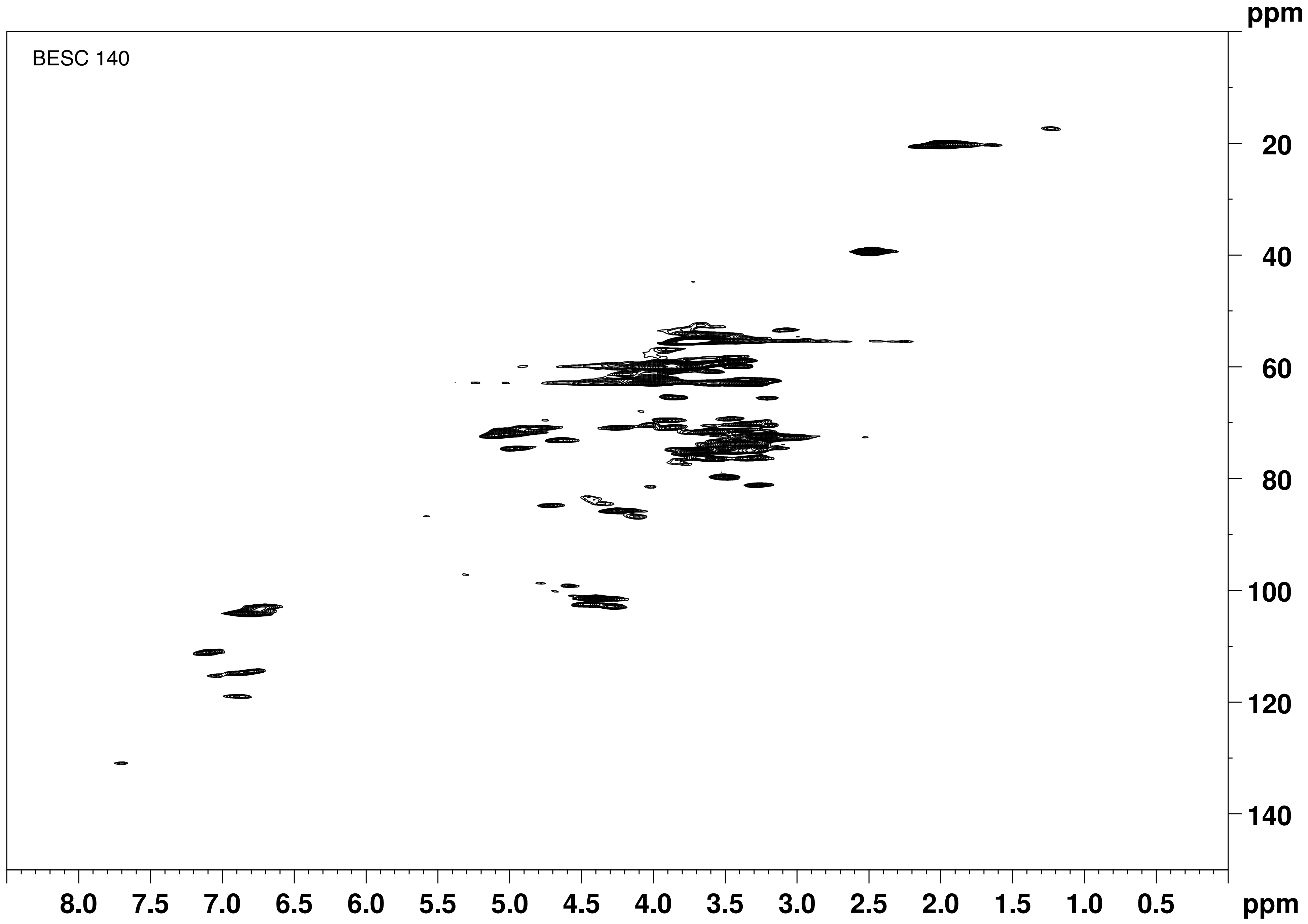

BESC 140
ppm
20
40
60
80
100
120
140
8.0
7.5
7.0
6.5
6.0
5.5
5.0
4.5
4.0
3.5
3.0
2.5
2.0
1.5
1.0
0.5
ppm

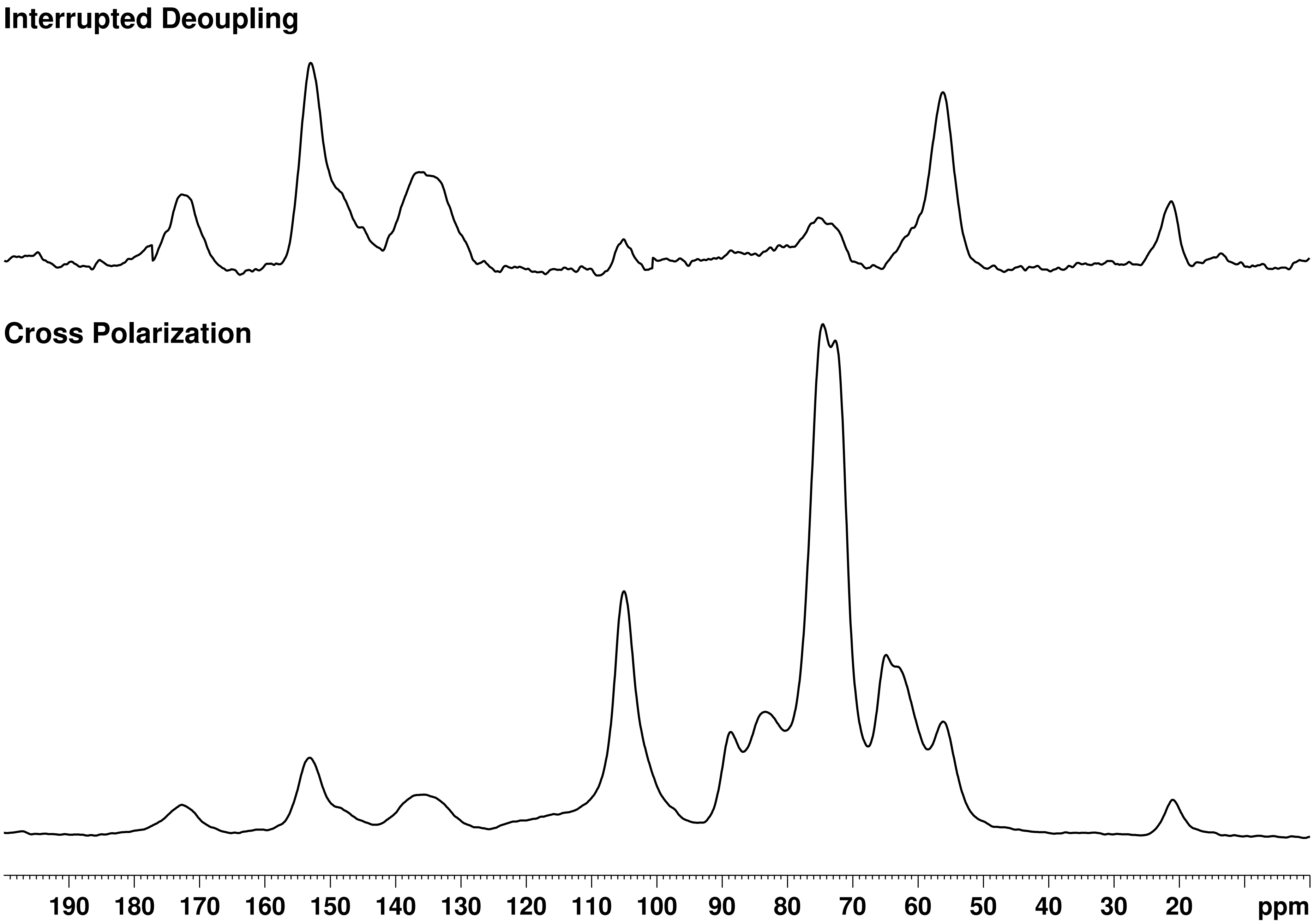
Interrupted Deoupling
Cross Polarization
190 180 170 160 150 140 130 120 110 100 90 80 70 60 50 40 30 20 ppm

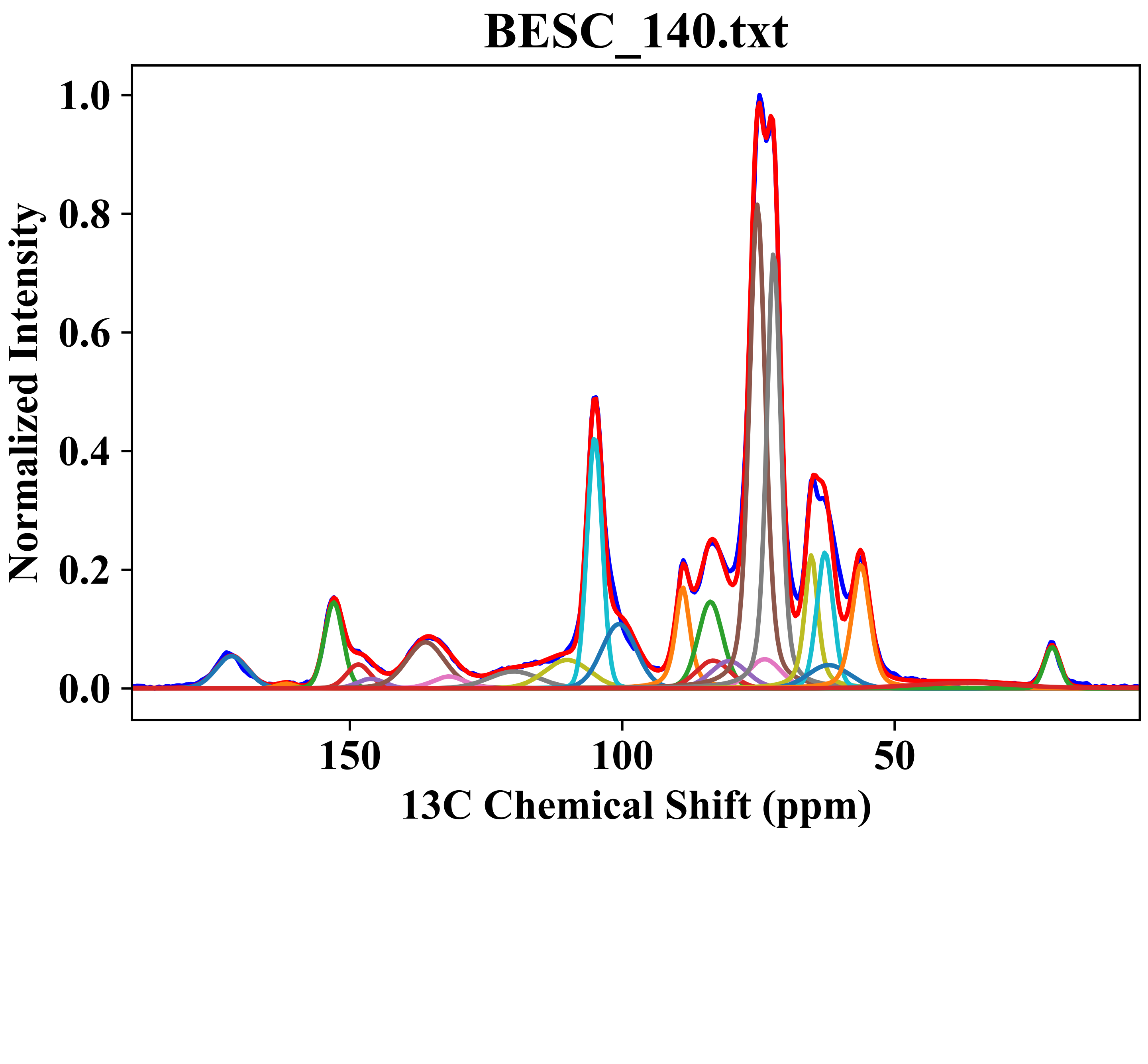
BESC_140.txt
Normalized Intensity
1.0
0.8
0.6
0.4
0.2
0.0
150
100
50
13C Chemical Shift (ppm)

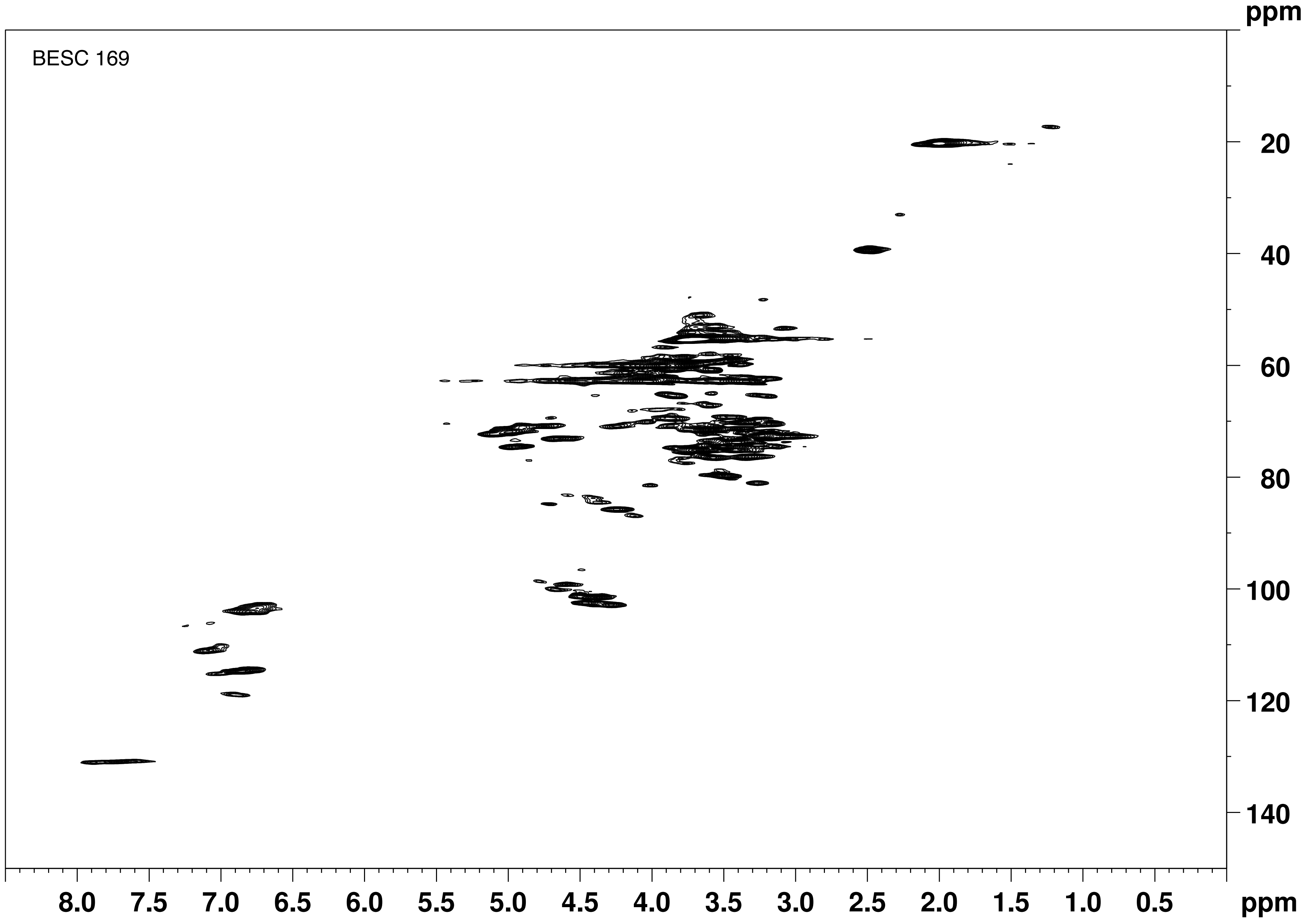
BESC 169
ppm
20
40
60
80
100
120
140
8.0 7.5 7.0 6.5 6.0 5.5 5.0 4.5 4.0 3.5 3.0 2.5 2.0 1.5 1.0 0.5
ppm

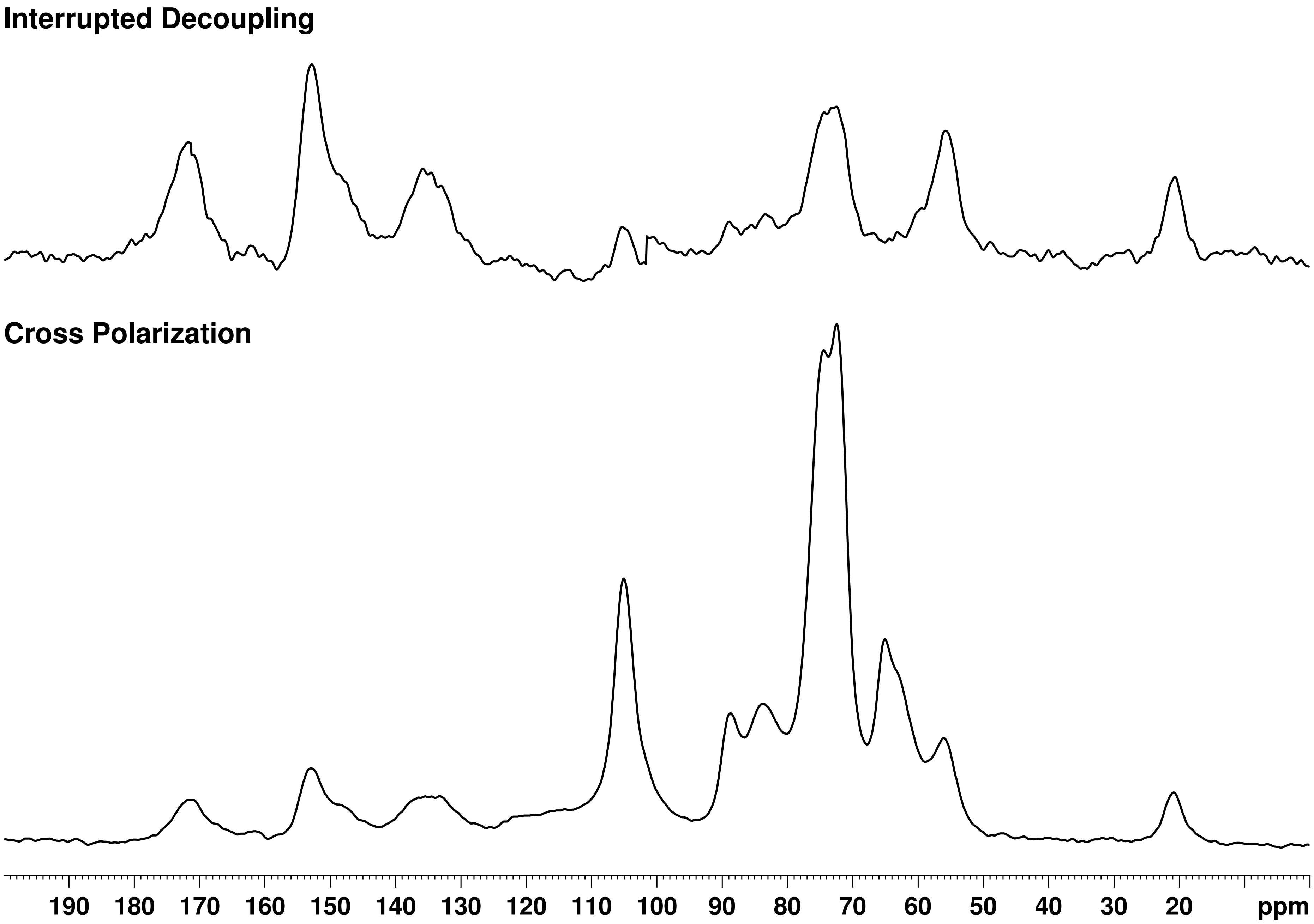
Interrupted Decoupling
Cross Polarization
190 180 170 160 150 140 130 120 110 100 90 80 70 60 50 40 30 20
ppm

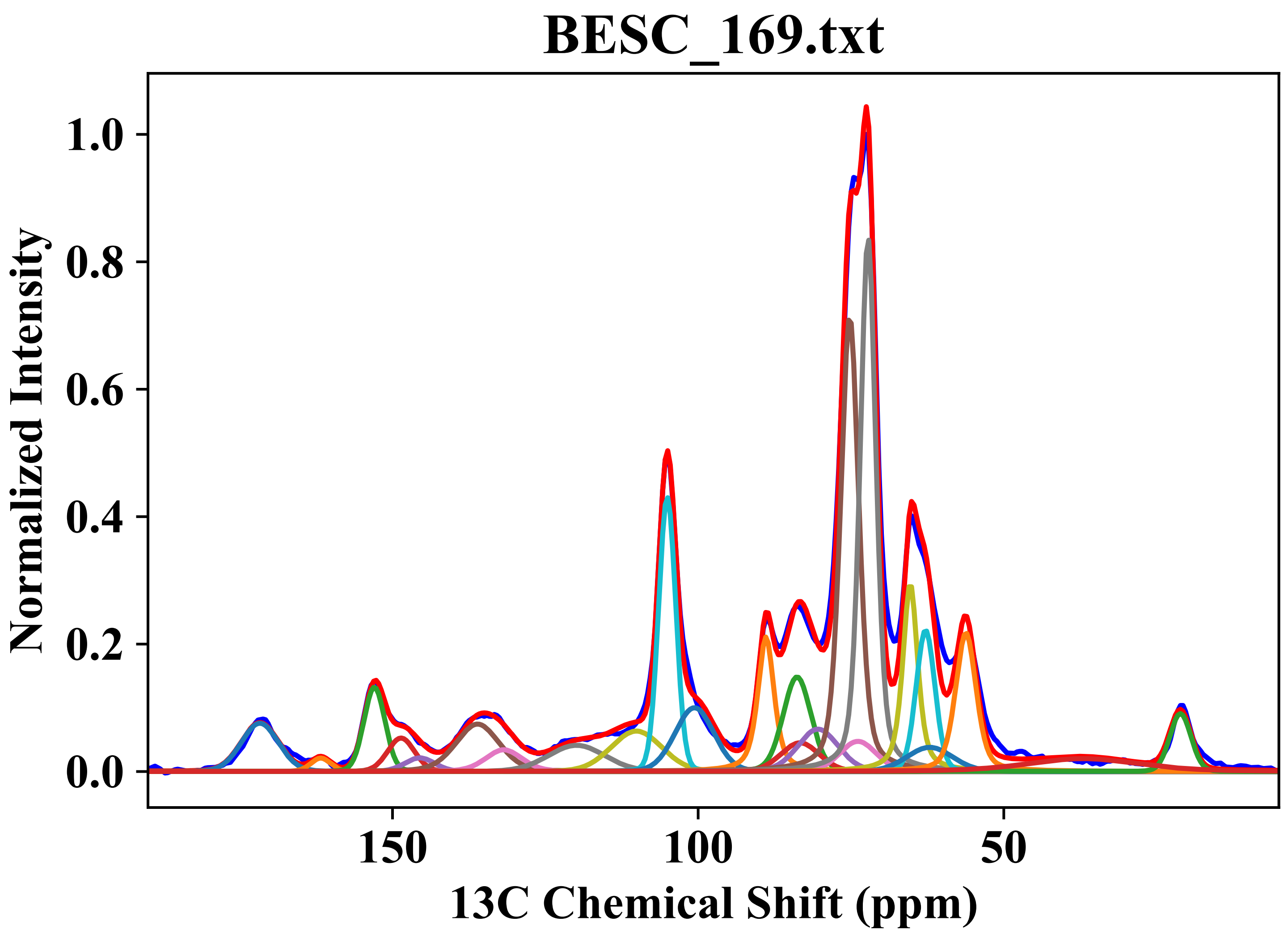
BESC_169.txt
Normalized Intensity
1.0
0.8
0.6
0.4
0.2
0.0
150
100
50
13C Chemical Shift (ppm)

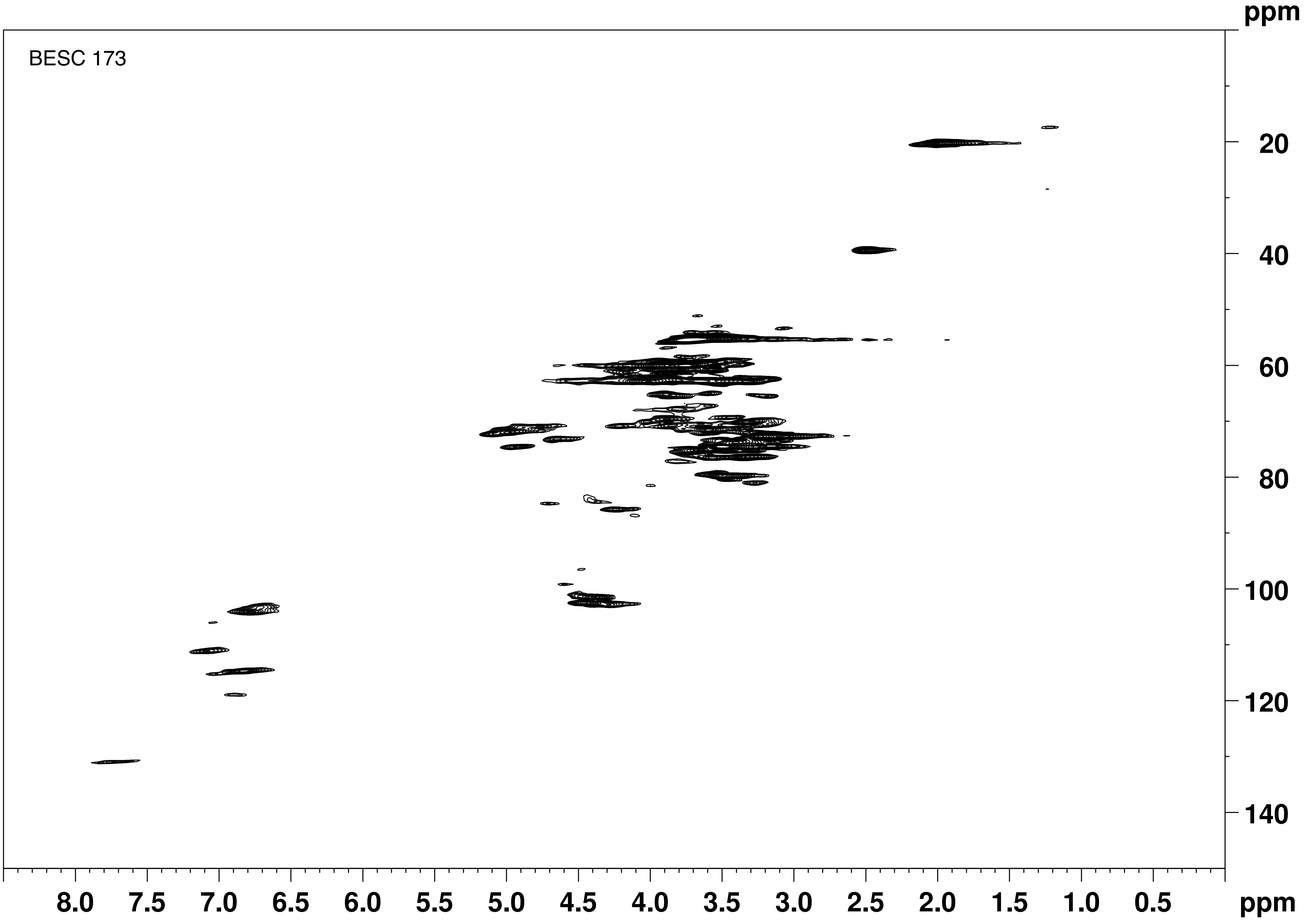
BESC 173
ppm
20
40
60
80
100
120
140
8.0 7.5 7.0 6.5 6.0 5.5 5.0 4.5 4.0 3.5 3.0 2.5 2.0 1.5 1.0 0.5
ppm

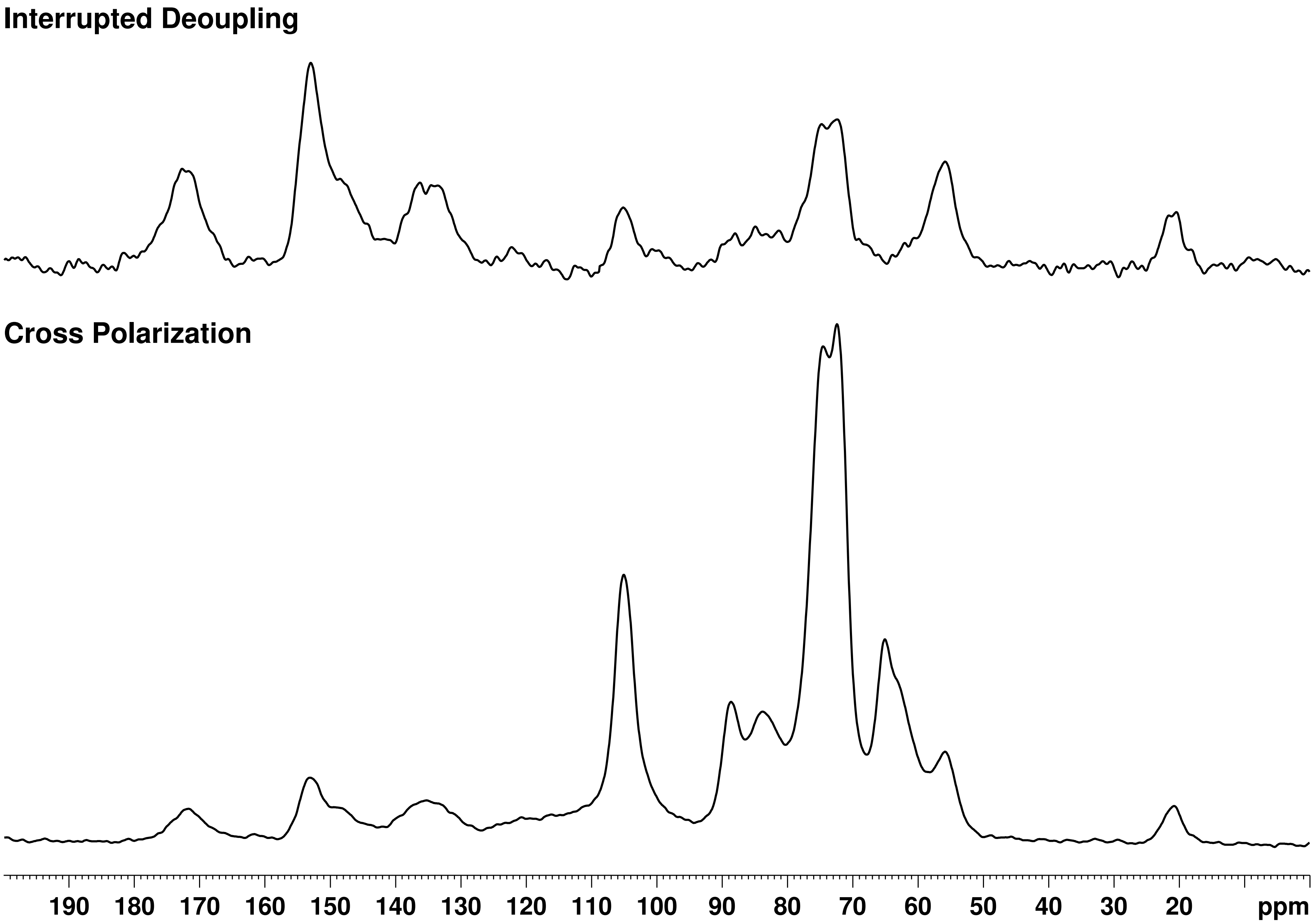
Interrupted Deoupling
Cross Polarization
190 180 170 160 150 140 130 120 110 100 90 80 70 60 50 40 30 20 ppm

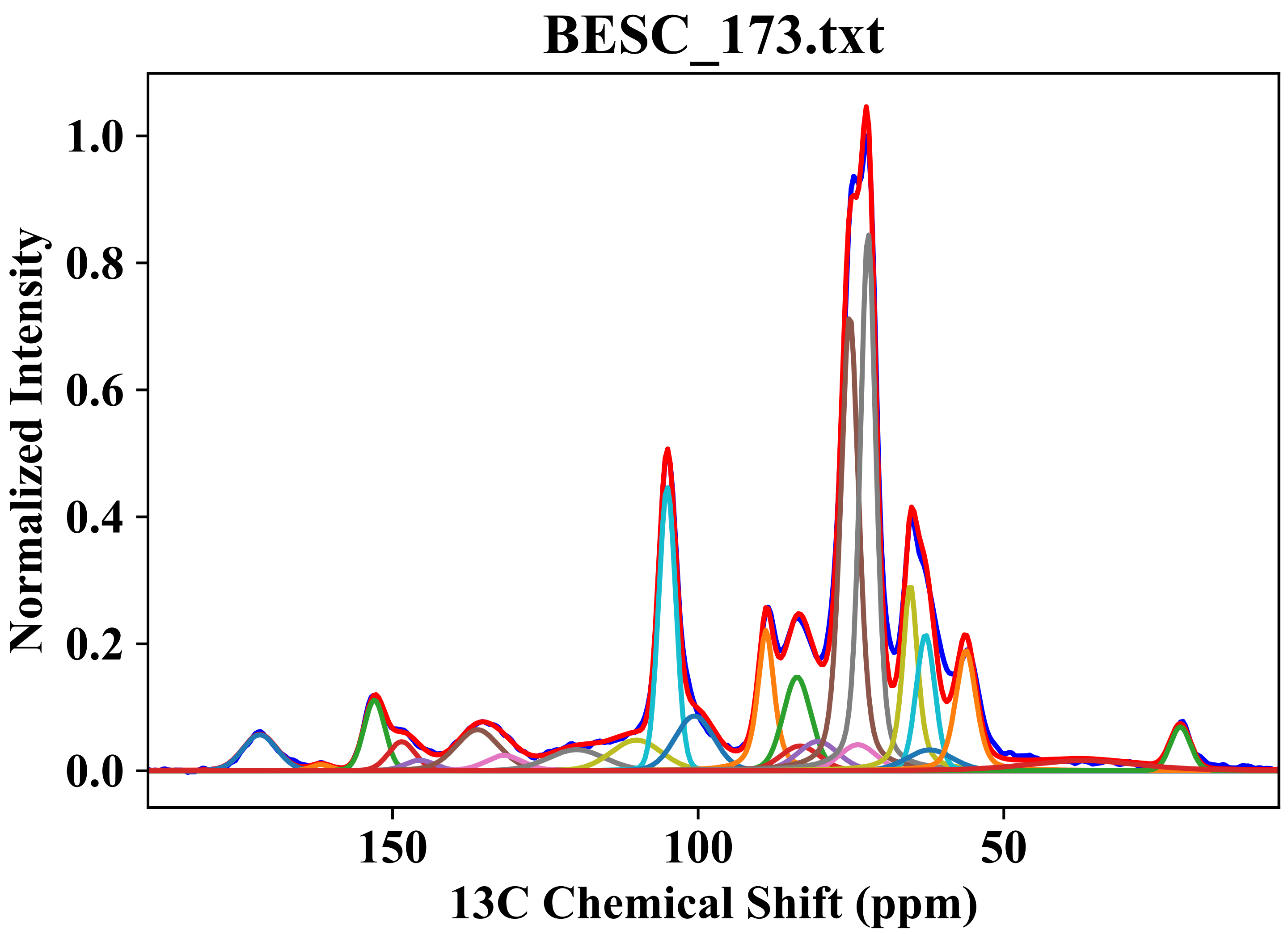

BESC_173.txt
Normalized Intensity
0.0
0.2
0.4
0.6
0.8
1.0
150
100
50
13C Chemical Shift (ppm)

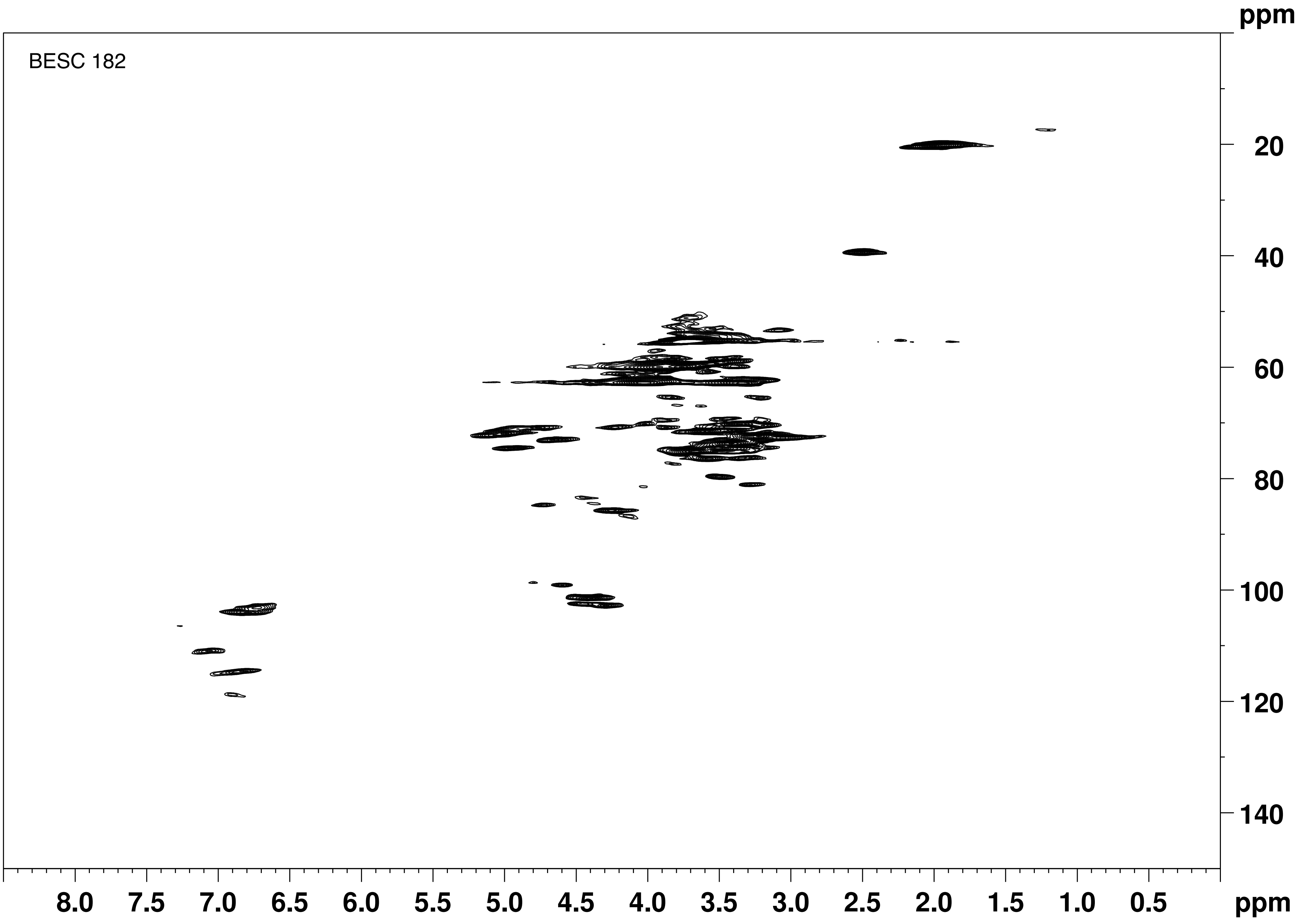
BESC 182
ppm
20
40
60
80
100
120
140
8.0
7.5
7.0
6.5
6.0
5.5
5.0
4.5
4.0
3.5
3.0
2.5
2.0
1.5
1.0
0.5
ppm

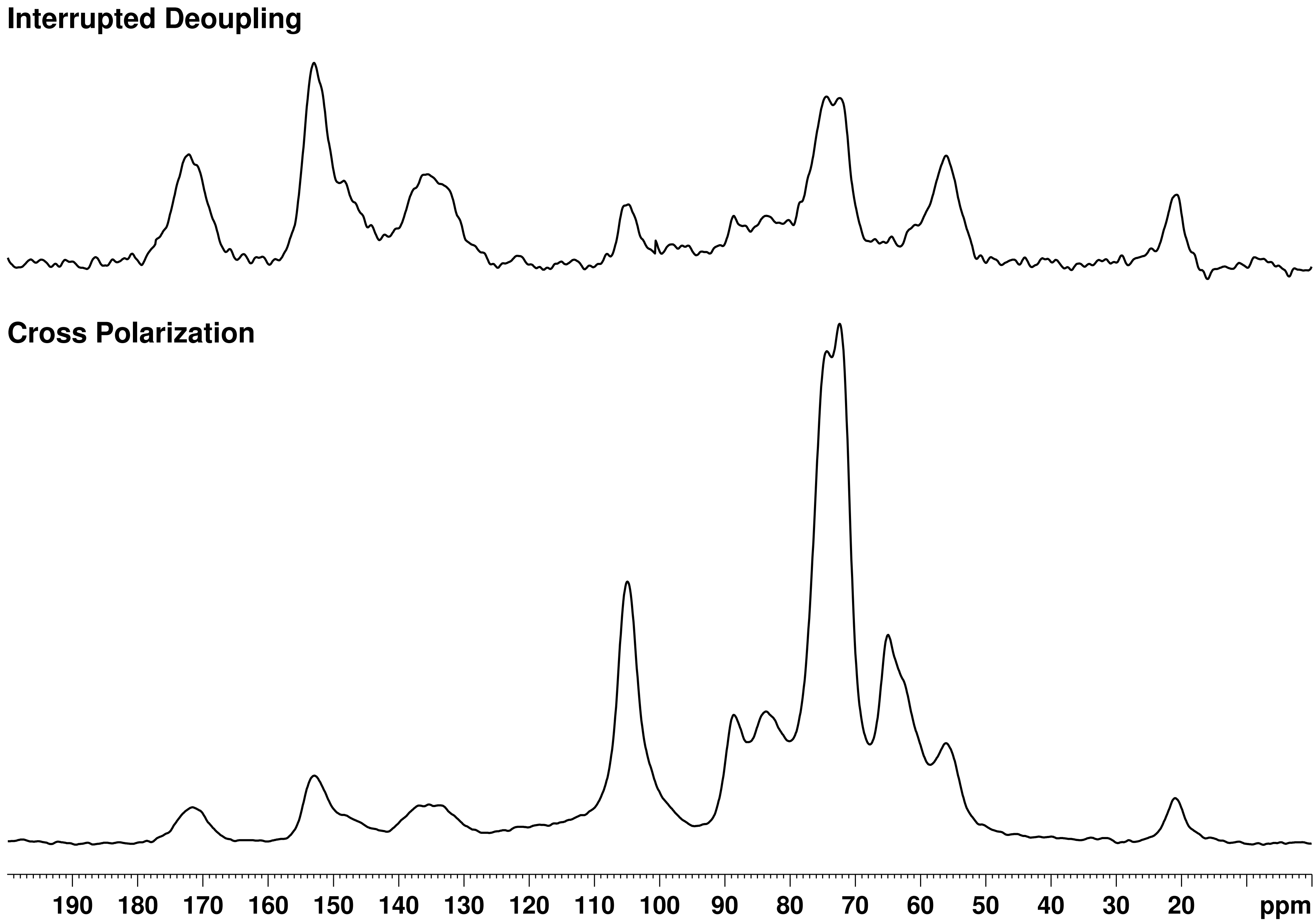
Interrupted Deoupling
Cross Polarization
190 180 170 160 150 140 130 120 110 100 90 80 70 60 50 40 30 20 ppm

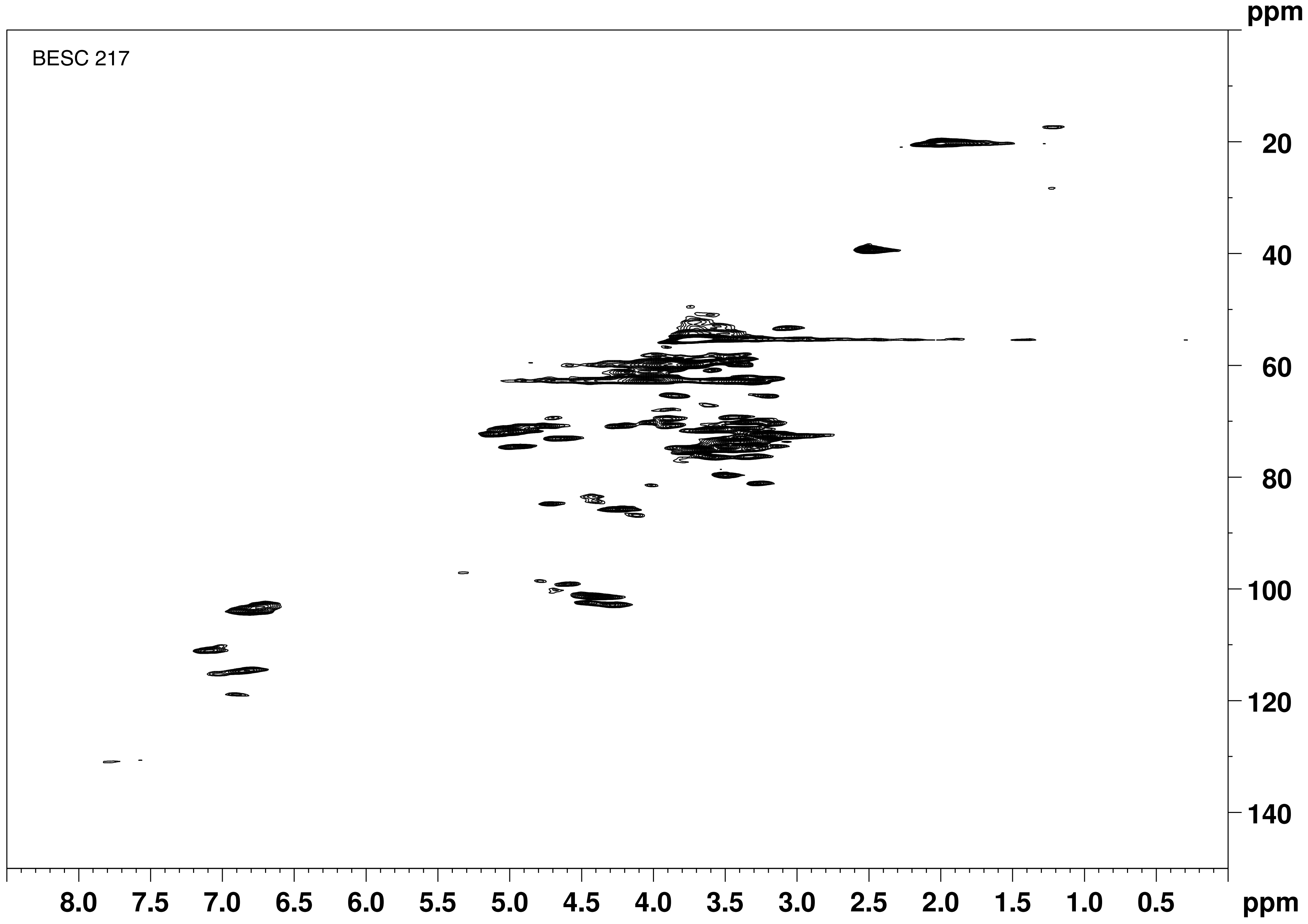

BESC 217
ppm
20
40
60
80
100
120
140
8.0
7.5
7.0
6.5
6.0
5.5
5.0
4.5
4.0
3.5
3.0
2.5
2.0
1.5
1.0
0.5
ppm

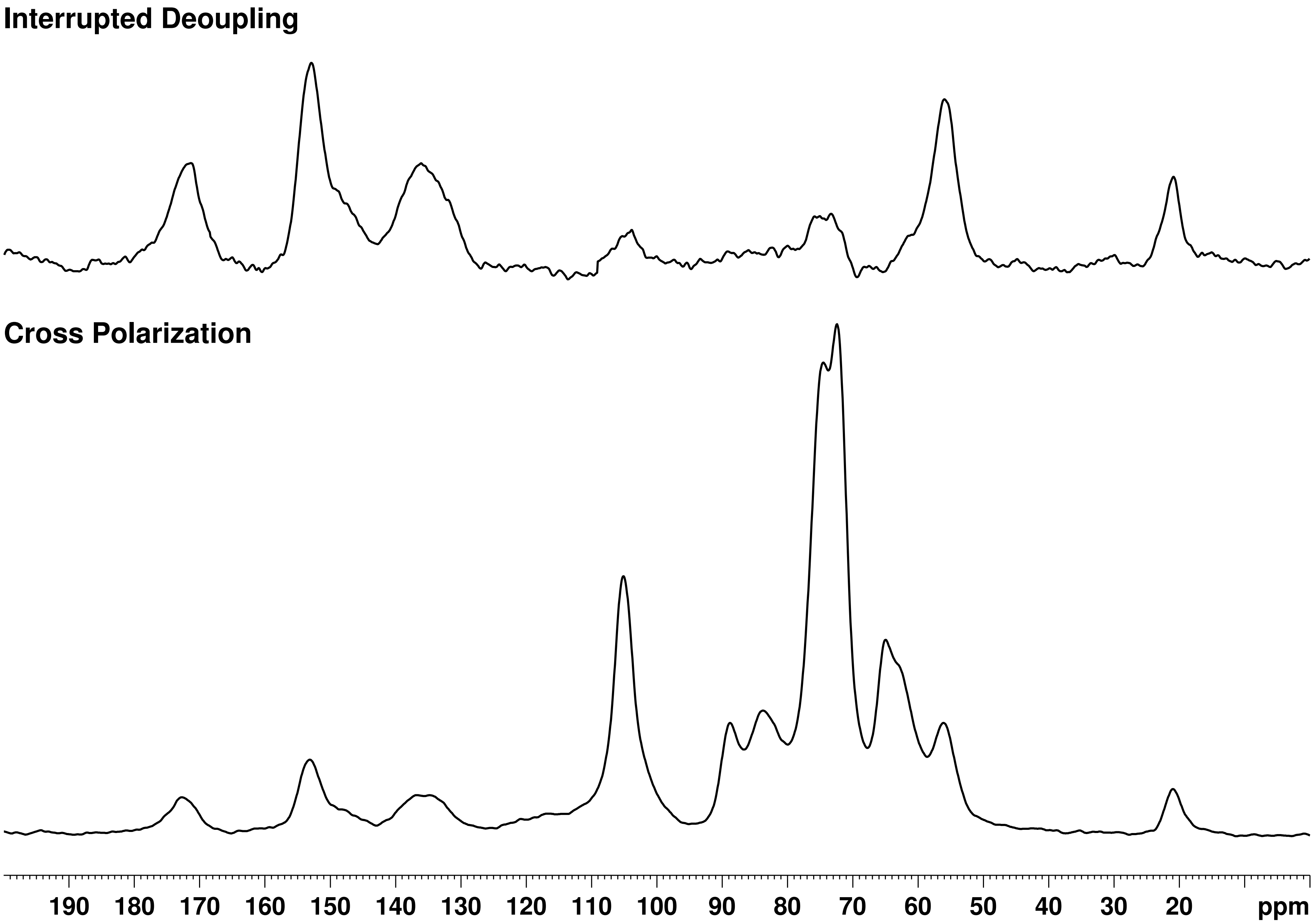
Interrupted Deoupling
Cross Polarization
190 180 170 160 150 140 130 120 110 100 90 80 70 60 50 40 30 20 ppm

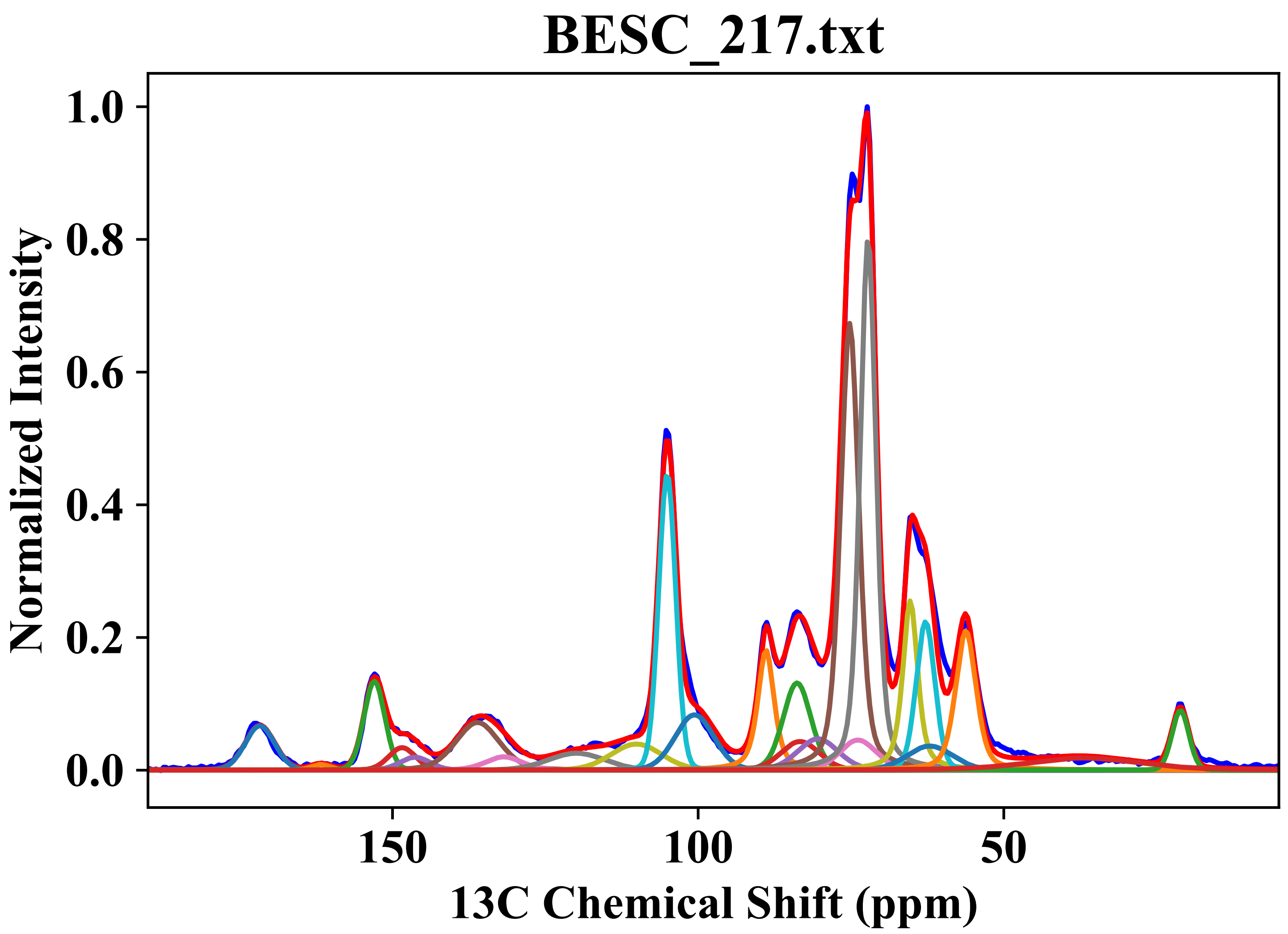
BESC_217.txt
Normalized Intensity
1.0
0.8
0.6
0.4
0.2
0.0
150
100
50
13C Chemical Shift (ppm)

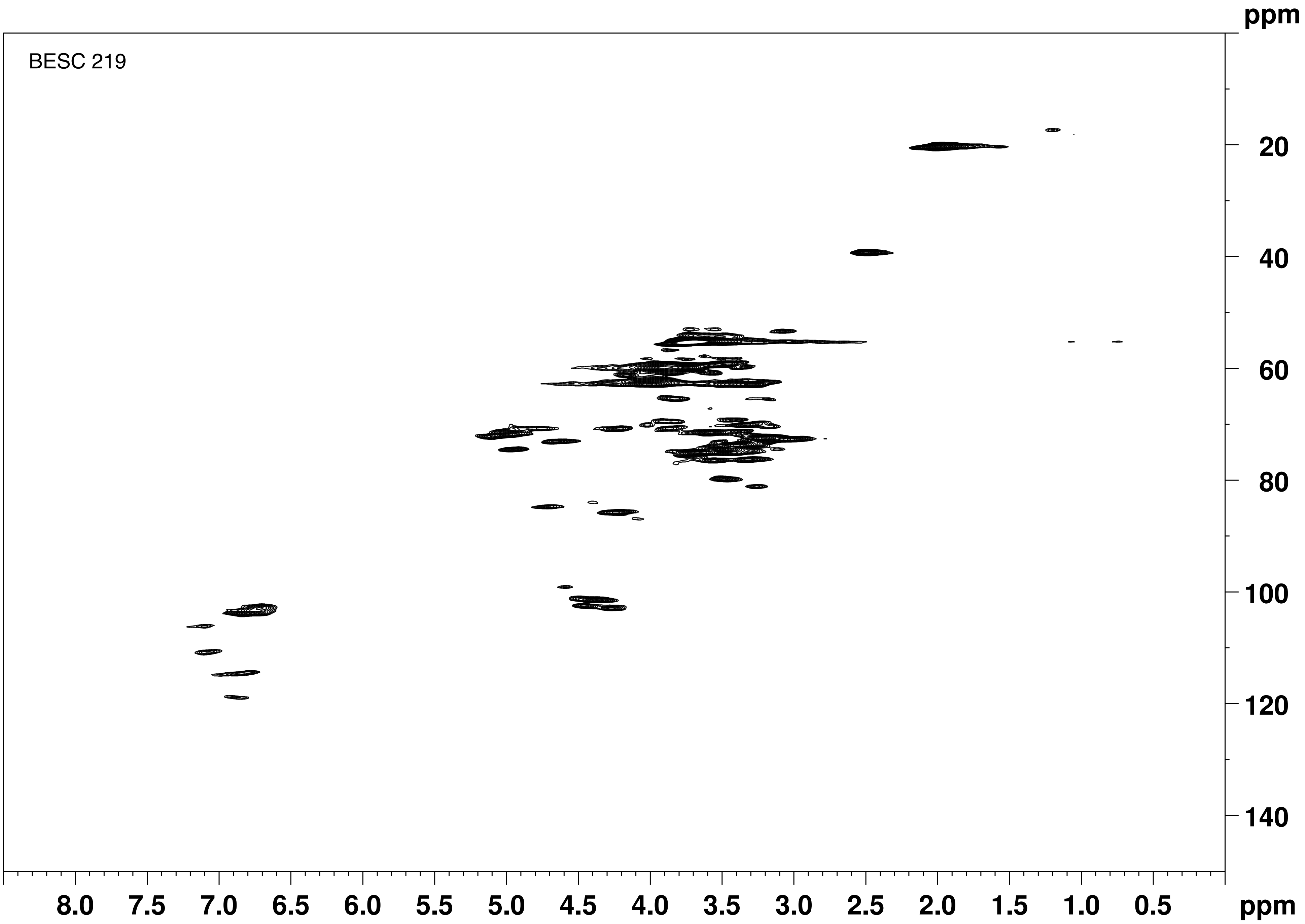

BESC 219
ppm
20
40
60
80
100
120
140
8.0
7.5
7.0
6.5
6.0
5.5
5.0
4.5
4.0
3.5
3.0
2.5
2.0
1.5
1.0
0.5
ppm

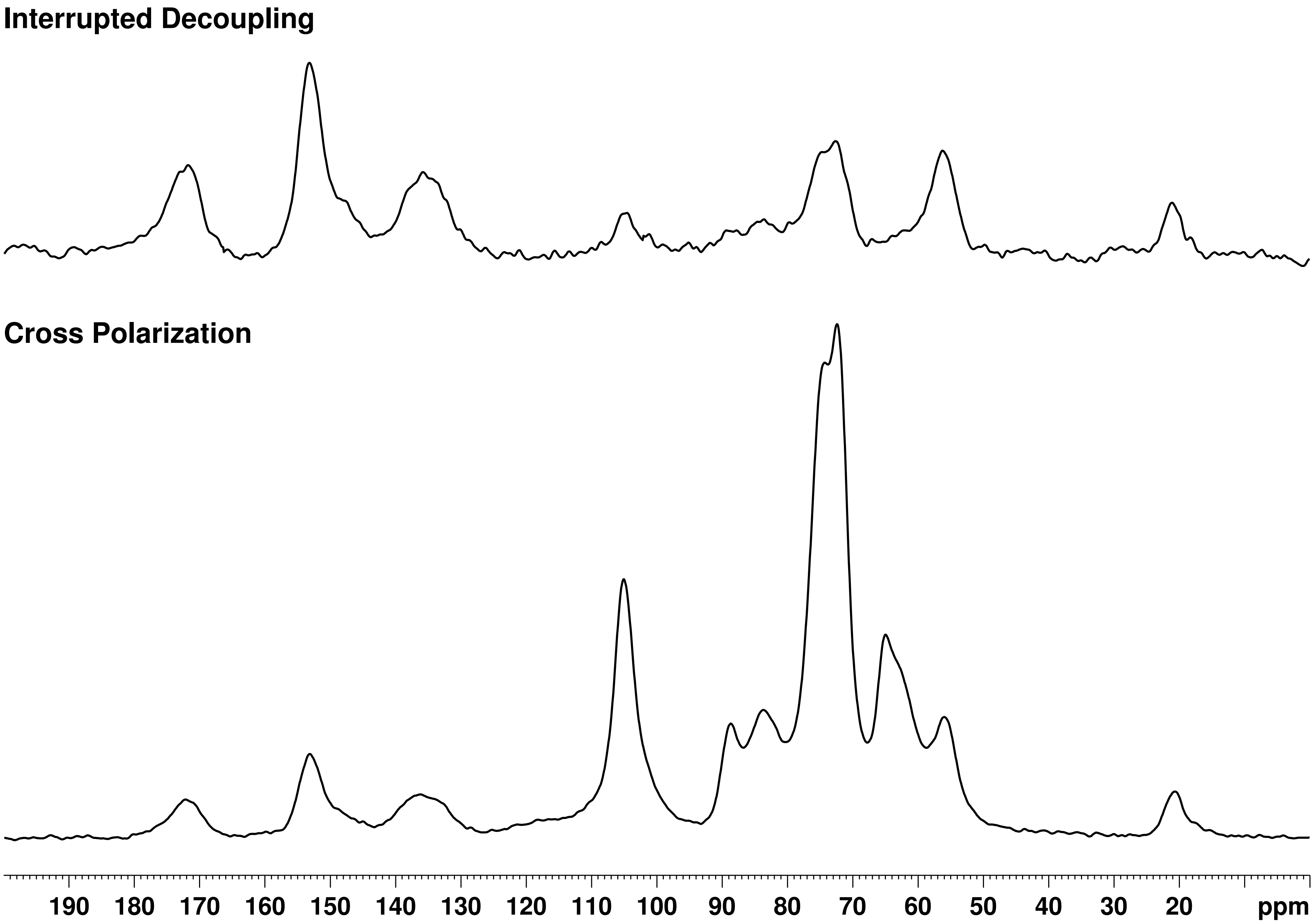
Interrupted Decoupling
Cross Polarization
190 180 170 160 150 140 130 120 110 100 90 80 70 60 50 40 30 20
ppm

BESC_219.txt

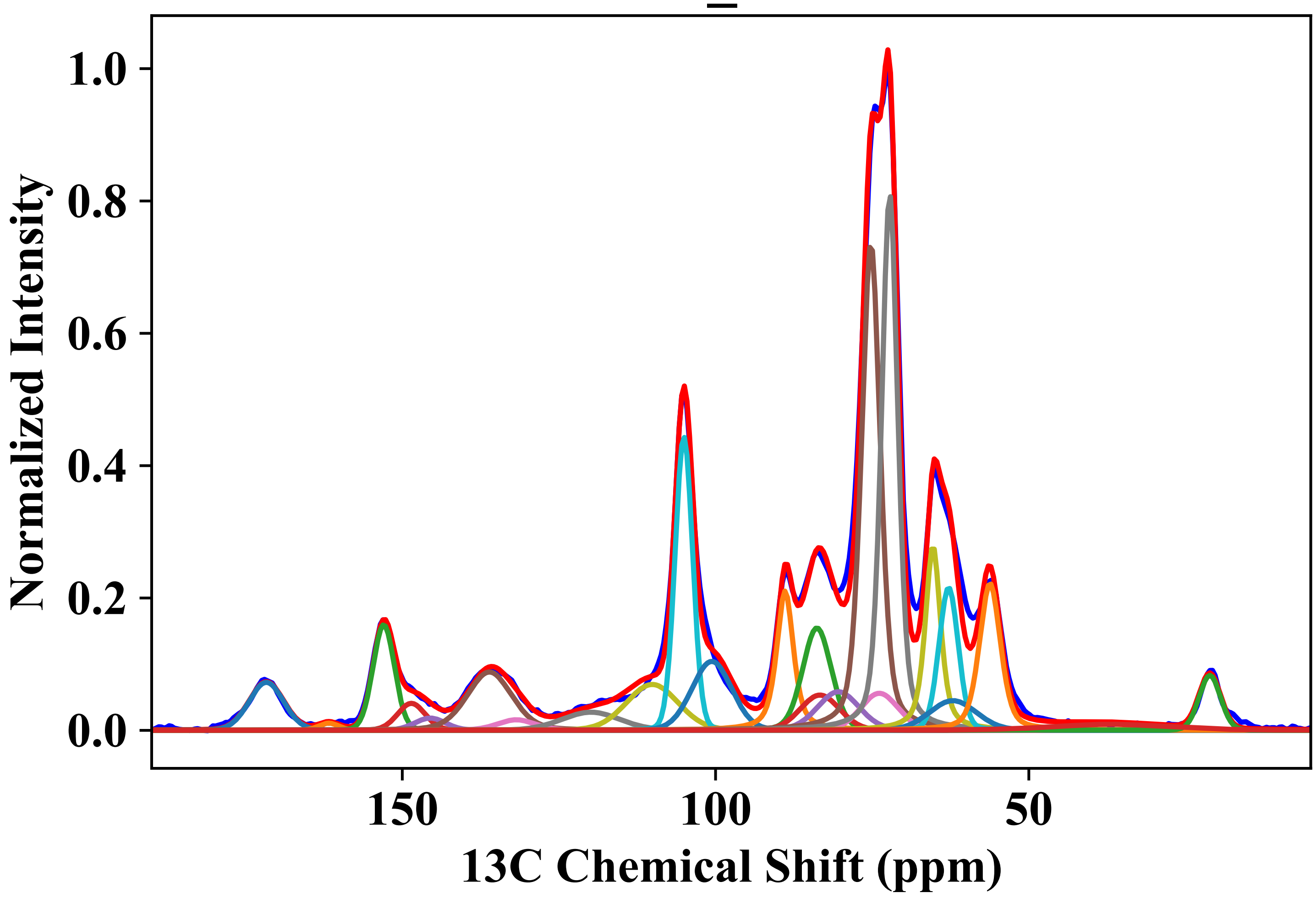

Normalized Intensity
1.0
0.8
0.6
0.4
0.2
0.0
150
100
50
13C Chemical Shift (ppm)

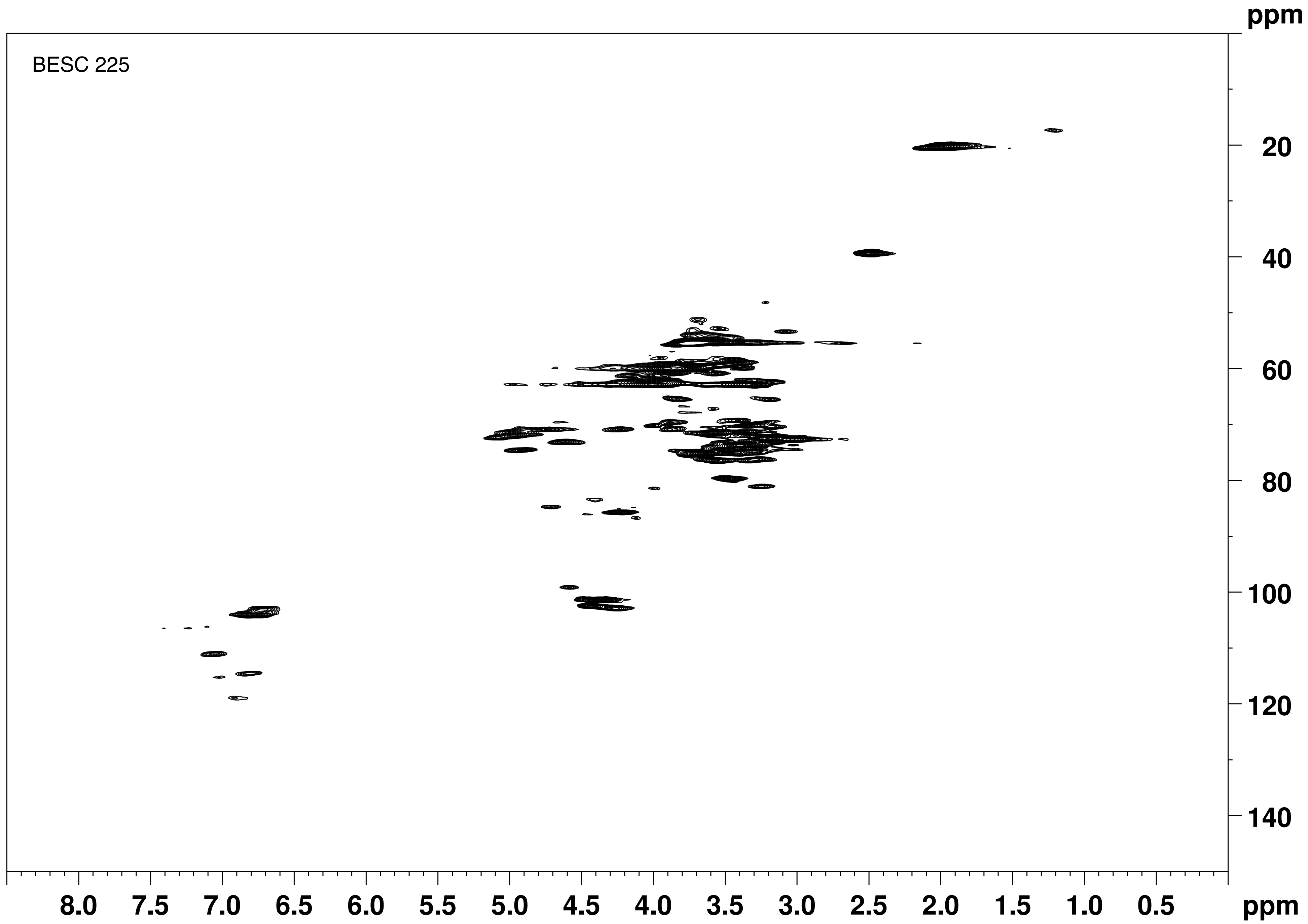
BESC 225
ppm
20
40
60
80
100
120
140
8.0 7.5 7.0 6.5 6.0 5.5 5.0 4.5 4.0 3.5 3.0 2.5 2.0 1.5 1.0 0.5
ppm

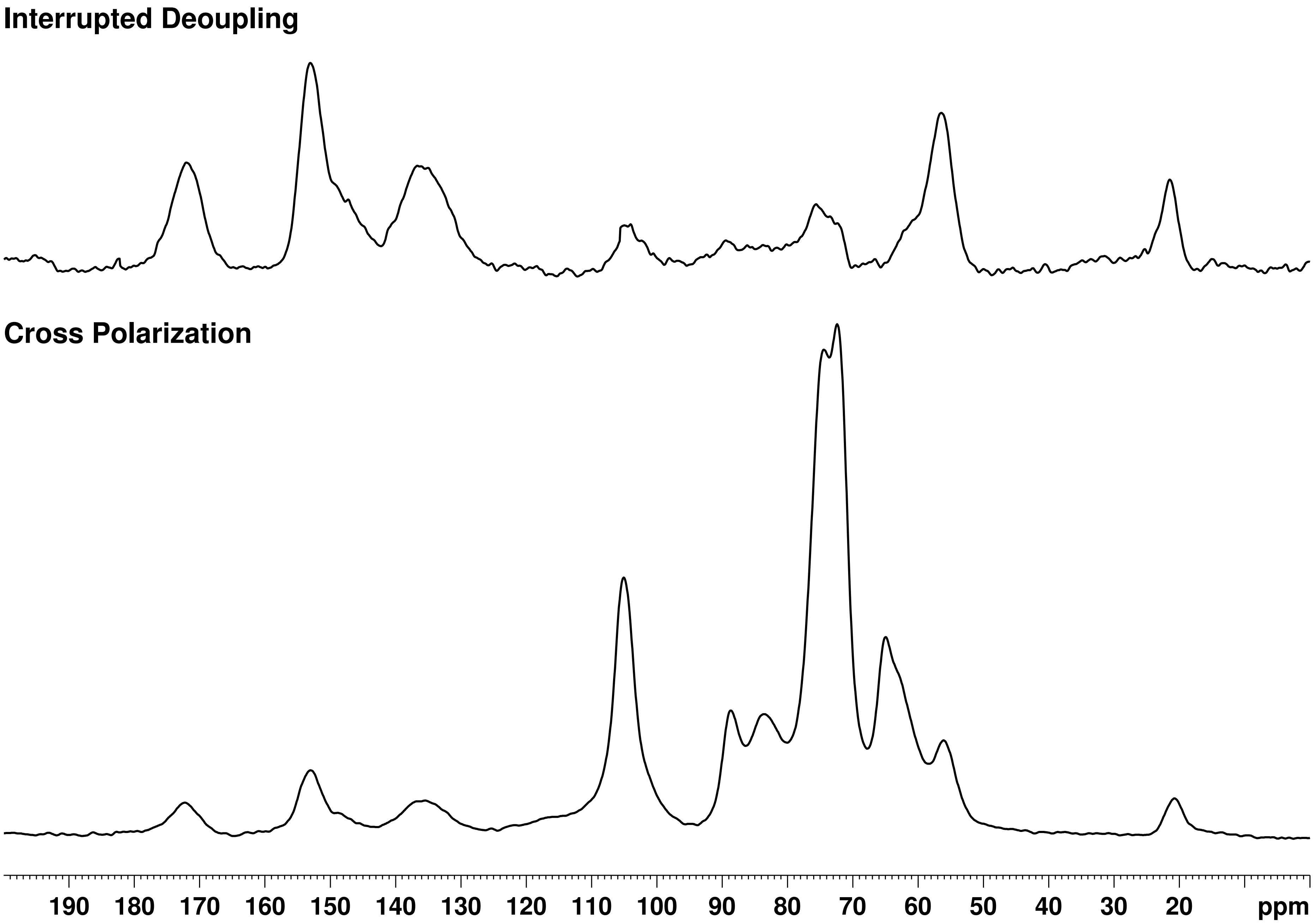

Interrupted Deoupling
Cross Polarization
190 180 170 160 150 140 130 120 110 100 90 80 70 60 50 40 30 20
ppm

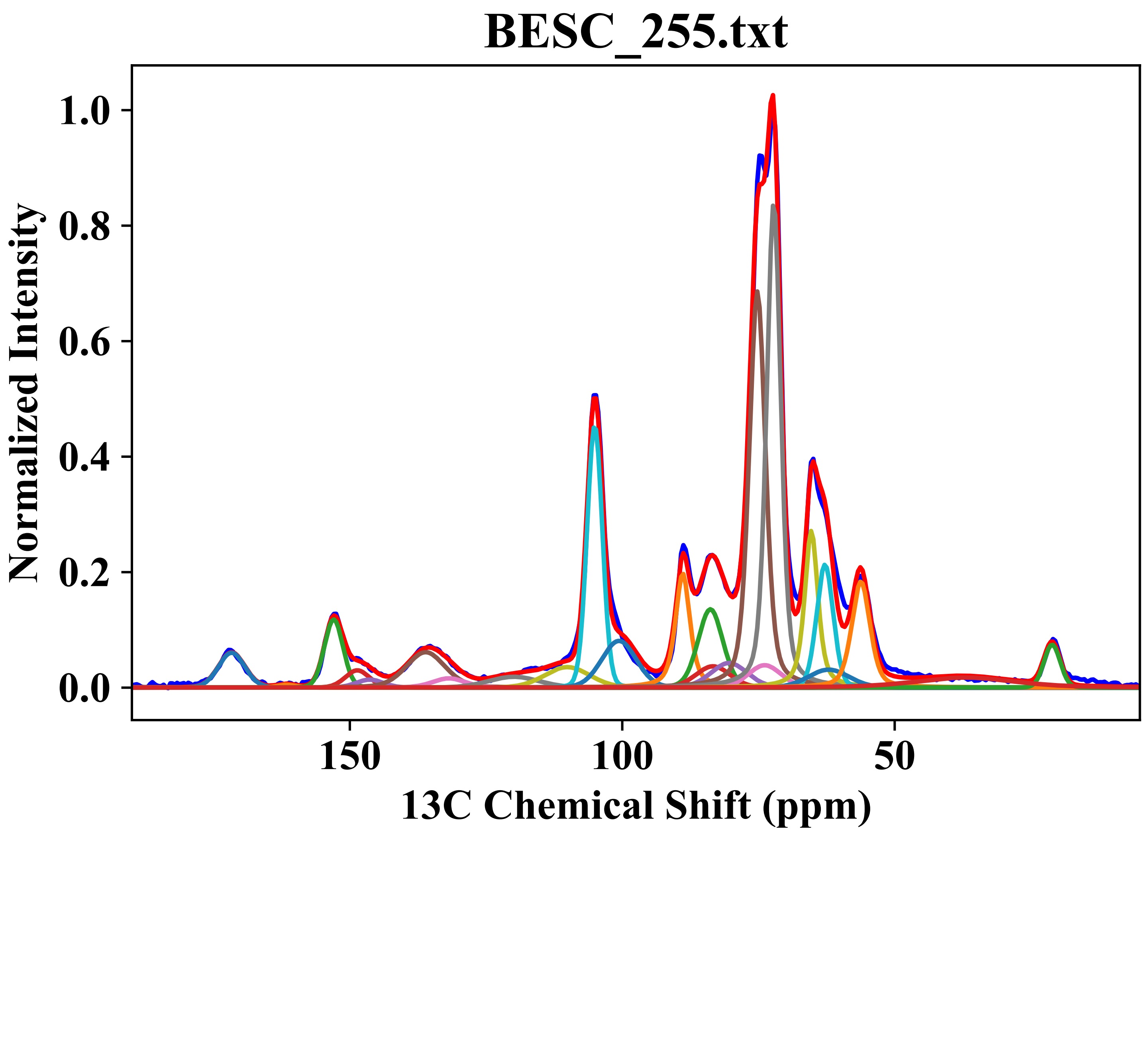

BESC_255.txt
Normalized Intensity
0.0
0.2
0.4
0.6
0.8
1.0
150
100
50
13C Chemical Shift (ppm)

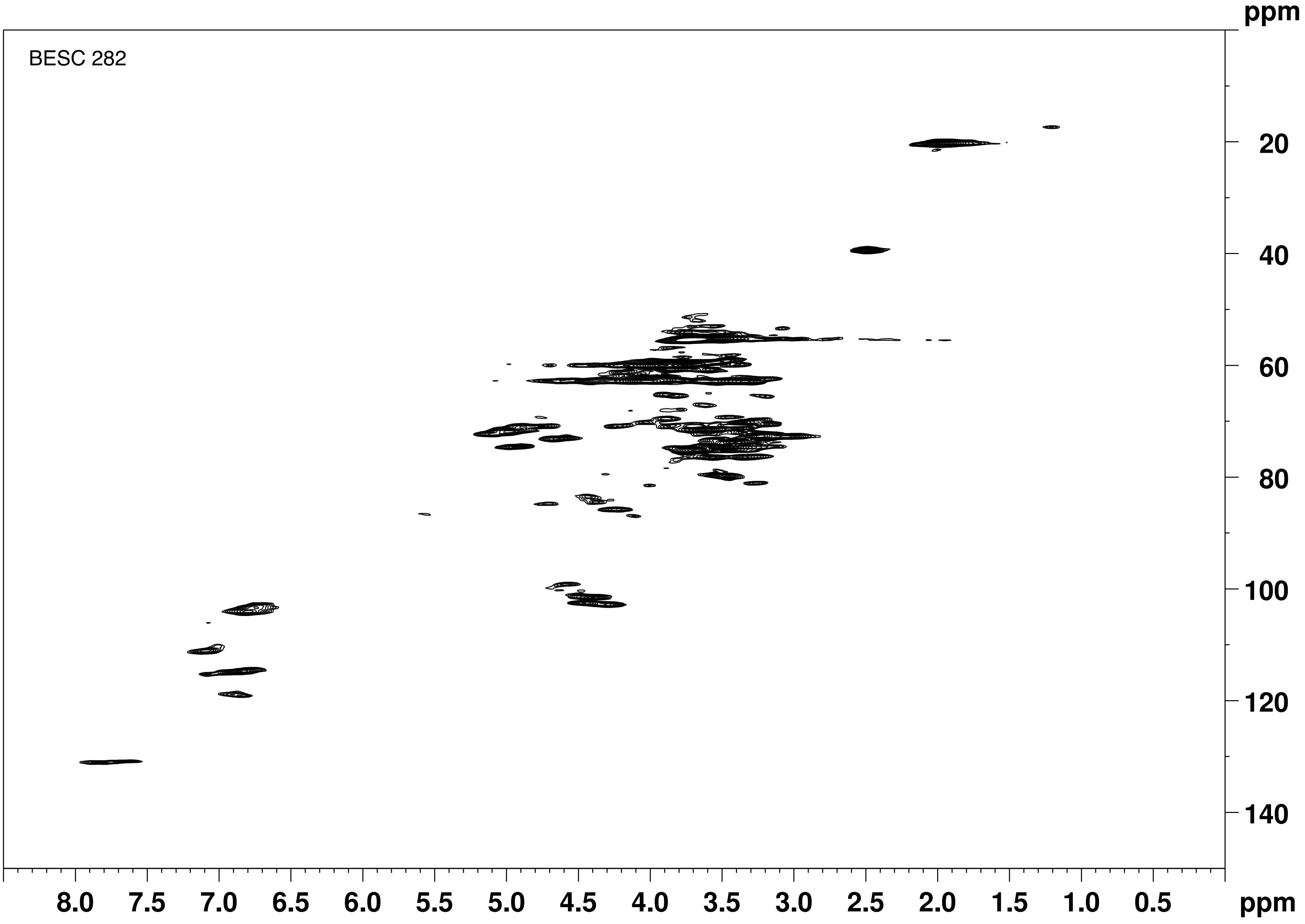
BESC 282
ppm
20
40
60
80
100
120
140
8.0 7.5 7.0 6.5 6.0 5.5 5.0 4.5 4.0 3.5 3.0 2.5 2.0 1.5 1.0 0.5
ppm

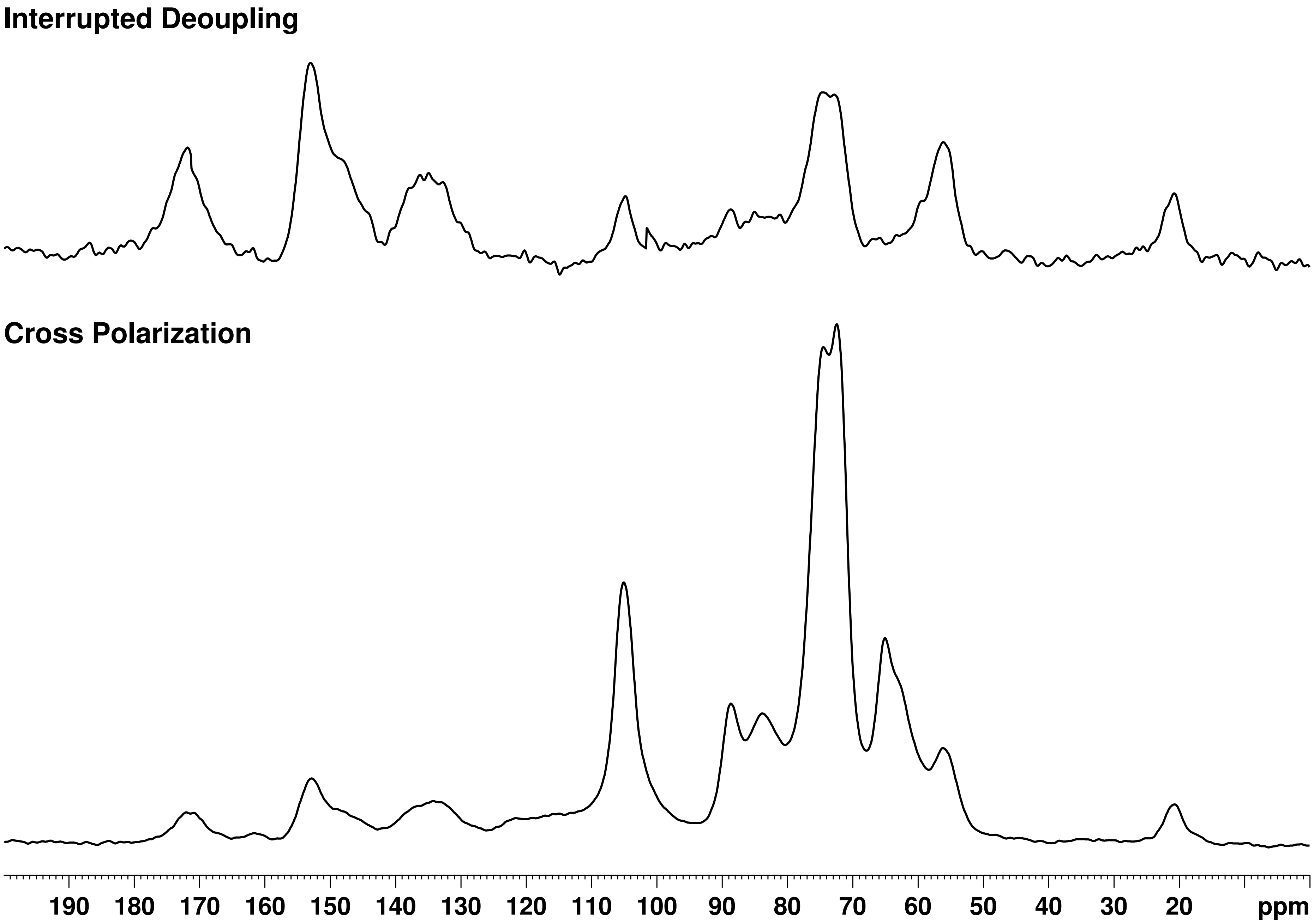
Interrupted Deoupling
Cross Polarization
190 180 170 160 150 140 130 120 110 100 90 80 70 60 50 40 30 20 ppm

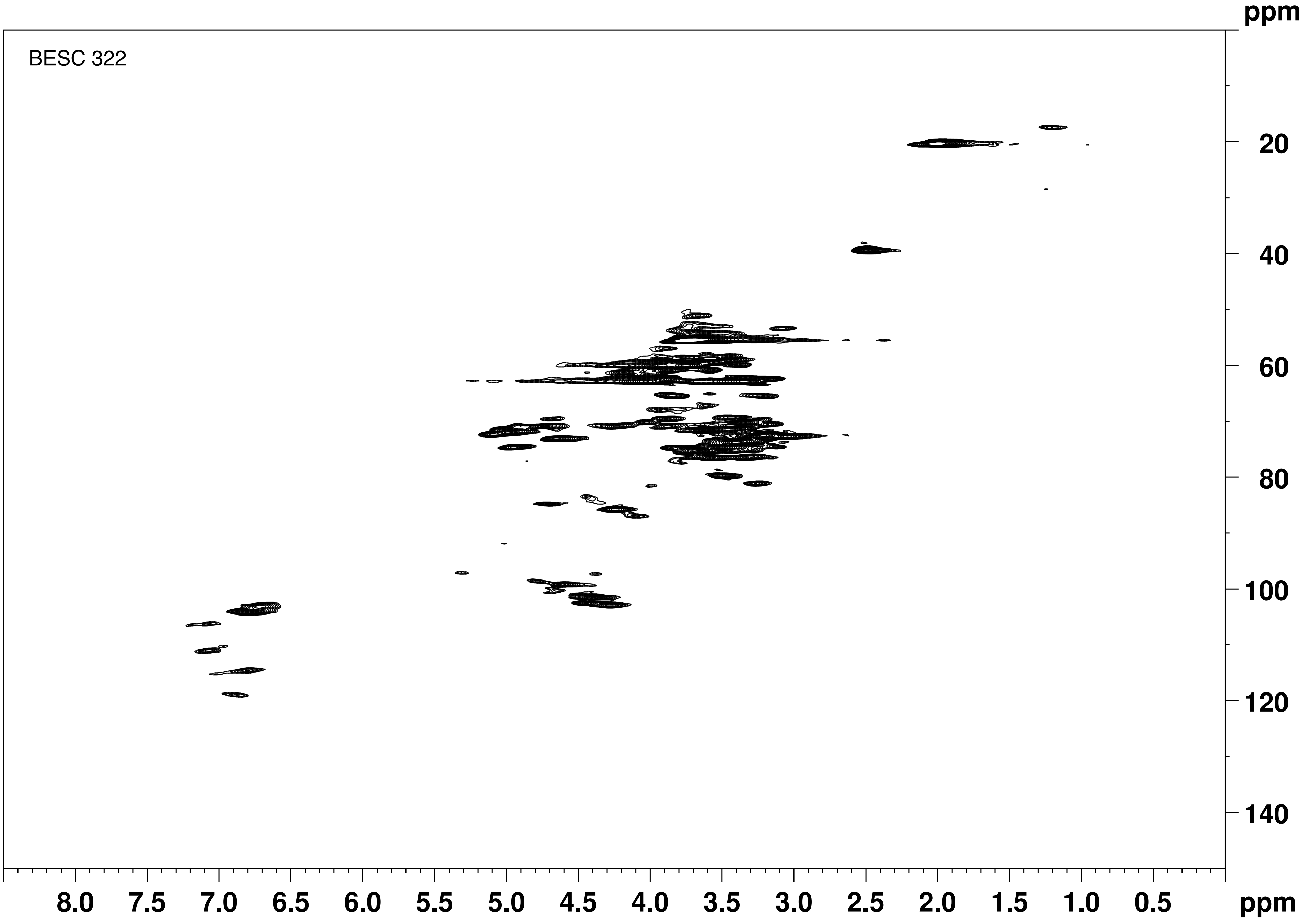

BESC 322
ppm
20
40
60
80
100
120
140
8.0 7.5 7.0 6.5 6.0 5.5 5.0 4.5 4.0 3.5 3.0 2.5 2.0 1.5 1.0 0.5
ppm

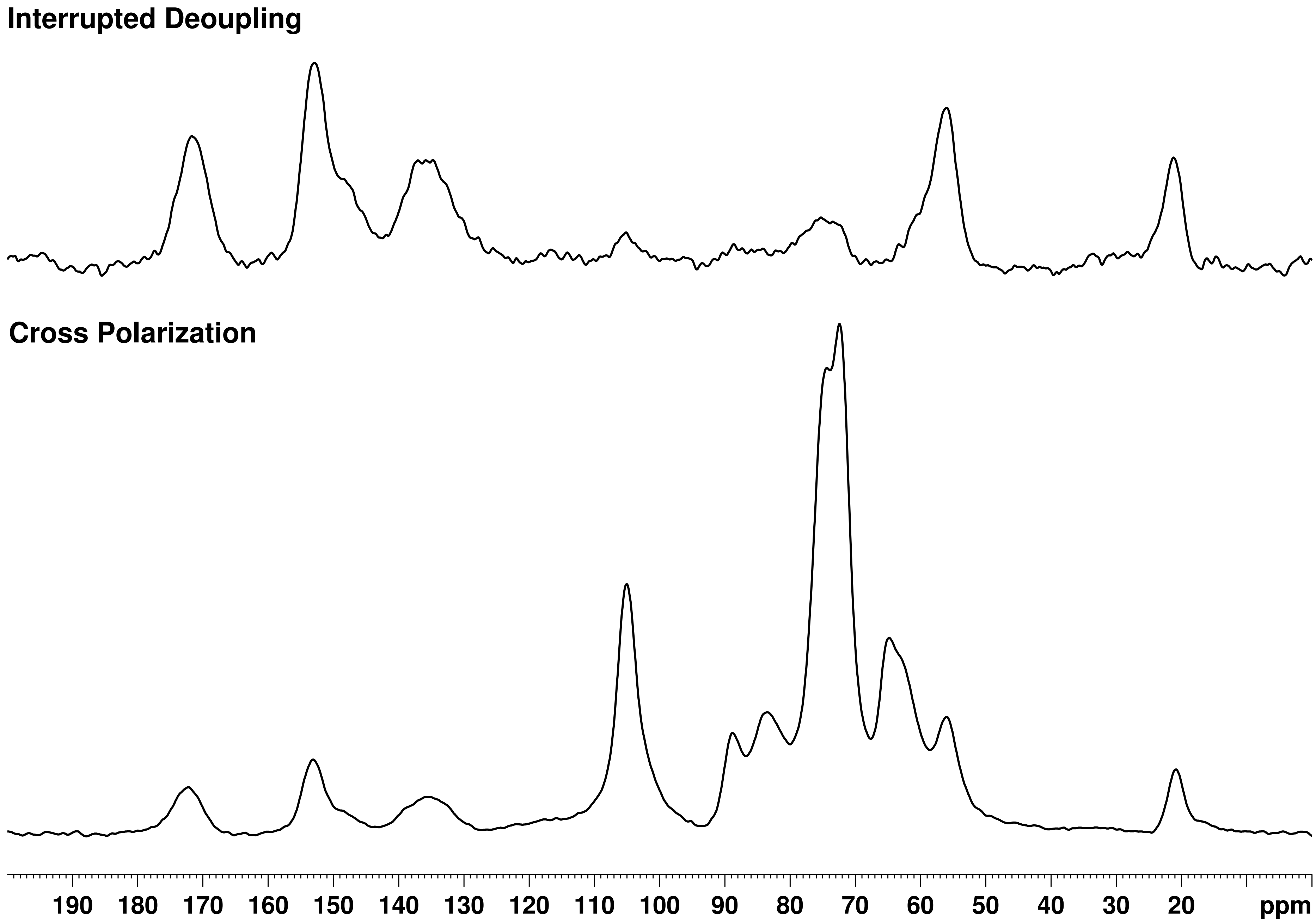

Interrupted Deoupling
Cross Polarization
190 180 170 160 150 140 130 120 110 100 90 80 70 60 50 40 30 20 ppm

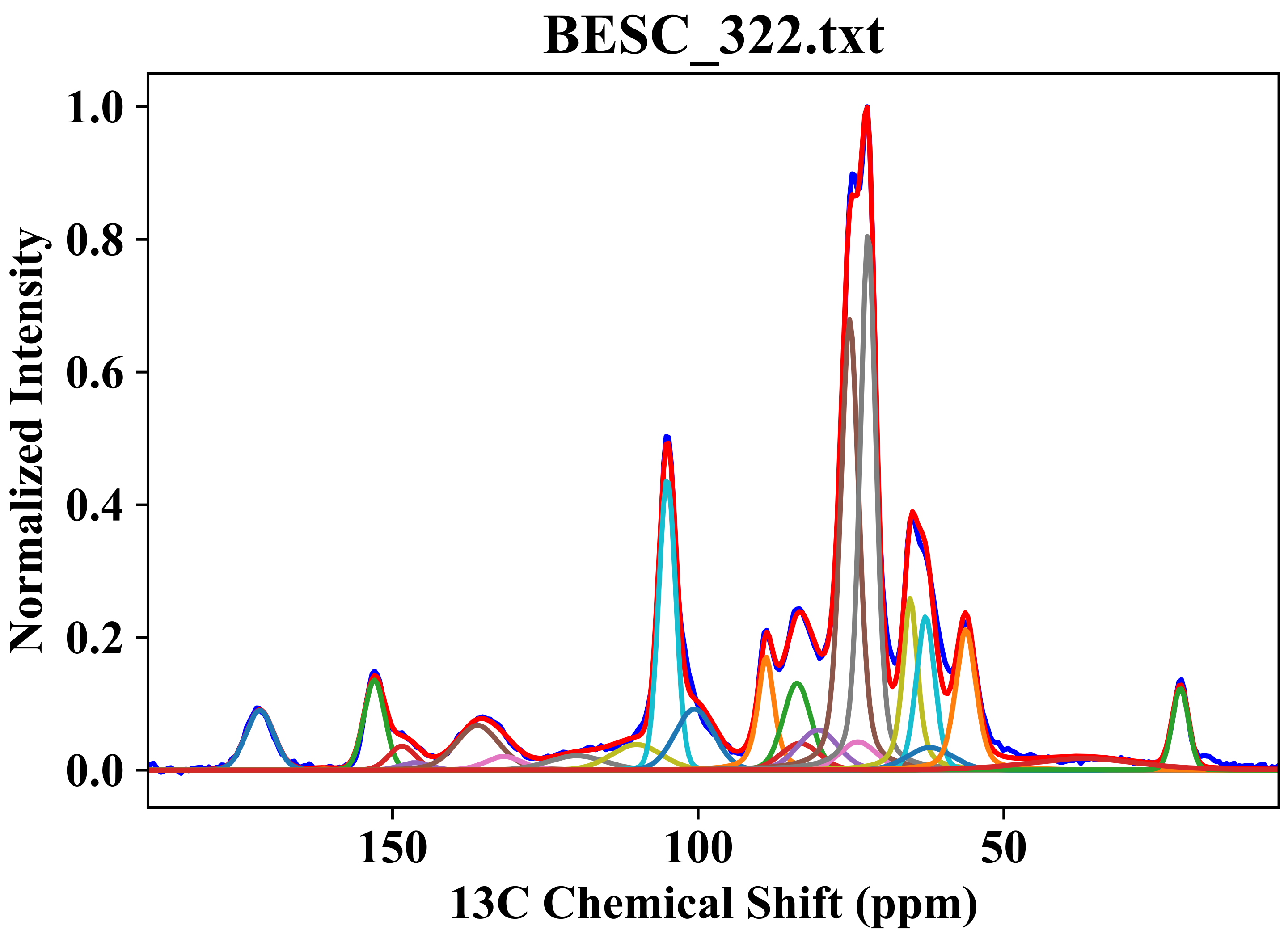
BESC_322.txt
Normalized Intensity
0.0
0.2
0.4
0.6
0.8
1.0
150
100
50
13C Chemical Shift (ppm)

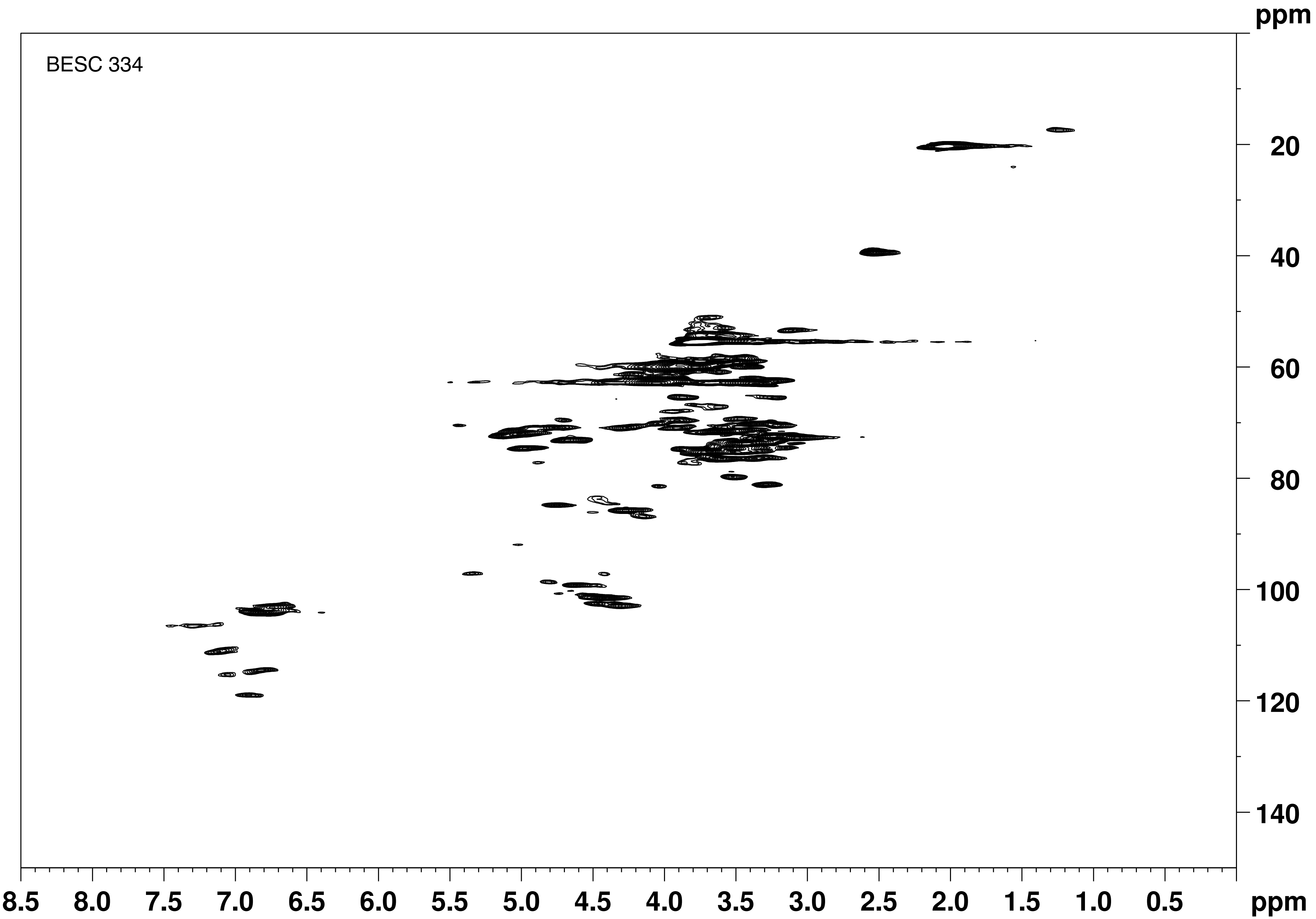

BESC 334
ppm
20
40
60
80
100
120
140
8.5
8.0
7.5
7.0
6.5
6.0
5.5
5.0
4.5
4.0
3.5
3.0
2.5
2.0
1.5
1.0
0.5
ppm

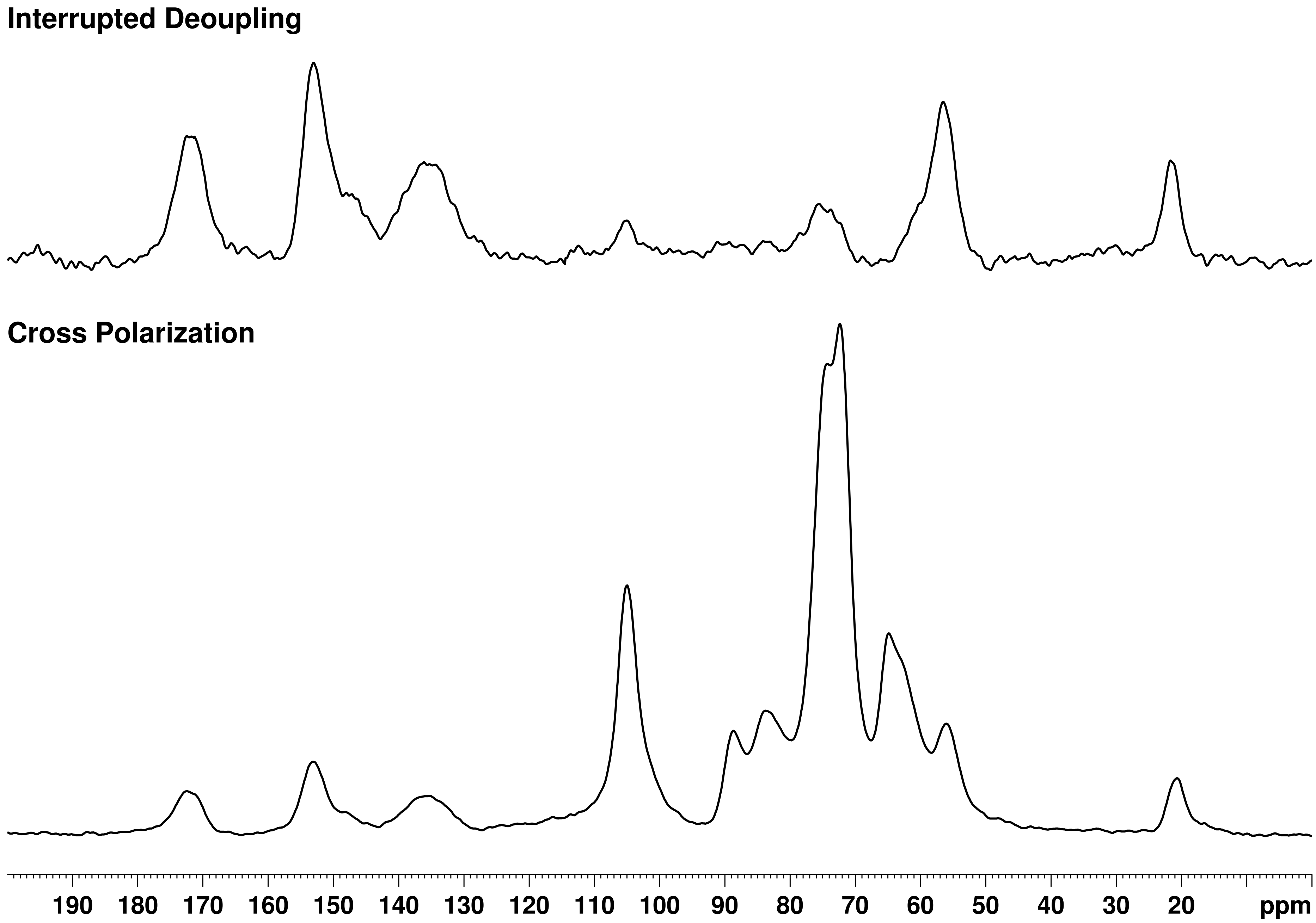
Interrupted Deoupling
Cross Polarization
190 180 170 160 150 140 130 120 110 100 90 80 70 60 50 40 30 20 ppm

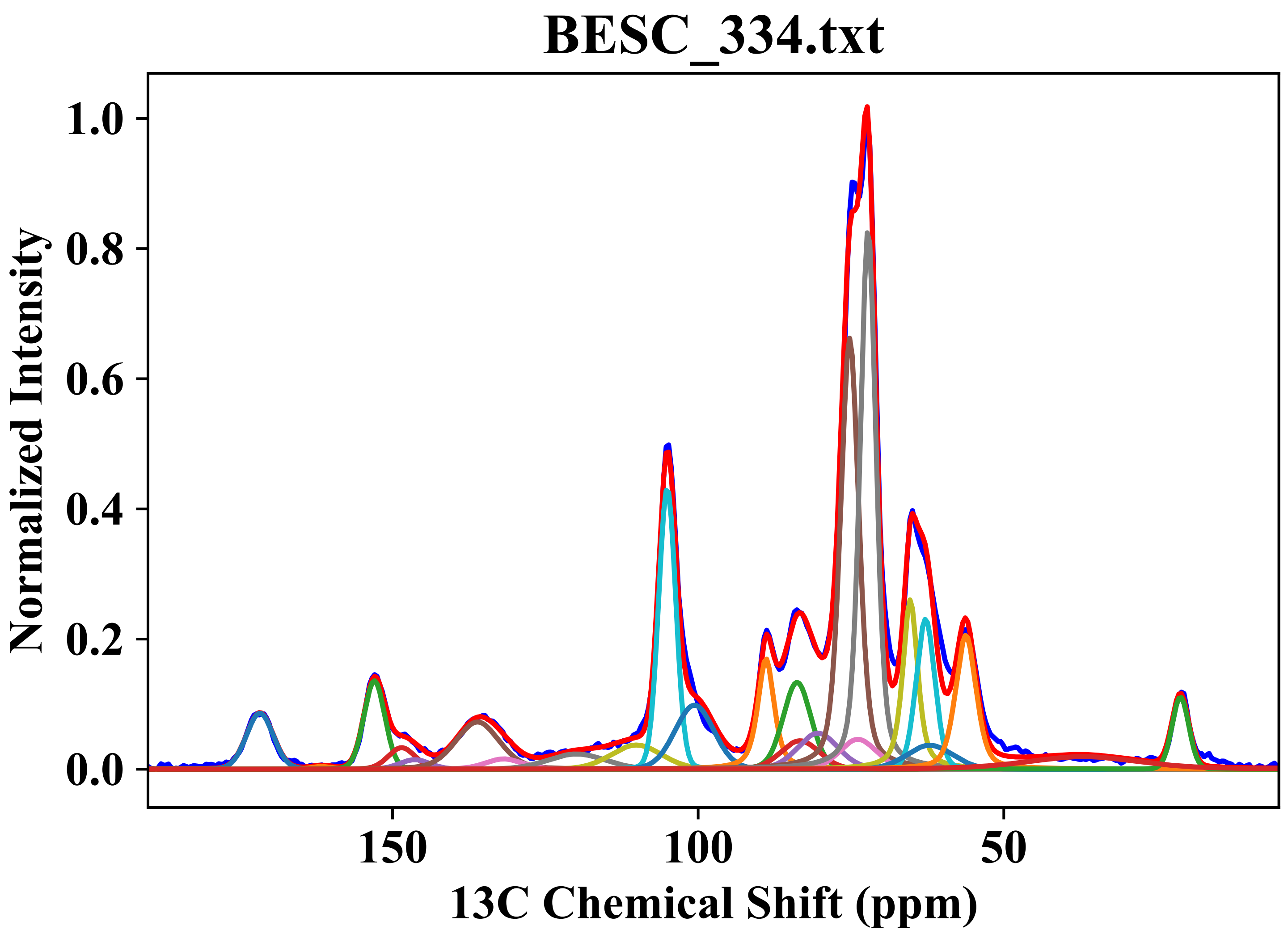
BESC_334.txt
Normalized Intensity
0.0
0.2
0.4
0.6
0.8
1.0
150
100
50
13C Chemical Shift (ppm)

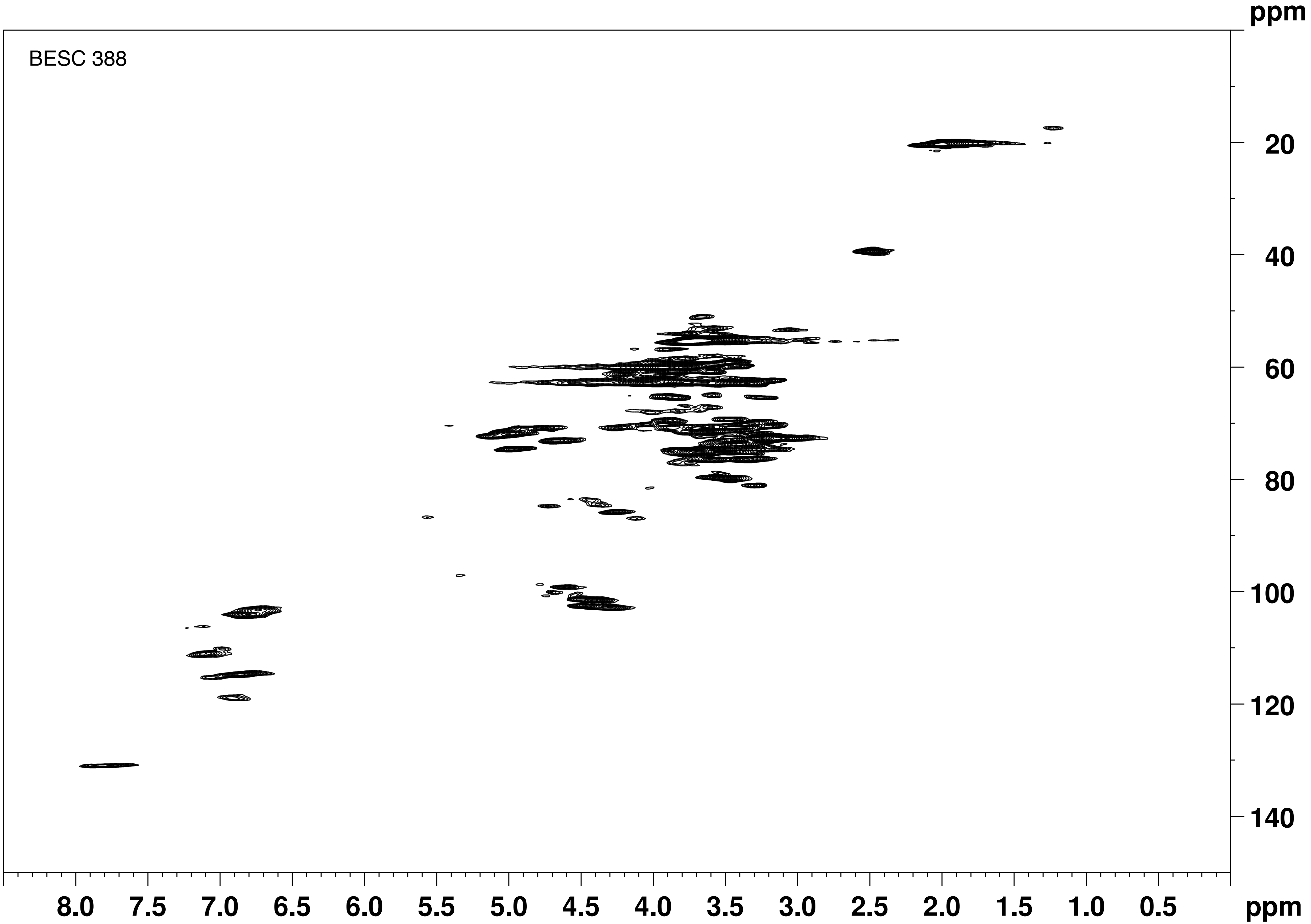
BESC 388
ppm
20
40
60
80
100
120
140
8.0
7.5
7.0
6.5
6.0
5.5
5.0
4.5
4.0
3.5
3.0
2.5
2.0
1.5
1.0
0.5
ppm

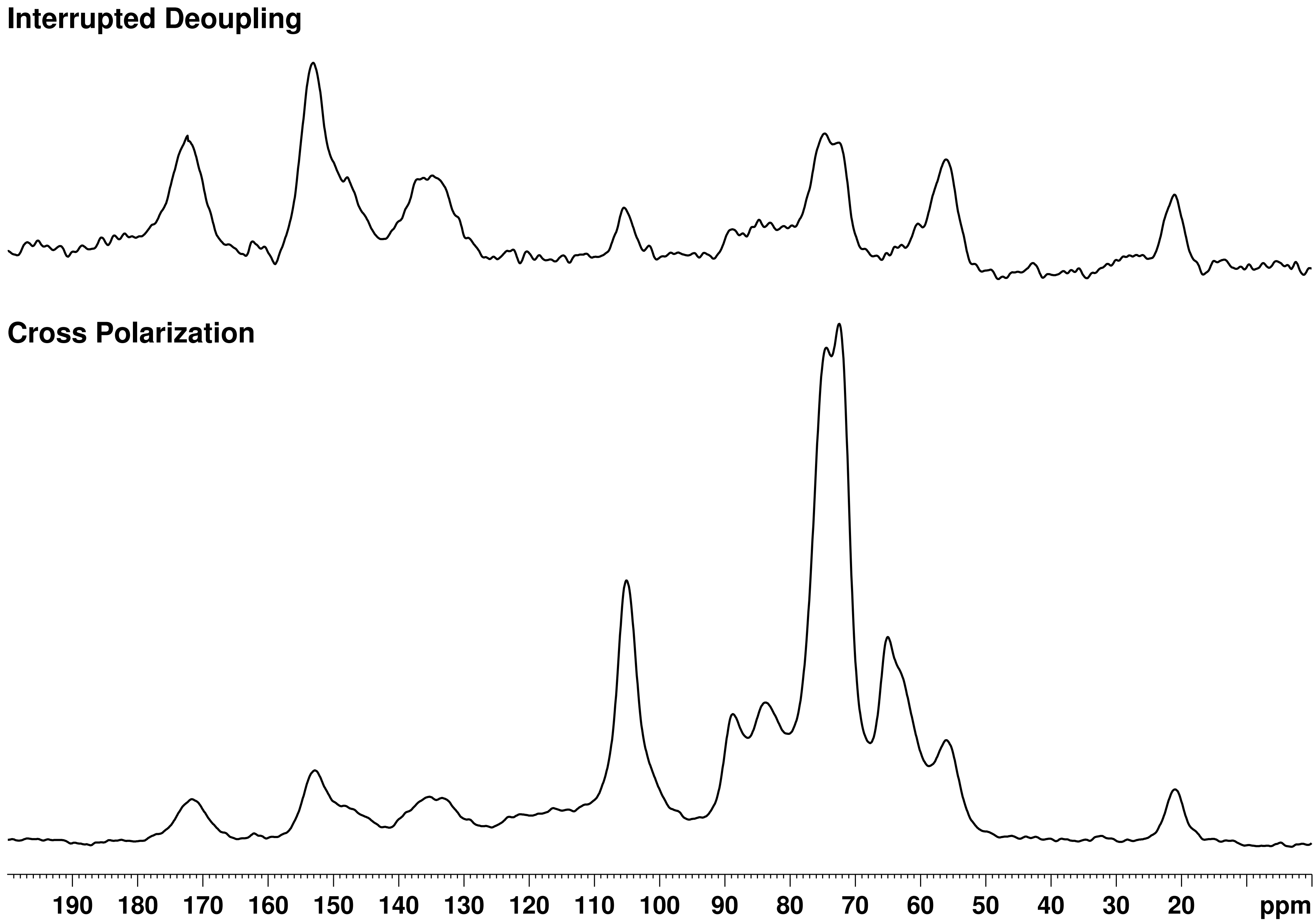
Interrupted Deoupling
Cross Polarization
190 180 170 160 150 140 130 120 110 100 90 80 70 60 50 40 30 20 ppm

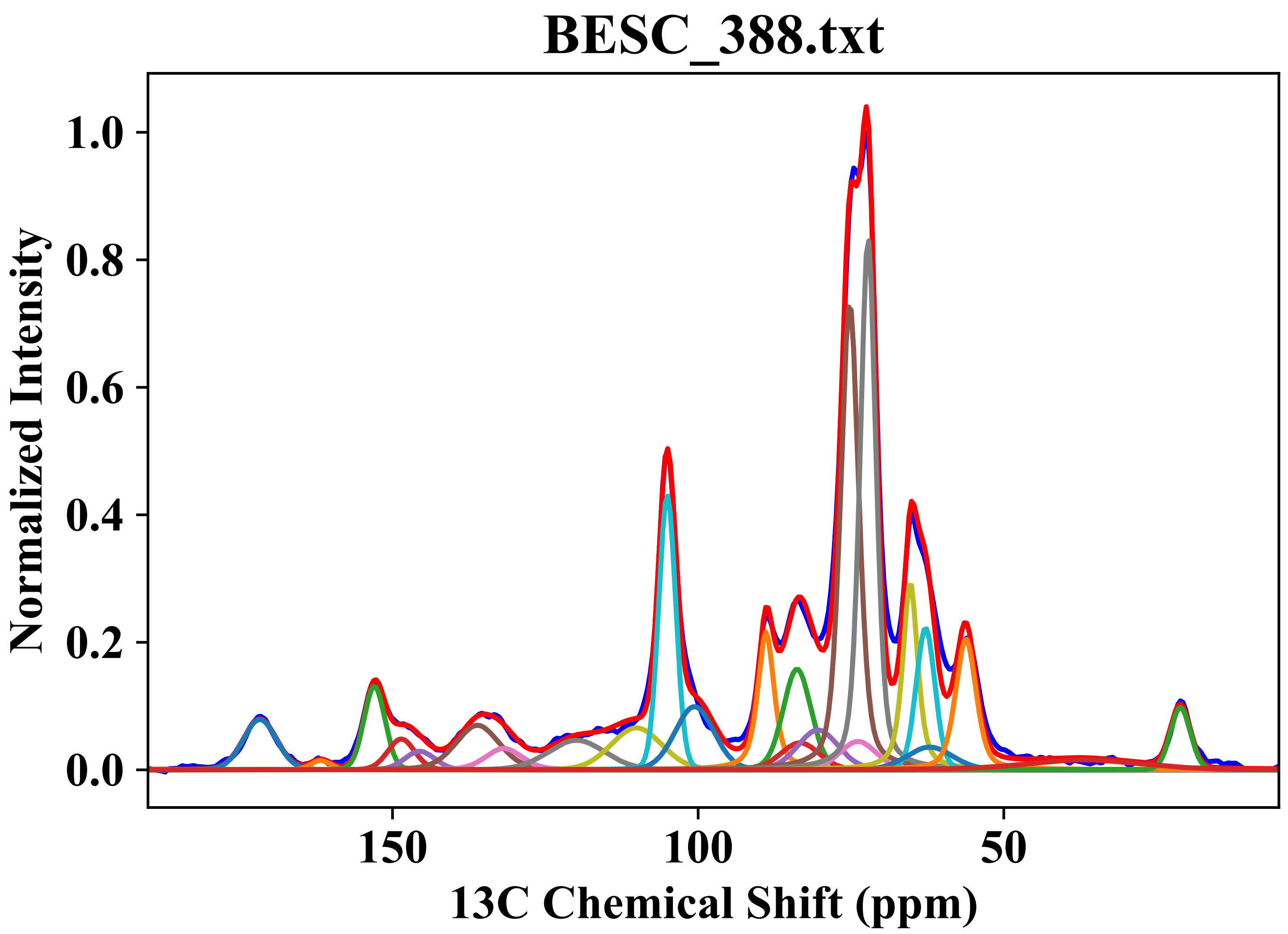
BESC_388.txt
Normalized Intensity
1.0
0.8
0.6
0.4
0.2
0.0
150
100
50
13C Chemical Shift (ppm)

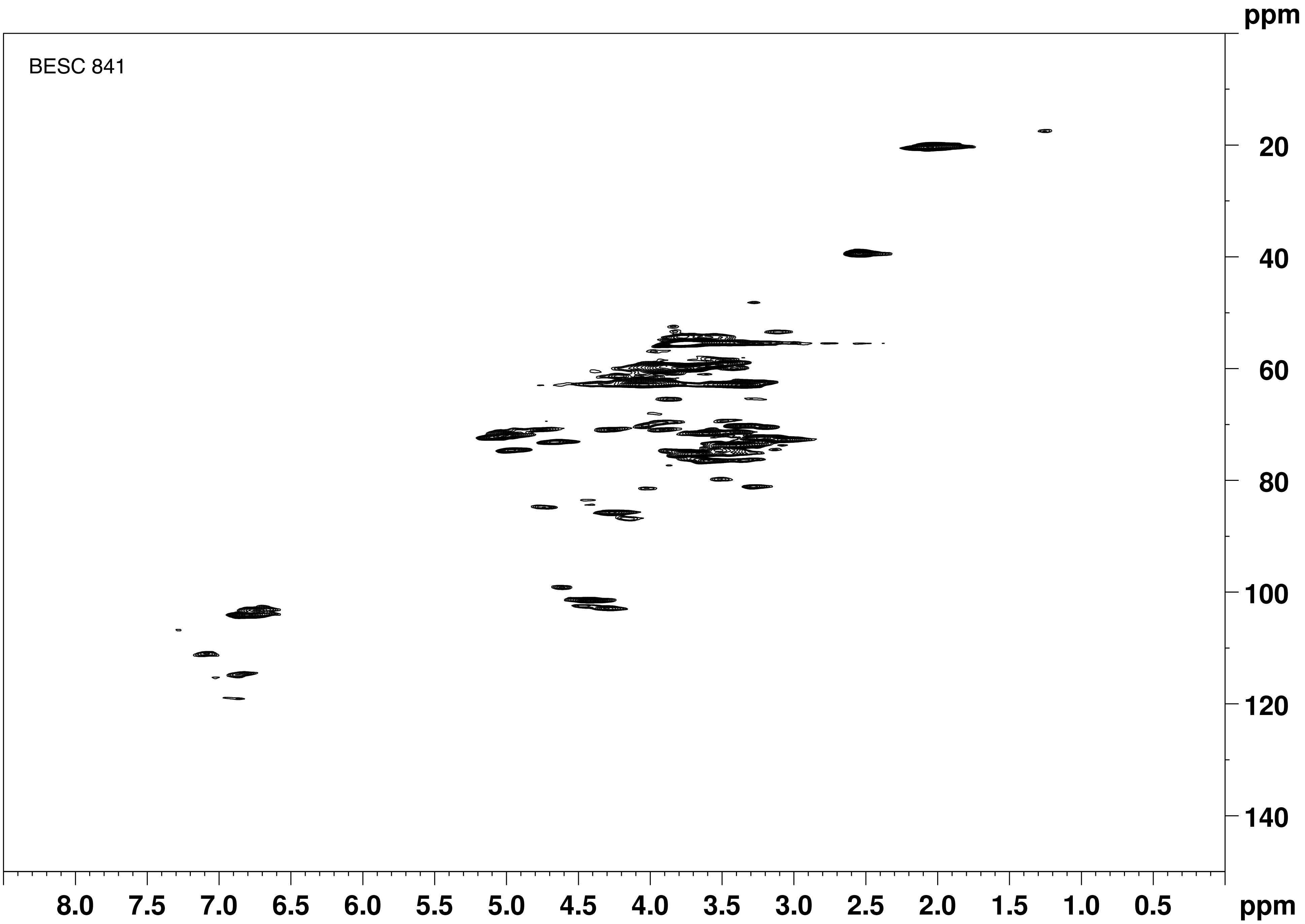
BESC 841
ppm
20
40
60
80
100
120
140
8.0
7.5
7.0
6.5
6.0
5.5
5.0
4.5
4.0
3.5
3.0
2.5
2.0
1.5
1.0
0.5
ppm

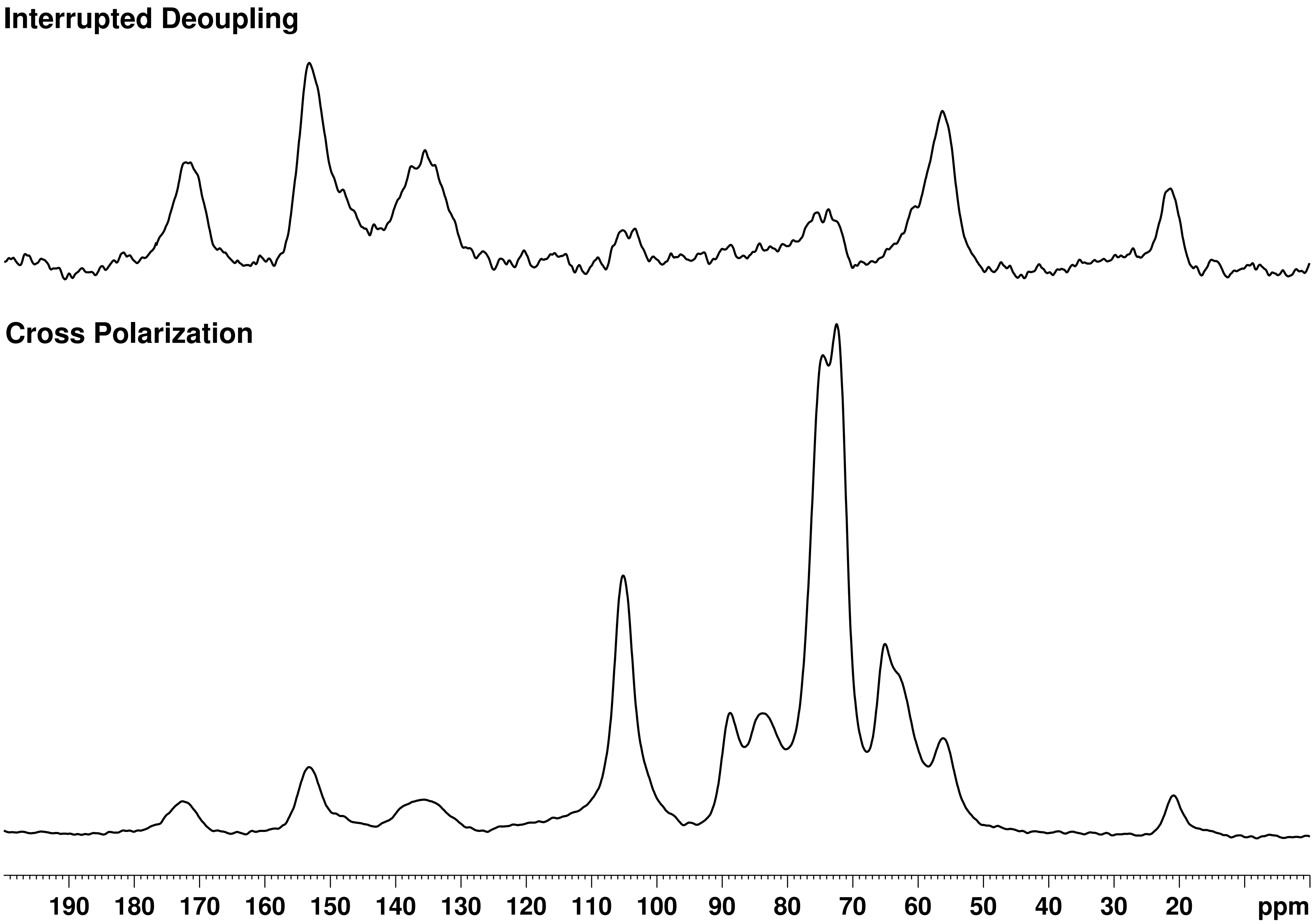
Interrupted Deoupling
Cross Polarization
190 180 170 160 150 140 130 120 110 100 90 80 70 60 50 40 30 20 ppm

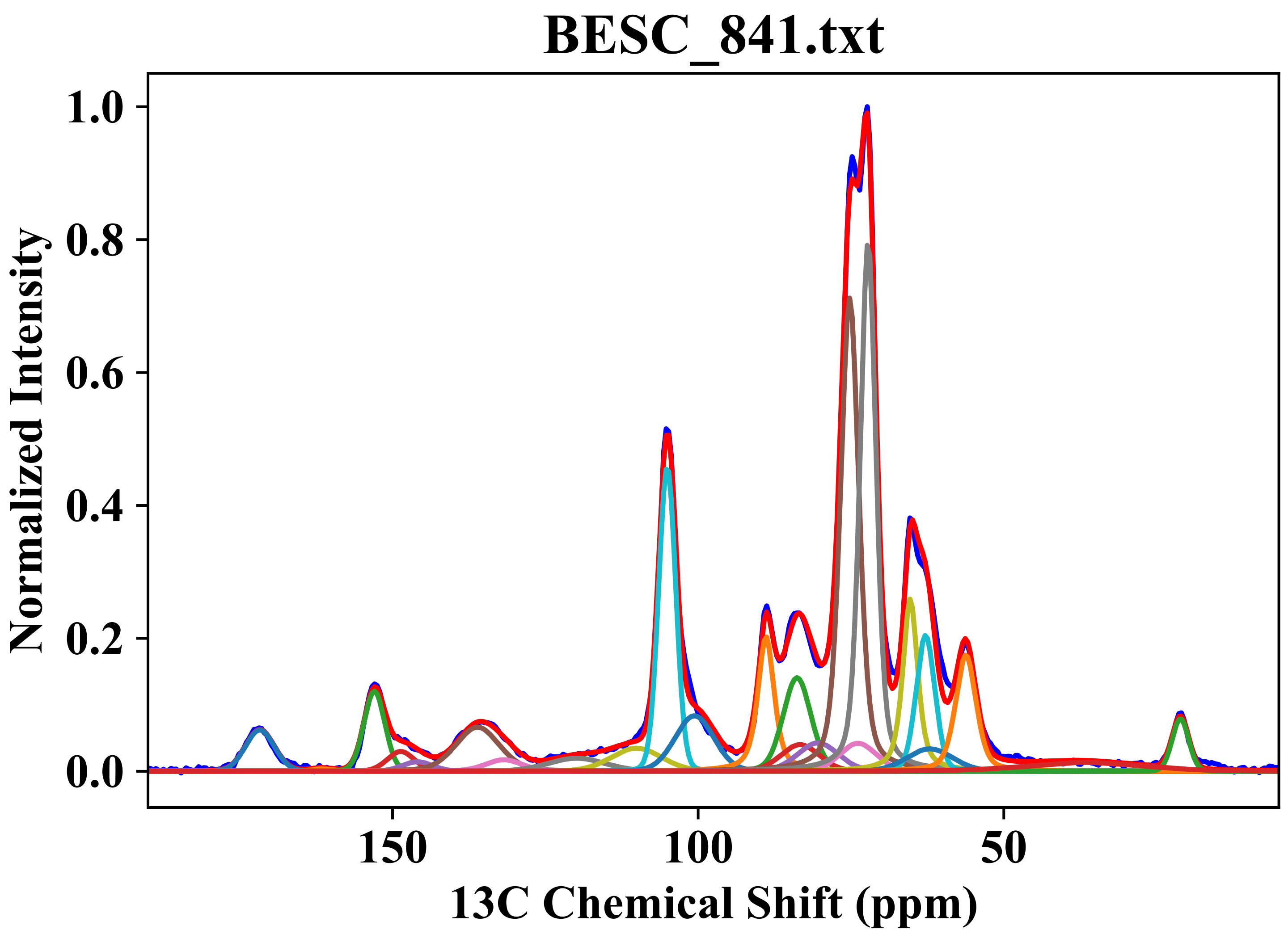

BESC_841.txt
Normalized Intensity
1.0
0.8
0.6
0.4
0.2
0.0
150
100
50
13C Chemical Shift (ppm)

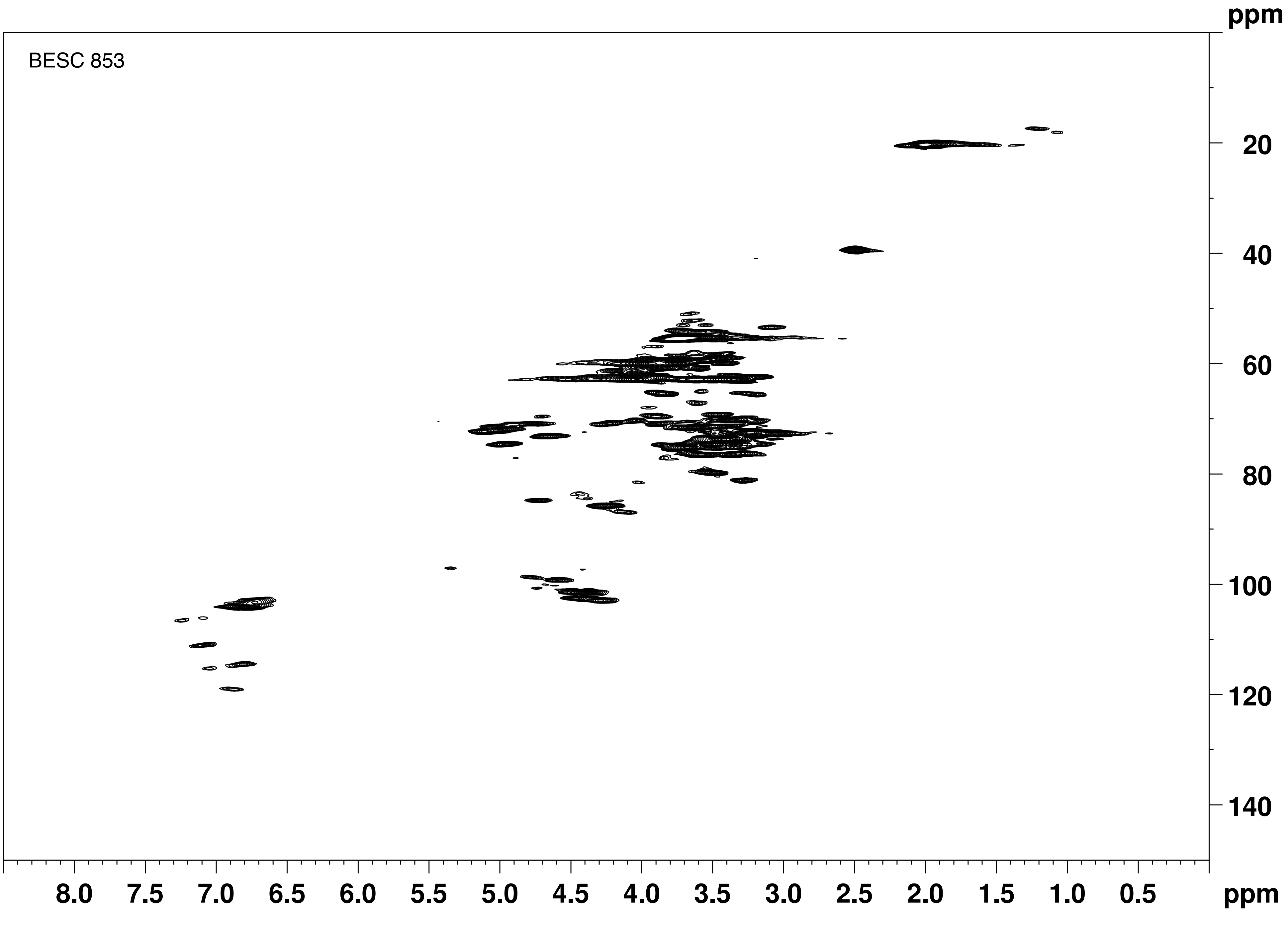

BESC 853
ppm
20
40
60
80
100
120
140
8.0 7.5 7.0 6.5 6.0 5.5 5.0 4.5 4.0 3.5 3.0 2.5 2.0 1.5 1.0 0.5
ppm

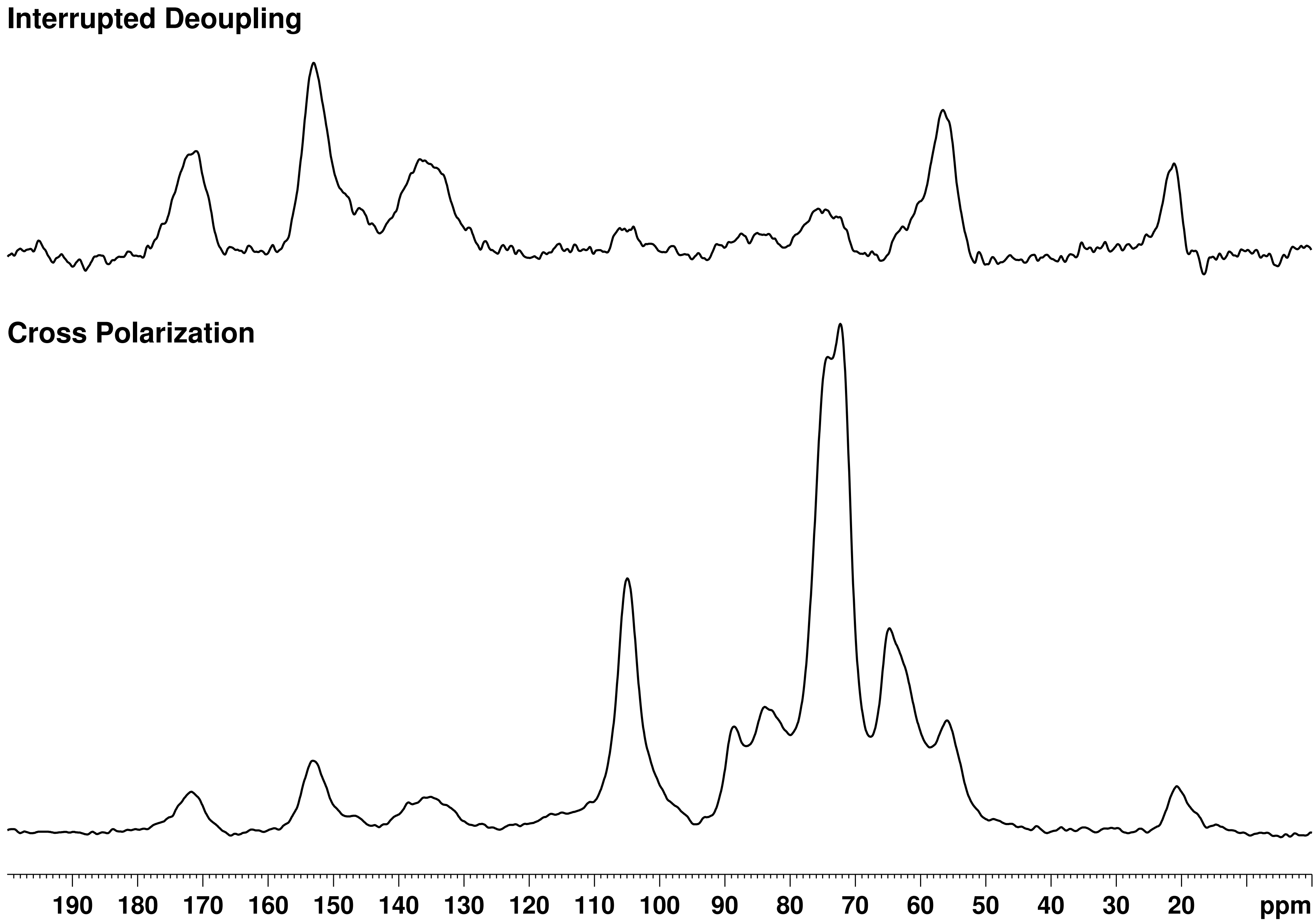
Interrupted Deoupling
Cross Polarization
190 180 170 160 150 140 130 120 110 100 90 80 70 60 50 40 30 20 ppm

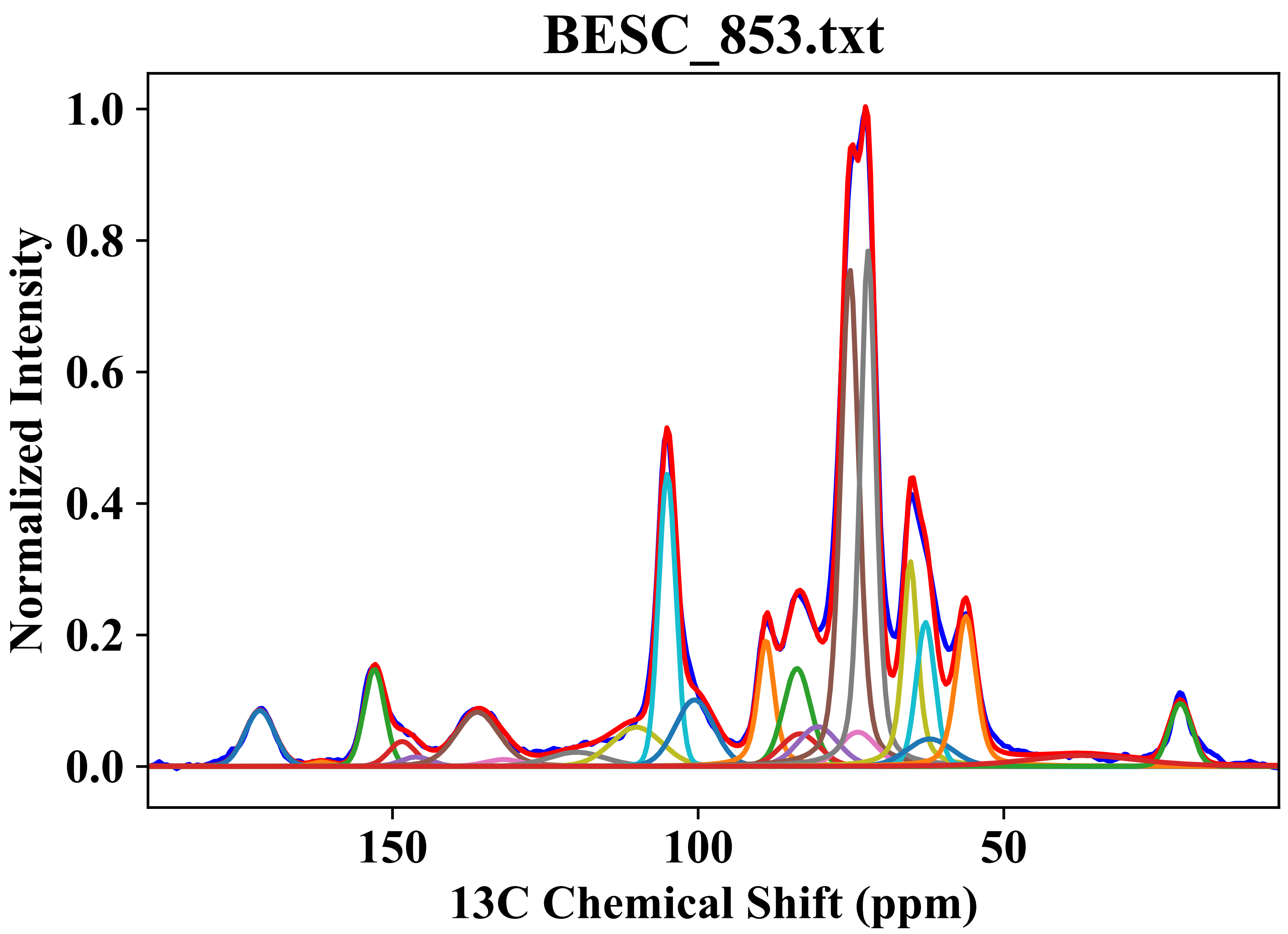
BESC_853.txt
Normalized Intensity
0.0
0.2
0.4
0.6
0.8
1.0
150
100
50
13C Chemical Shift (ppm)

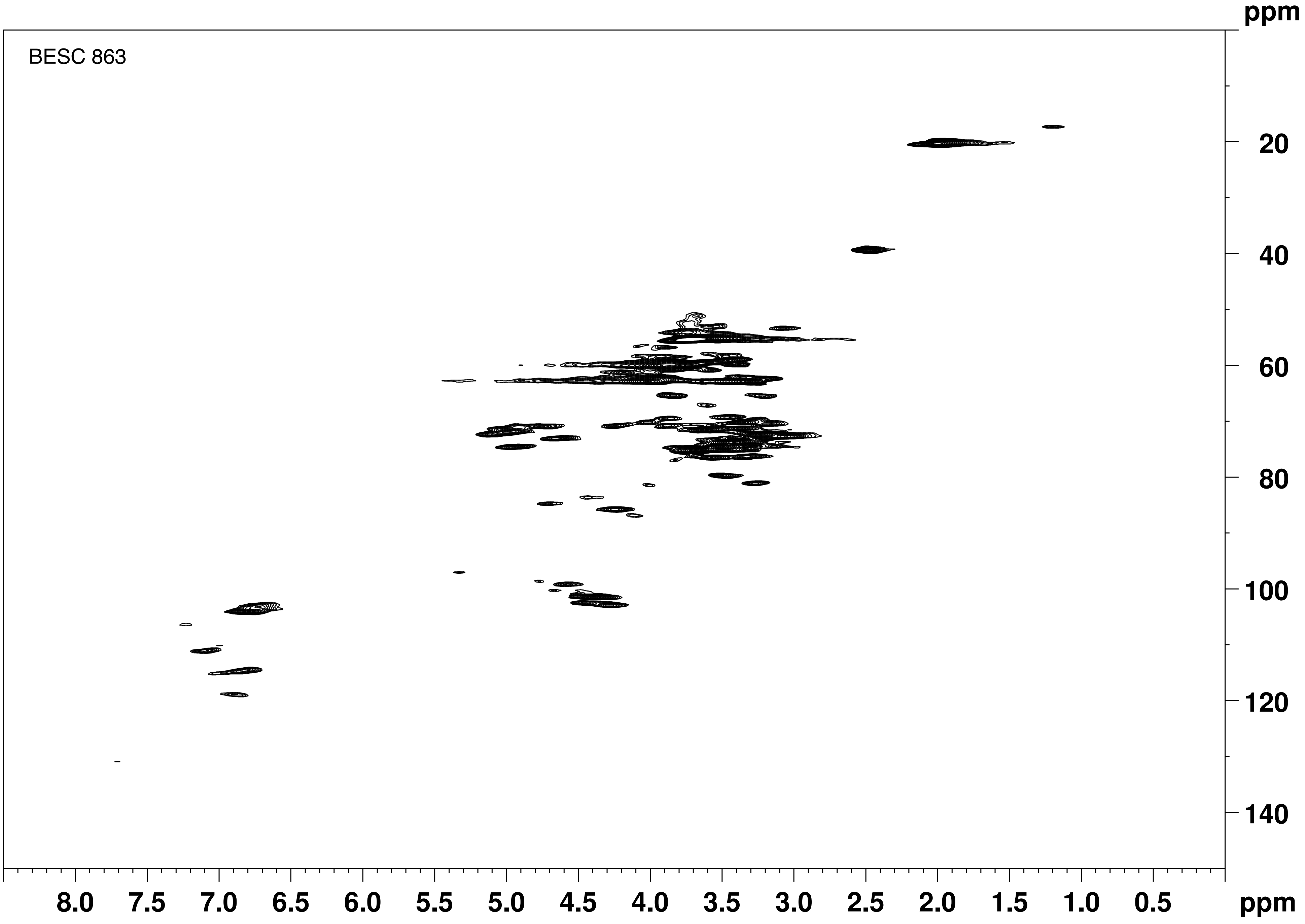

BESC 863
ppm
20
40
60
80
100
120
140
8.0 7.5 7.0 6.5 6.0 5.5 5.0 4.5 4.0 3.5 3.0 2.5 2.0 1.5 1.0 0.5
ppm

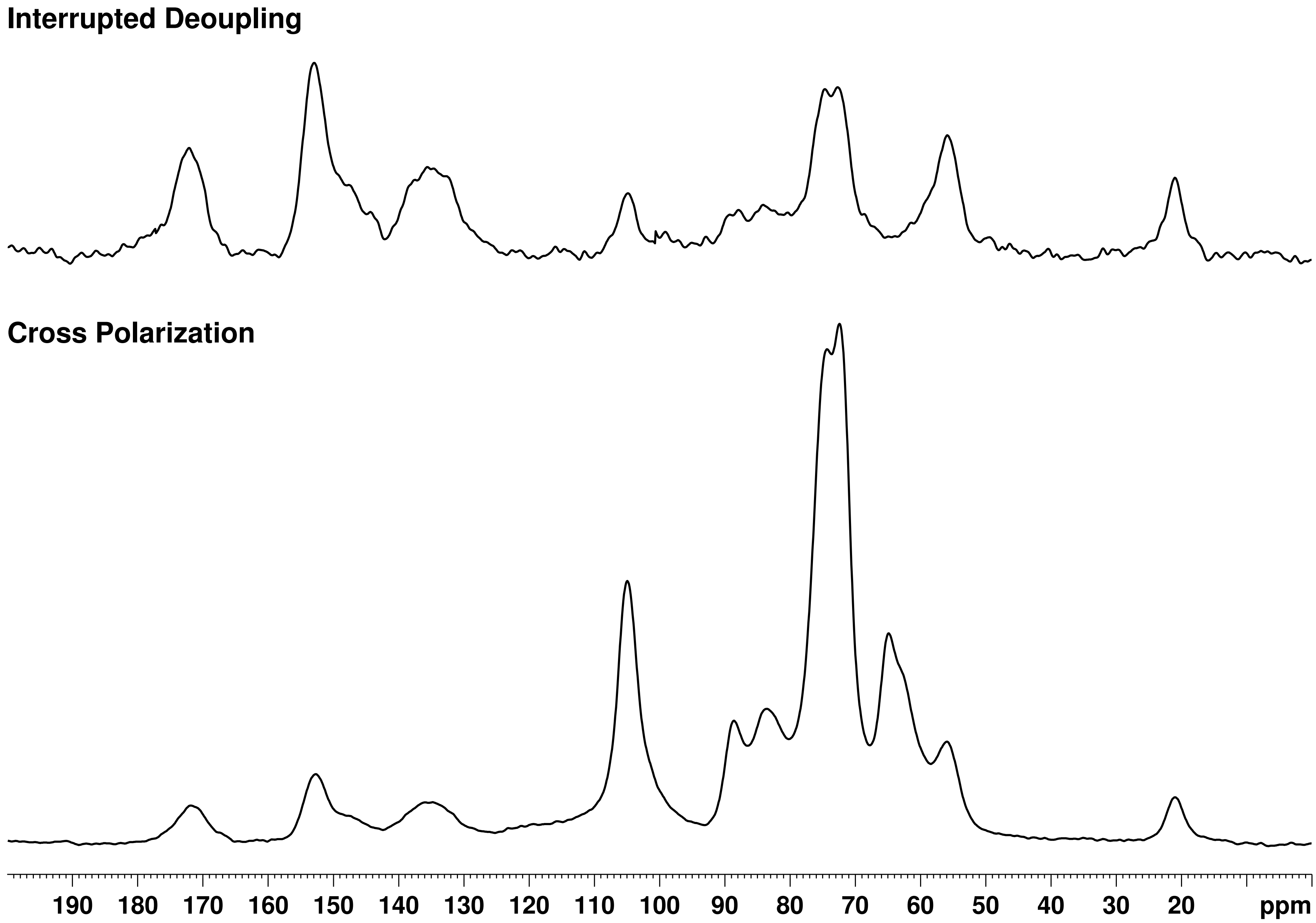
Interrupted Deoupling
Cross Polarization
190 180 170 160 150 140 130 120 110 100 90 80 70 60 50 40 30 20 ppm

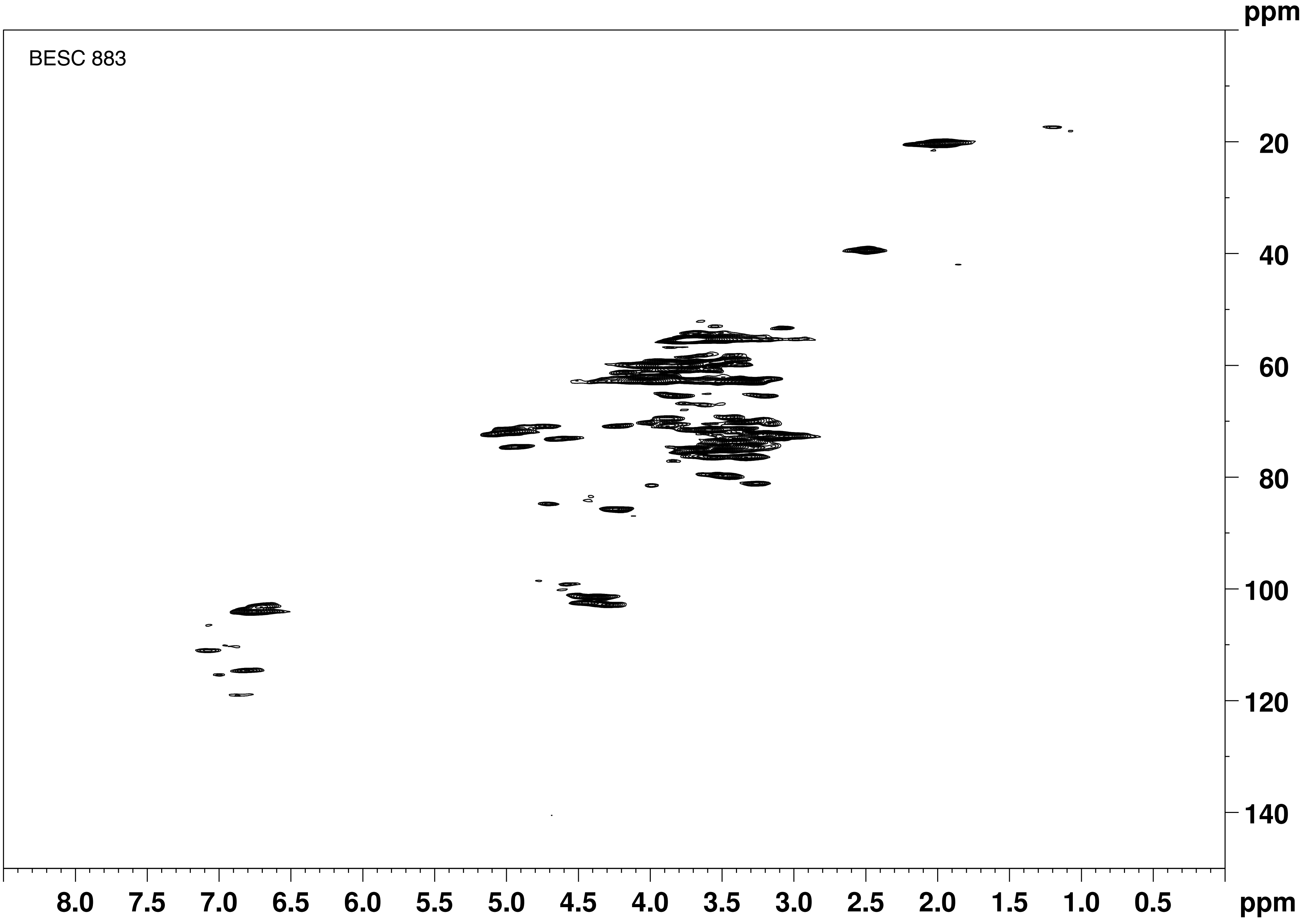

BESC 883
ppm
20
40
60
80
100
120
140
8.0 7.5 7.0 6.5 6.0 5.5 5.0 4.5 4.0 3.5 3.0 2.5 2.0 1.5 1.0 0.5
ppm

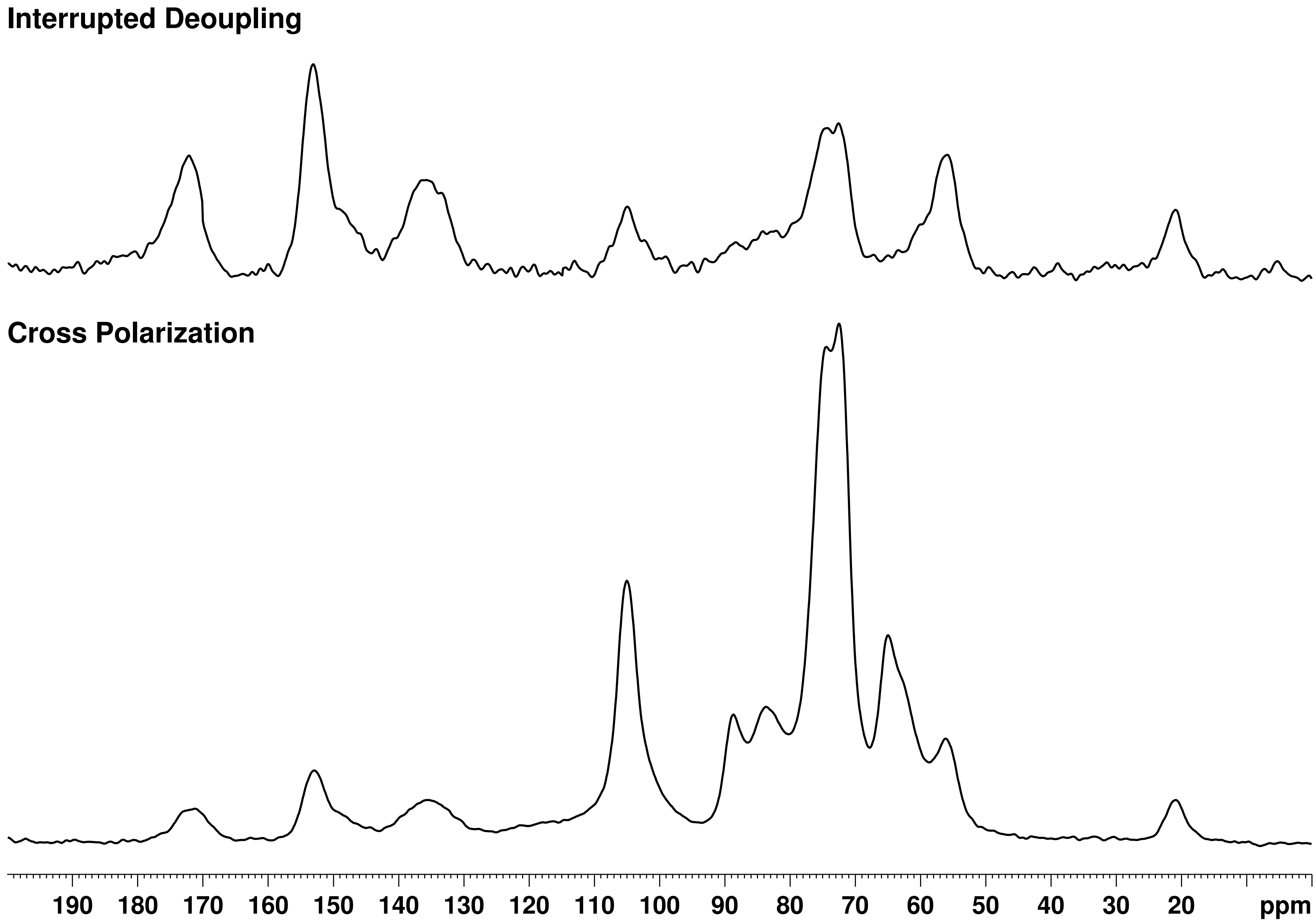
Interrupted Deoupling
Cross Polarization
190 180 170 160 150 140 130 120 110 100 90 80 70 60 50 40 30 20 ppm

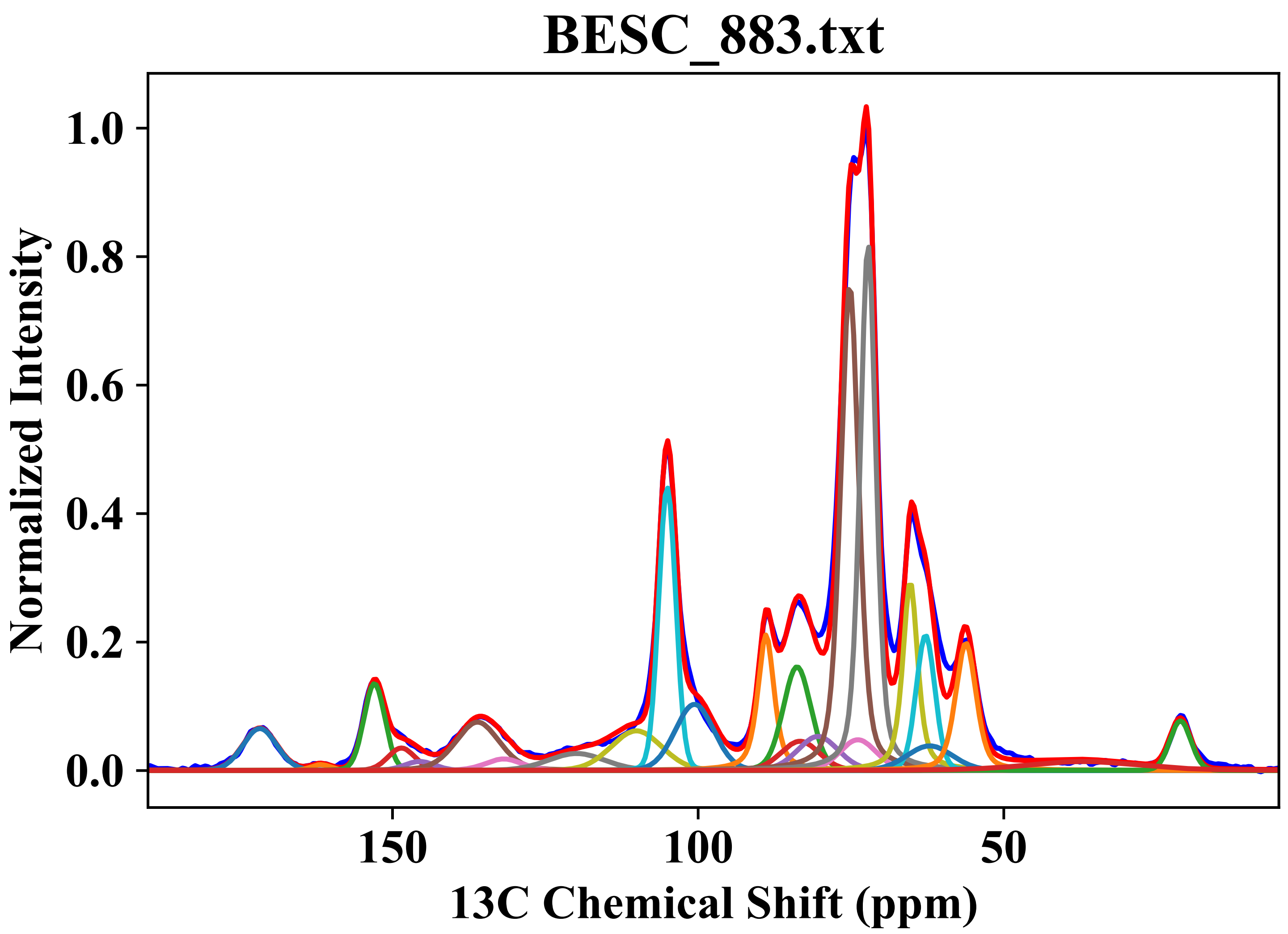
BESC_883.txt
Normalized Intensity
1.0
0.8
0.6
0.4
0.2
0.0
150
100
50
13C Chemical Shift (ppm)

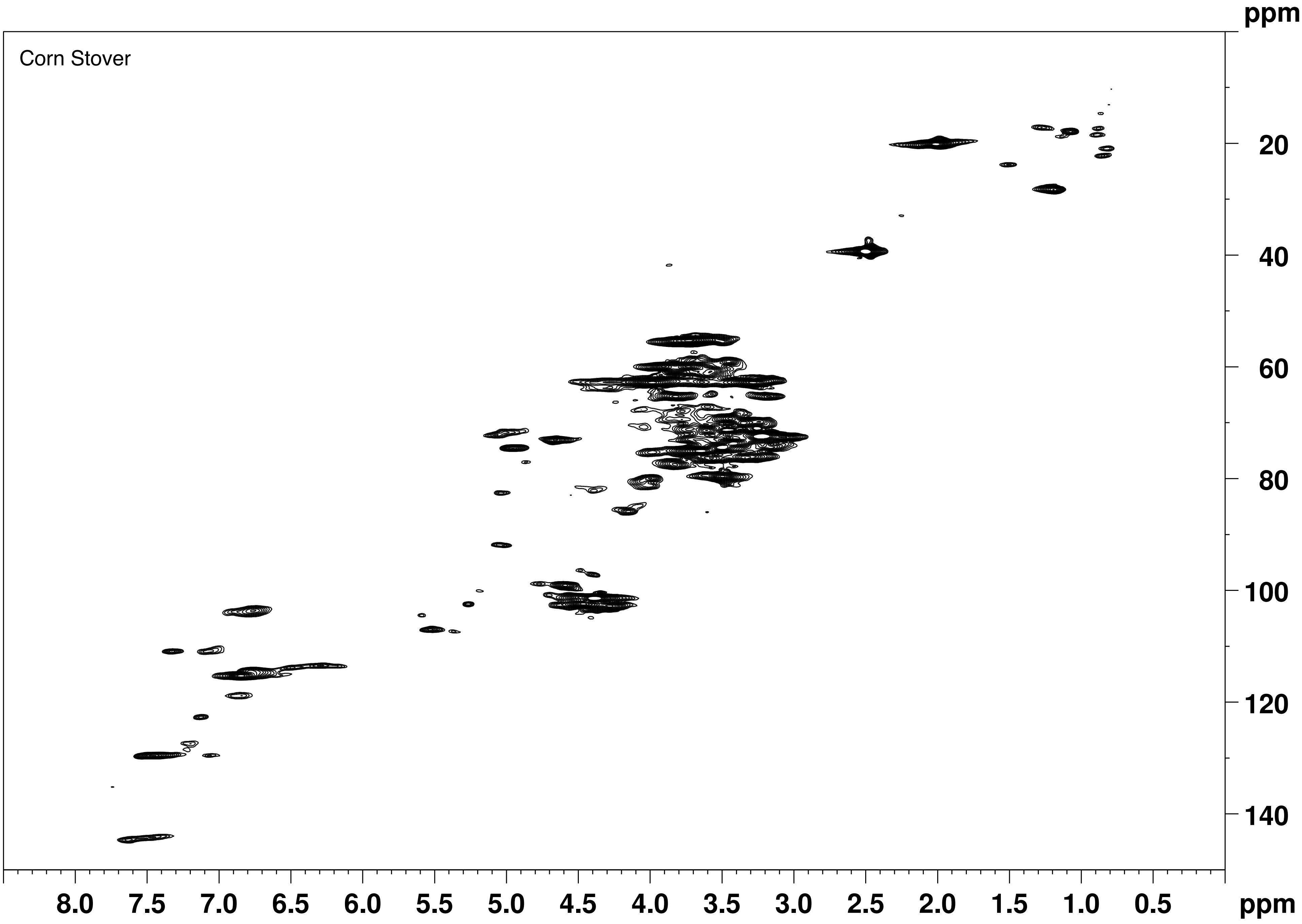

Corn Stover
ppm
20
40
60
80
100
120
140
8.0
7.5
7.0
6.5
6.0
5.5
5.0
4.5
4.0
3.5
3.0
2.5
2.0
1.5
1.0
0.5
ppm

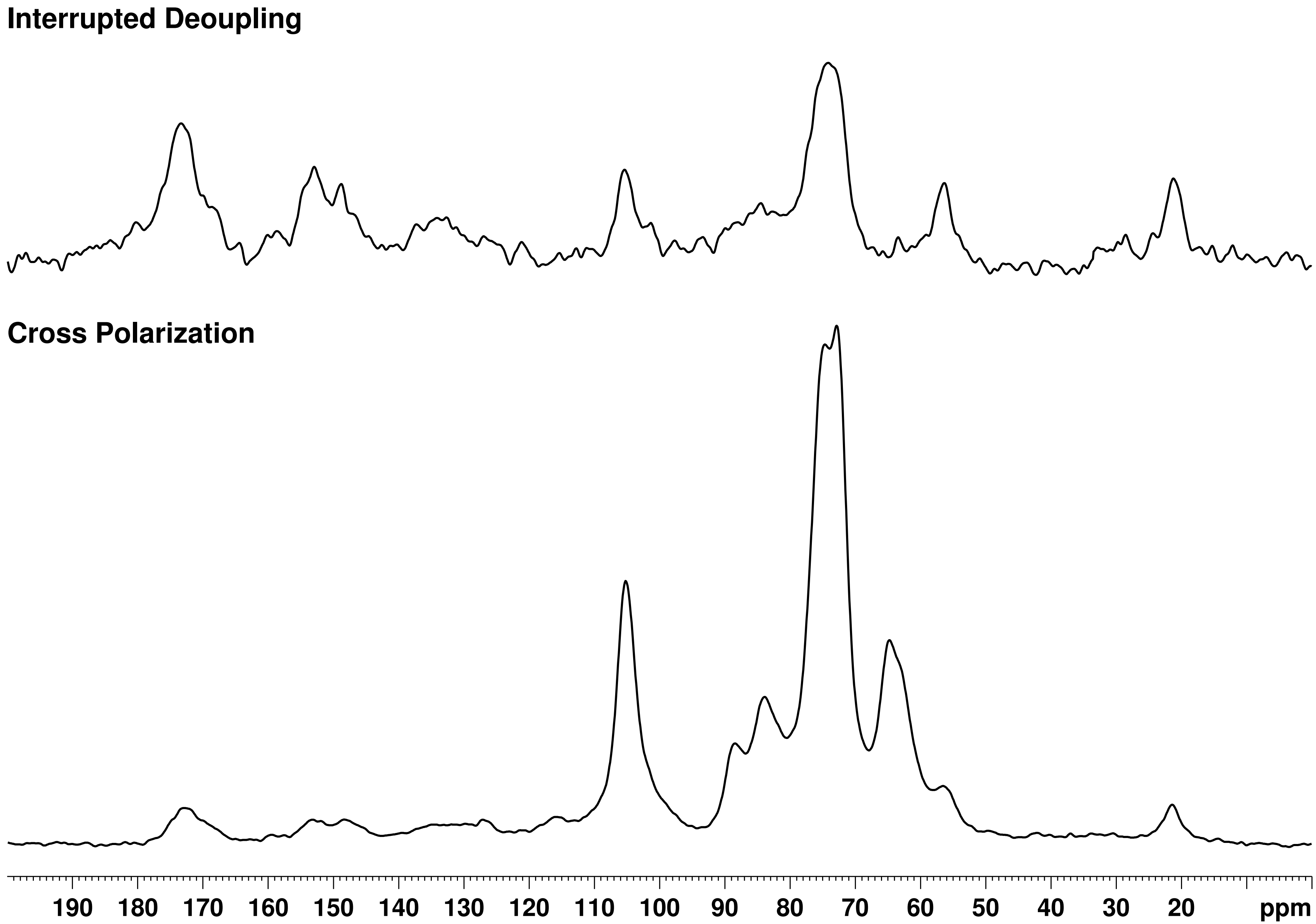

Interrupted Deoupling
Cross Polarization
190 180 170 160 150 140 130 120 110 100 90 80 70 60 50 40 30 20 ppm

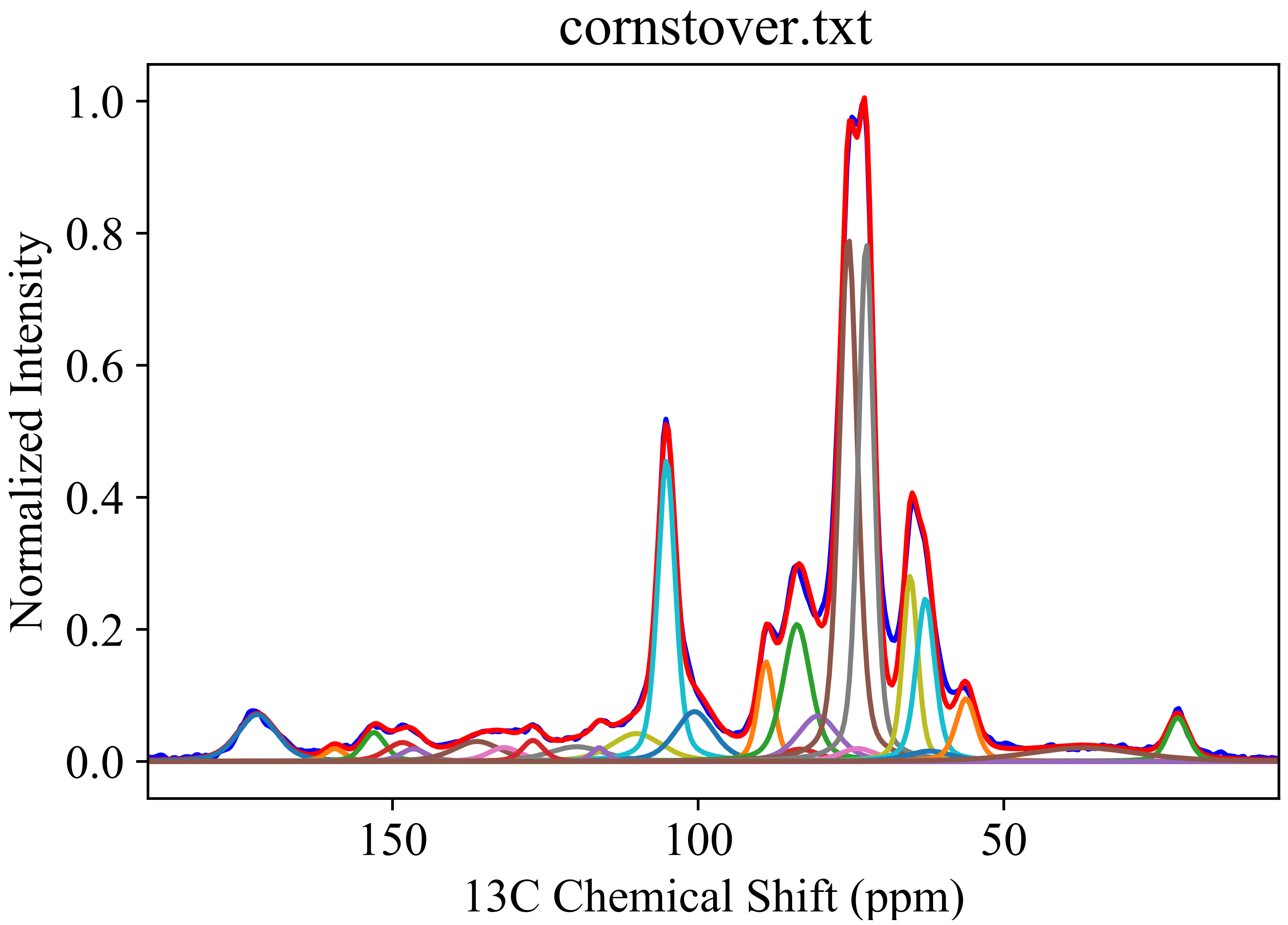

cornstover.txt
Normalized Intensity
0.0
0.2
0.4
0.6
0.8
1.0
150
100
50
13C Chemical Shift (ppm)

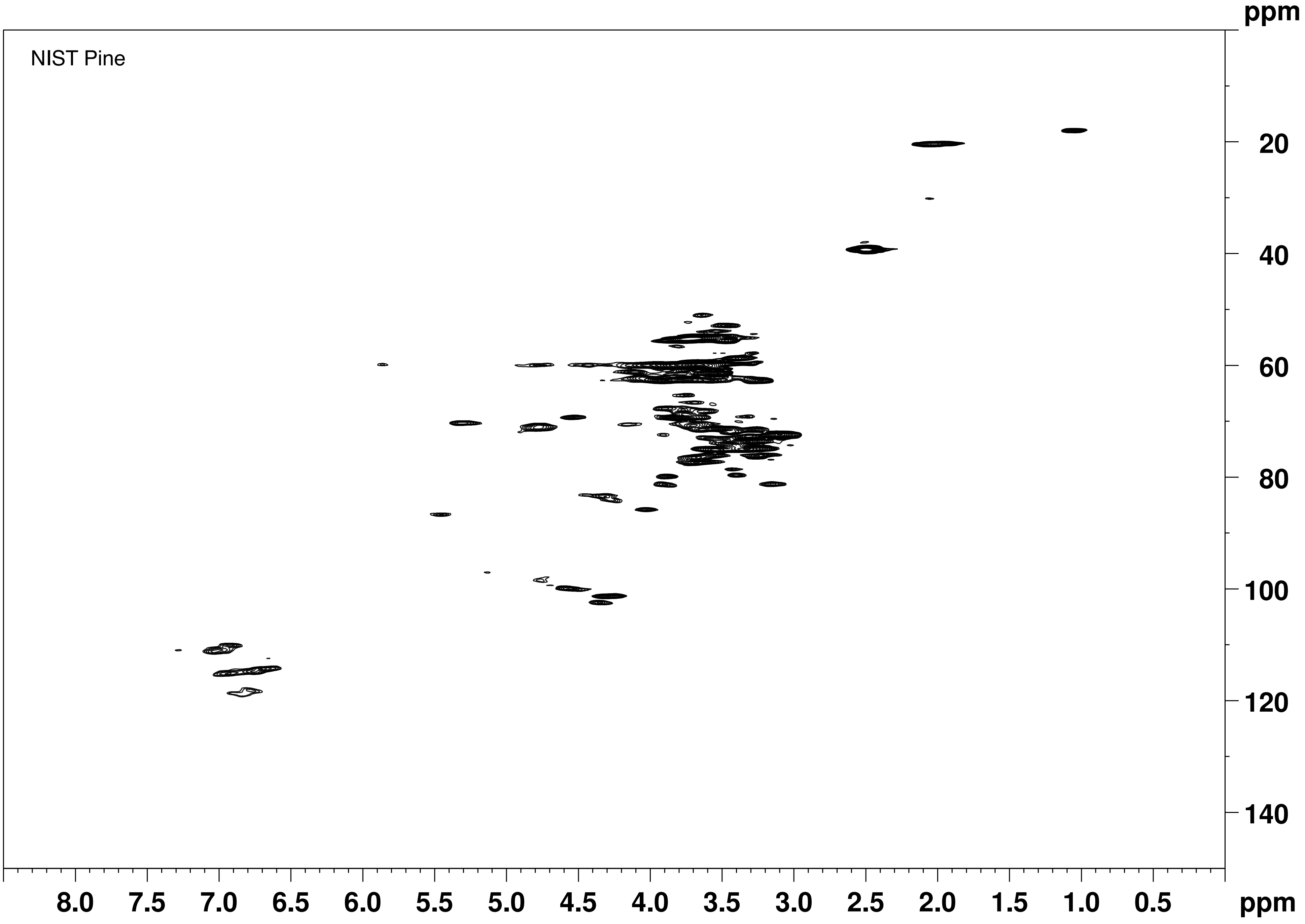
NIST Pine
ppm
20
40
60
80
100
120
140
8.0
7.5
7.0
6.5
6.0
5.5
5.0
4.5
4.0
3.5
3.0
2.5
2.0
1.5
1.0
0.5
ppm

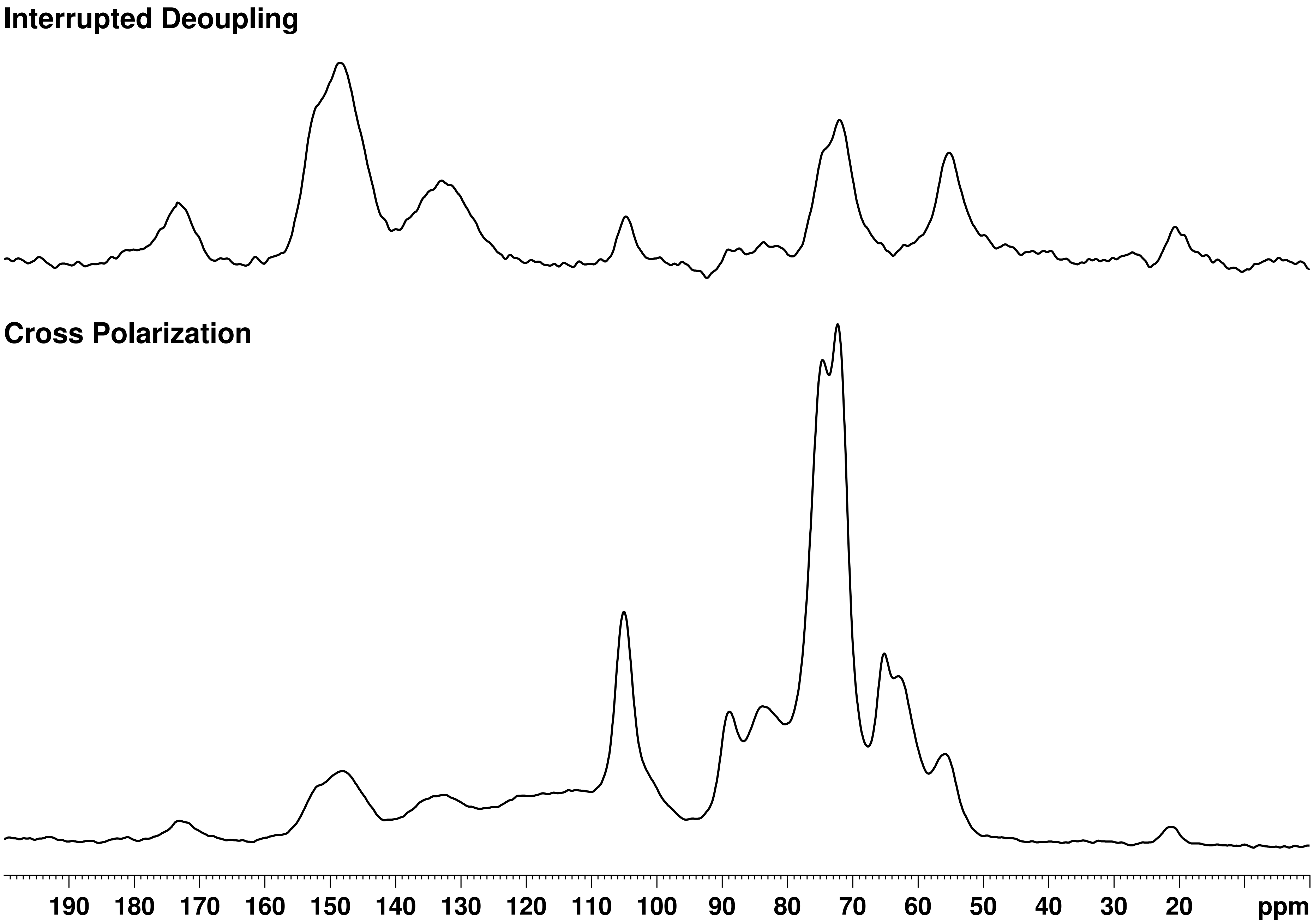
Interrupted Deoupling
Cross Polarization
190 180 170 160 150 140 130 120 110 100 90 80 70 60 50 40 30 20 ppm
